# Total Synthesis of Parameritannin A2, a Branched Epicatechin Tetramer with Two Double Linkages

**DOI:** 10.1002/anie.202205106

**Published:** 2022-05-20

**Authors:** Vipul V. Betkekar, Keisuke Suzuki, Ken Ohmori

**Affiliations:** ^1^ Department of Chemistry Tokyo Institute of Technology 2-12-1, O-okayama Meguro-ku, Tokyo 152-8551 Japan

**Keywords:** Cascade Reactions, Flavonoid, Oligomer, Polyphenol, Pummerer Reaction

## Abstract

The first total synthesis of parameritannin A2 (**1**), a branched epicatechin (EC) tetramer is reported. The “phloroglucinol trick” was used to circumvent two synthetic issues encountered when assembling four EC units, namely, the steric constraint and the formation of the C4−C6 interflavan linkage. As a substructure of the middle EC unit, phloroglucinol enabled the single‐step assembly of two EC units (top and side) through A‐type linkages. The middle EC unit was constructed by conducting a newly developed three‐carbon flavan annulation via a Pummerer/Friedel–Crafts cascade reaction to furnish a trimeric intermediate bearing a thio‐leaving group at C4 position, which allowed the final installation of the bottom EC unit.

## Graphical abstract

By overcoming steric constraints, a stereoselective total synthesis of the branched tetramer parameritannin A2 was achieved. As a key step of the approach, a Pummerer/Friedel–Crafts cascade enabled the de novo construction of the middle flavan unit for its regioselective linkage to the bottom flavan unit.

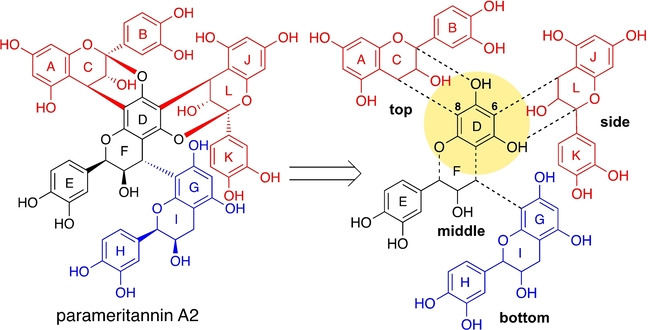

## Introduction

**Figure 1:**
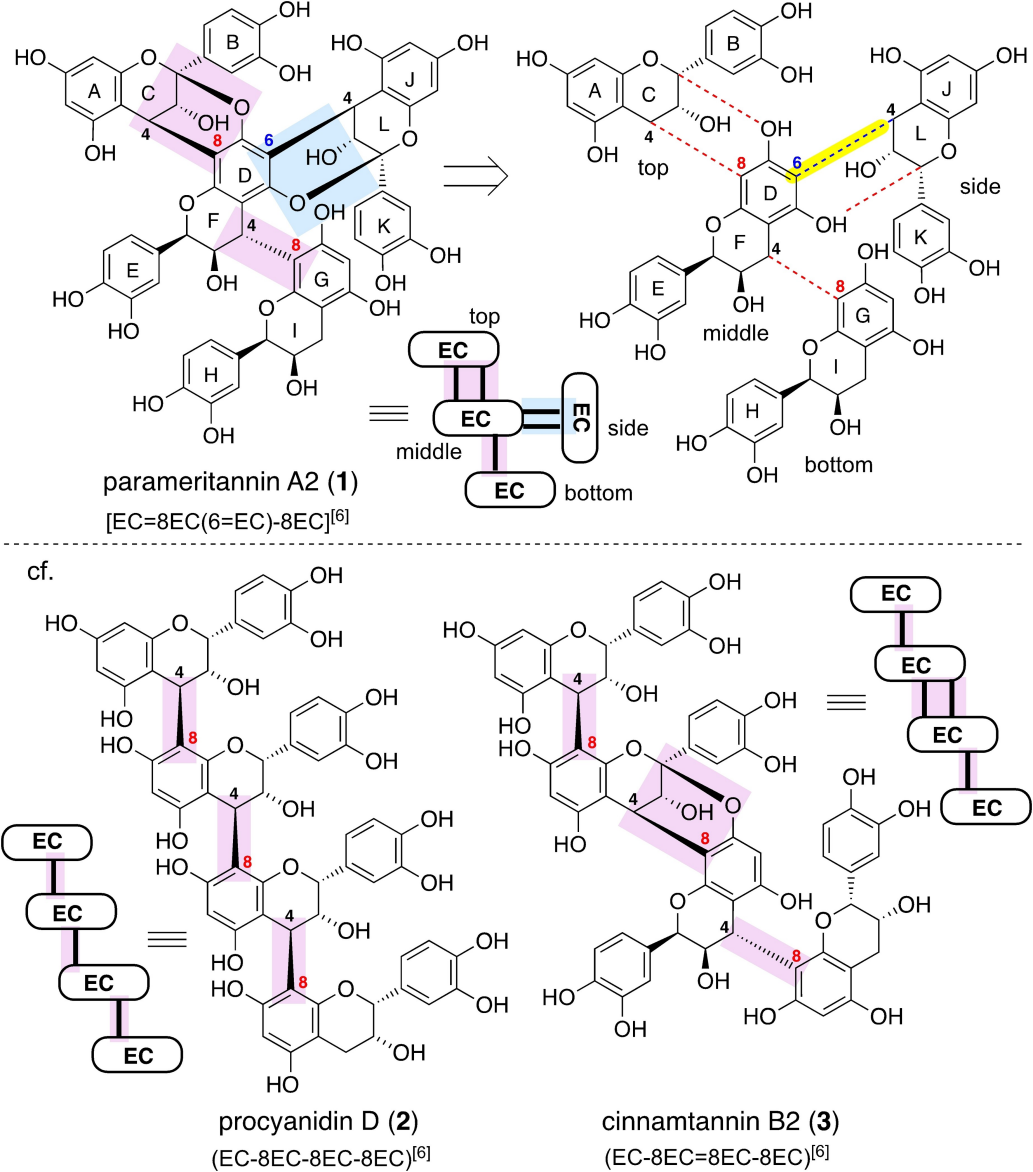
Structures of parameritannin A2 (**1**) as a branched OPA and procyanidin D (**2**) and cinnamtannin B2 (**3**) as a linear‐type OPA. EC=epicatechin.

Parameritannin A2 (**1**) is an epicatechin (EC) tetramer that is isolated from the bark extracts of the Asian traditional folk medicinal plant *Parameria laevigata* Moldenke along with several other EC oligomers (Figure 1).[^1^, ^2^] Compound **1** exhibits a unique branched structure, in which three EC units (top, bottom, and side) are convergently linked to a single EC unit (middle). This structure contrasts with the linear array of EC units shared by many flavan oligomers, such as procyanidin D (**2**) and cinnamtannin B2 (**3**), which differ in the absence or presence of an A‐type double linkage.^[3]^

In our synthetic studies on oligomeric proanthocyanidins (OPAs), we previously completed the total syntheses of linear‐type OPAs with or without a double linkage[^4^, ^5^] and turned our attention to the synthesis of **1**. We envisaged two potential issues in the construction of the branched structure of **1**, i.e., 1) the steric constraint in the assembly of three EC units to the middle EC unit and 2) the lack of a reliable approach for the formation of the C4−C6 interflavan bond.

Among the three interflavan linkages in **1**, the top‐middle and middle–bottom connections (pink) involve a C4−C8 bond, for which we could rely on our previously established methods (vide infra). However, the formation of the middle‐side linkage (blue) involved a more challenging C4−C6 interflavan bond (yellow).^[6]^

**Scheme 1:**
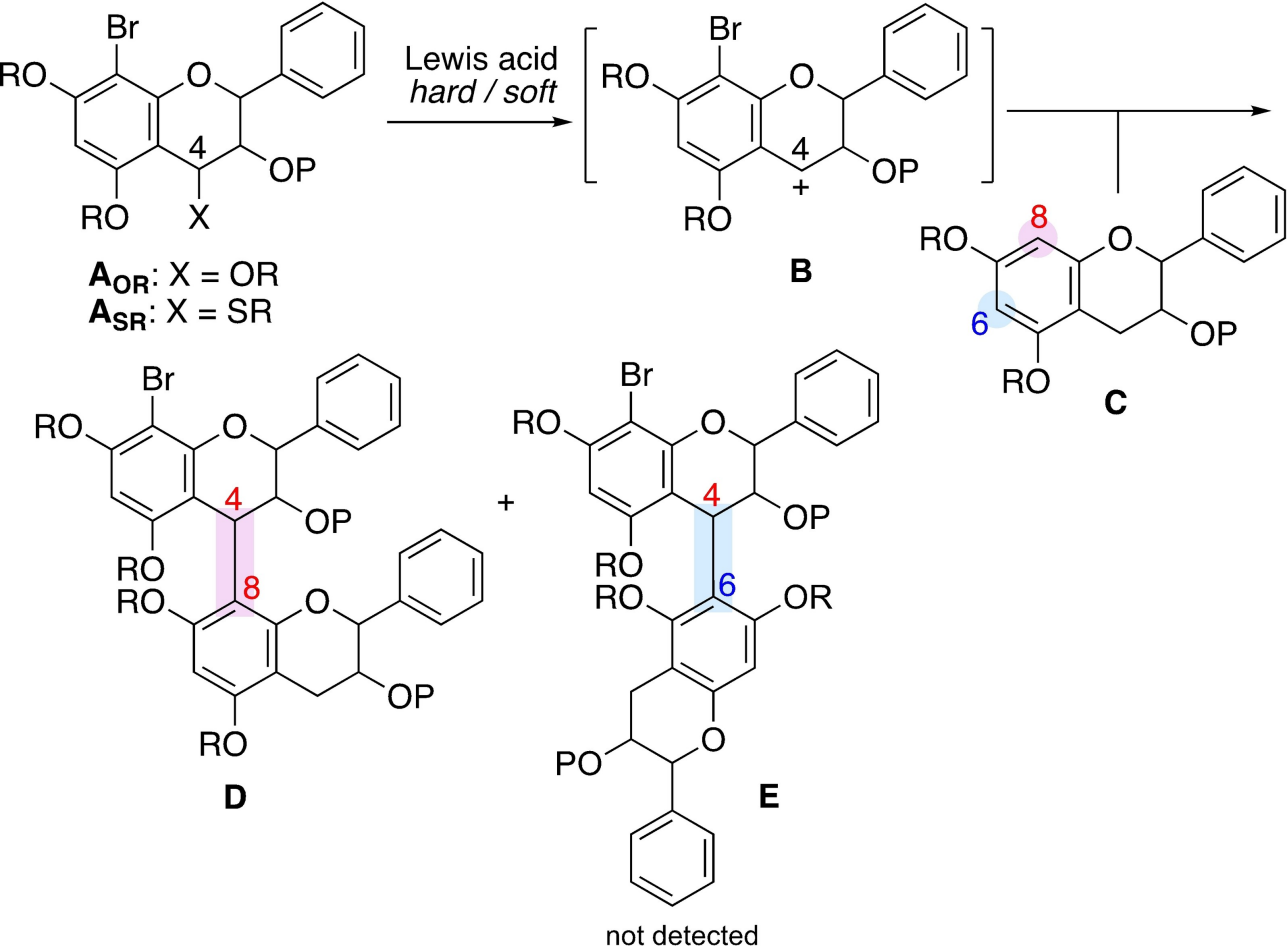
Single (B‐type) interflavan bond formation.

Scheme 1 shows our previously developed methods for single bond formation via the C4 cation intermediate **B**, which is generated upon activation with hard and soft Lewis acids of catechin units **A_OR_** and **A_SR_** that contain oxy and thio moieties as leaving groups, respectively.[^4^, ^5^] Importantly, cation **B** is intercepted by nucleophilic unit **C** at the C8 position rather than at the C6 position. Thus, the C4−C8 interflavan single bond is easily and preferentially formed over the C4−C6 bond.[^7^, ^8^]

**Scheme 2:**
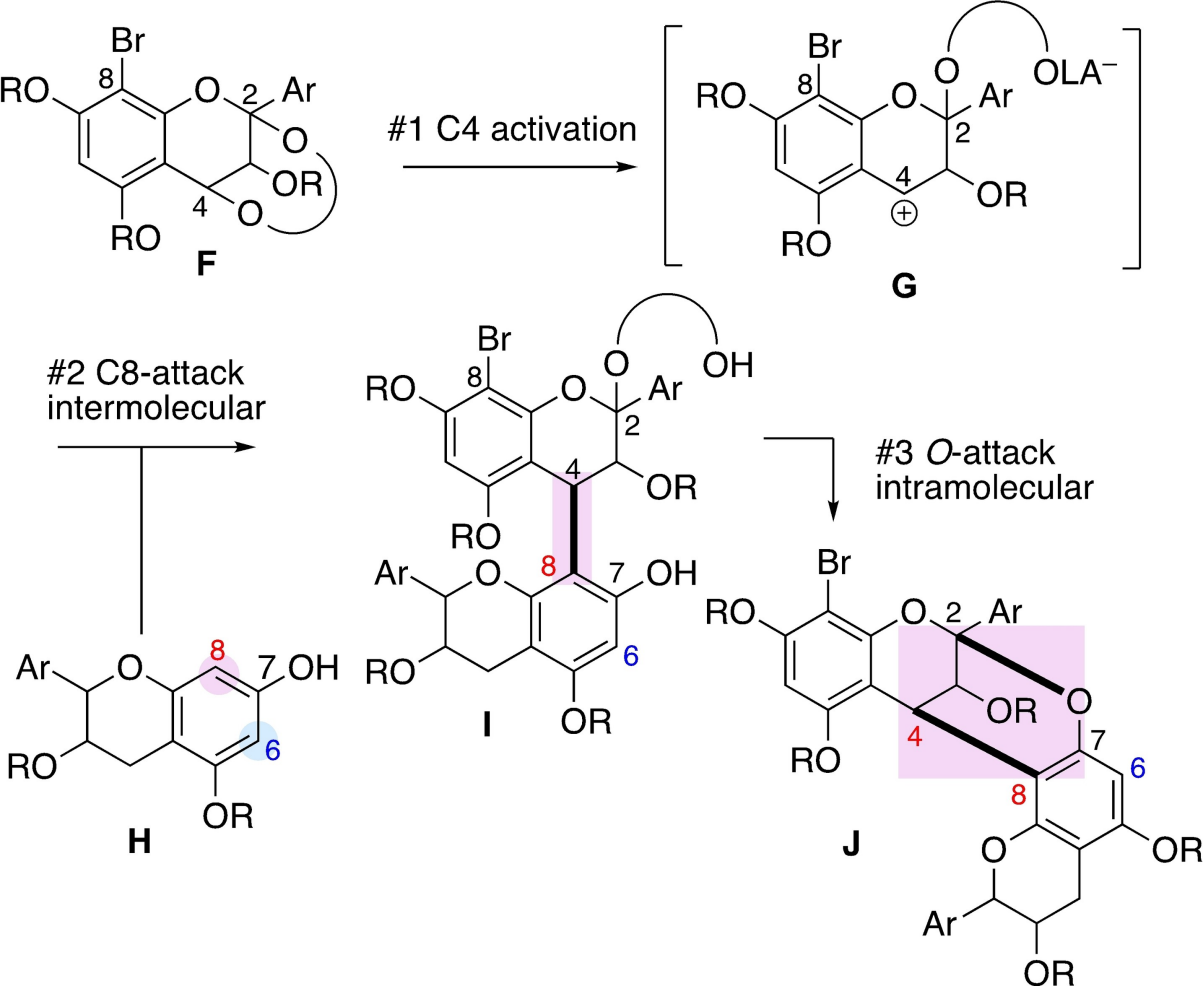
Dual (A‐type) interflavan bond formation.

The same trend applies to the A‐type linkages. Accordingly, a dual linkage involving a C4−C8 bond is accessible, but not a C4−C6 bond (Scheme 2).[^5^, ^7^] This trend has a mechanistic basis. Thus, the regioselectivity is determined at the C−C bond‐forming stage, in which two flavan units are connected at the C4 and C8 positions. Upon exposure to acid, 2,4‐dioxy‐substrate **F** initially generates C4 cation **G** (stage #1), which is then trapped by nucleophilic unit **H** at its C8 position (stage #2), forming the C4−C8 linked intermediate **I**. The second activation leads to the formation of an internal C−O bond (stage #3), giving A‐type dimer **J** containing a C4−C8 linkage.

## Results and Discussion

Our first attempts at accomplishing the challenging formation of the C4−C6 bond for the synthesis of **1** were unsuccessful. However, by adopting a strategy called “the phloroglucinol trick,” we achieved the first total synthesis of **1**, which is described in this article.

**Scheme 3:**
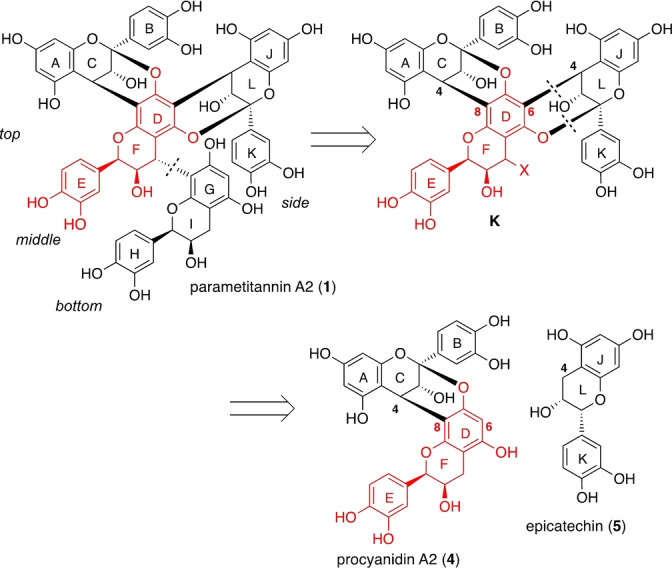
Initial retrosynthesis.

Scheme 3 depicts the initially attempted retrosynthesis of **1**, starting with the disconnection of the bottom EC unit to trimer **K**, followed by a second disconnection of the side EC unit, which suggested procyanidin A2 (**4**) as a dimeric progenitor recently synthesized by our group.[^5a^, ^7^]

**Scheme 4:**
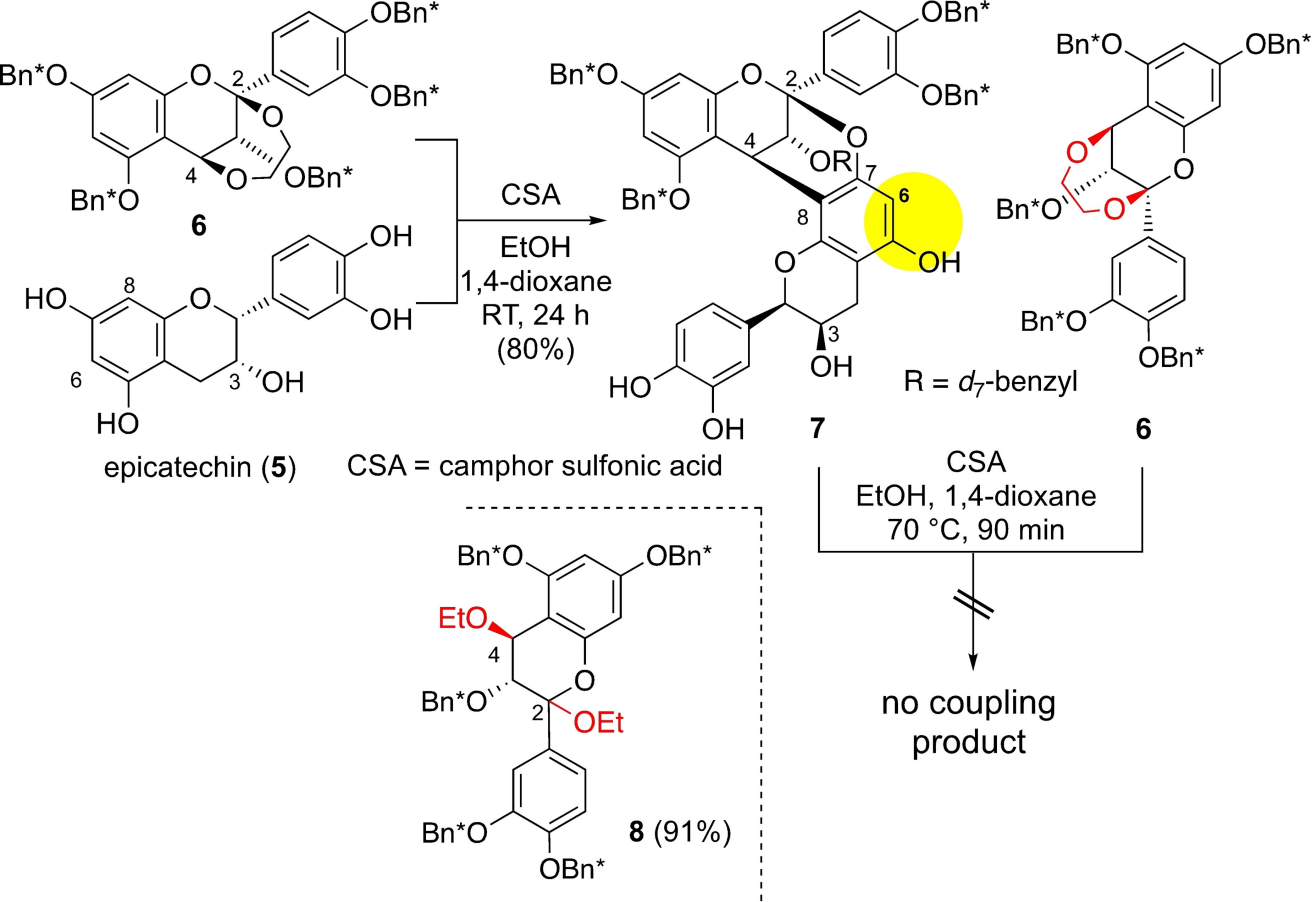
Attempted annulation of A‐type dimer **7** and dication progenitor **6**.

Scheme 4 shows the synthesis of **4** starting from *free* EC (**5**) as a nucleophilic unit.^[7]^ Despite the presence of multiple potential reaction sites in **5**, regioselective annulation occurred, furnishing the C4−C8 linked A‐type dimer **7** as the main product. It should be noted that dimer **7**, as a suitably protected form of **4**, could be envisaged as a promising intermediate bearing the appropriate nucleophilic reaction sites (yellow) for the second annulation with **6** to afford the branched trimer **K**.

Therefore, we examined a camphor sulfonic acid (CSA)‐promoted reaction of dimer **7** with dication progenitor **6**.^[9]^ Unfortunately, although the starting material **6** was completely consumed, no coupling product **K** was obtained even after heating at 70 °C for 1.5 h. Instead, diethoxy derivative **8** was produced (91 % yield), indicating the generation of a cationic species from **6**, which was nonetheless not attacked by the C6 nucleophilic center in **7**. This failure could be ascribed to the steric hindrance that prevented the formation of a hexasubstituted benzene or to the intrinsically poor reactivity of the C6 site of the flavan skeleton in **7**.

**Scheme 5:**
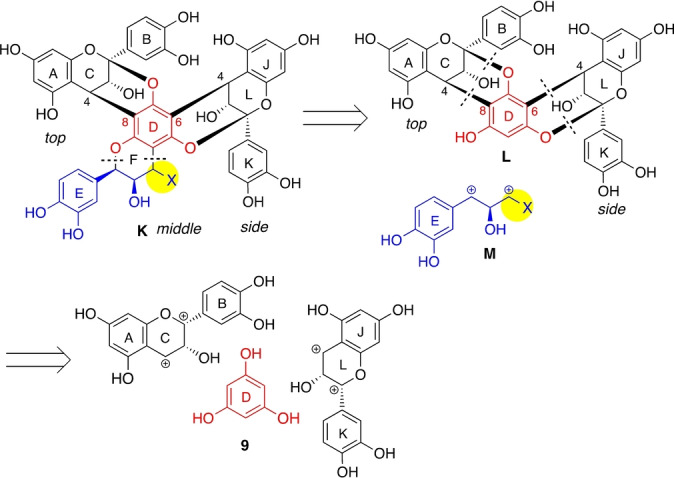
Alternative retrosynthesis.

To circumvent this issue, we turned our attention away from the traditional disconnections and considered further fragmentation of the middle unit. As shown in the alternative retrosynthesis depicted in Scheme 5, a two‐bond disconnection of **K** would lead to precursor **L** and the three‐carbon dication synthon **M**. Furthermore, the presence of a structural motif of phloroglucinol (**9**) in **K** suggested the feasibility of the disconnection of two EC units.

This synthetic plan required addressing two problems, i.e., 1) a double A‐type flavan annulation onto **9** and 2) the de novo construction of the middle EC unit by combining **L** with synthon **M** having a leaving group X at C4 position (yellow, Scheme 5).

**Scheme 6:**
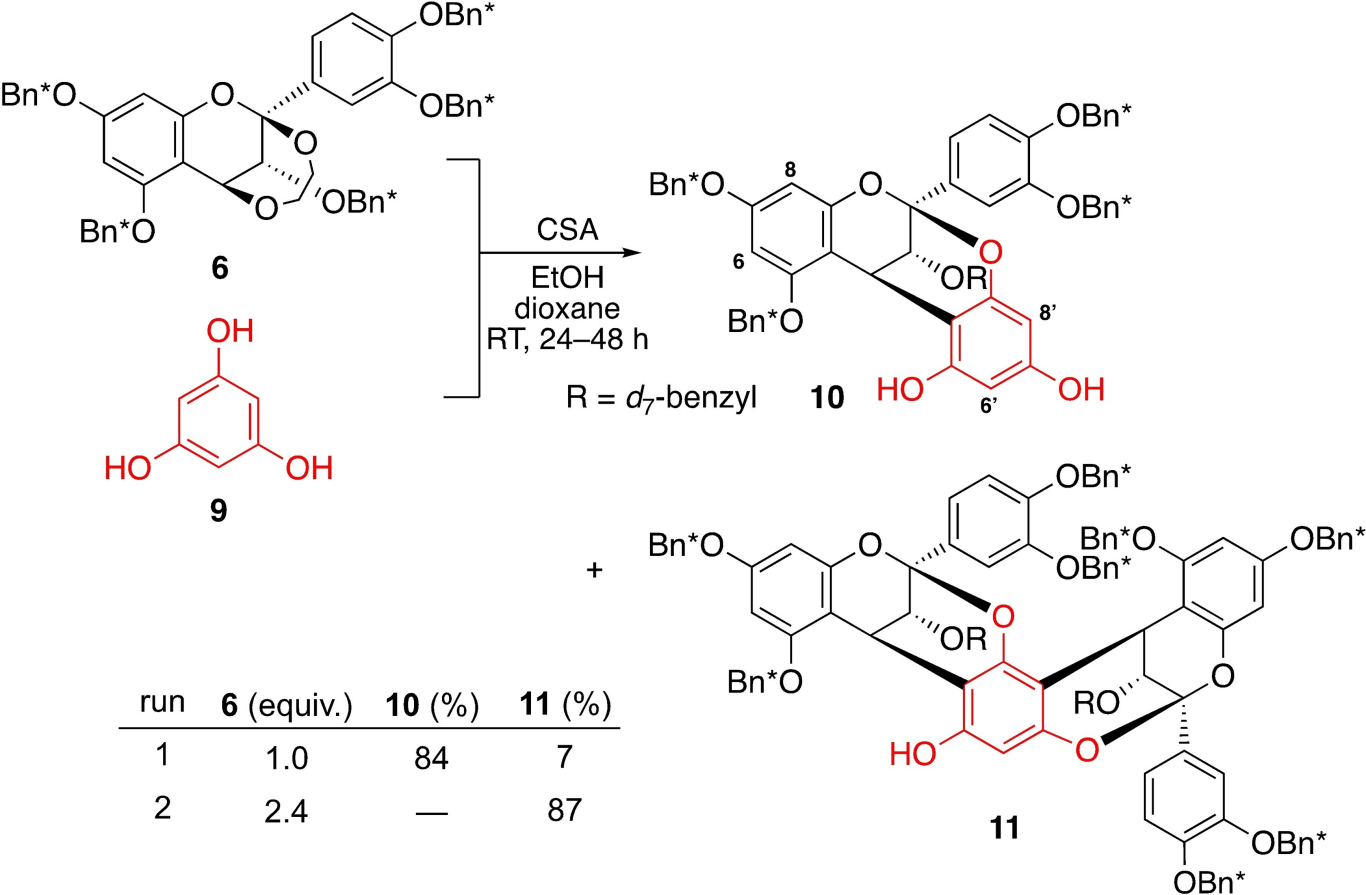
Bisannulation.

To test the reactivity of dioxy‐flavan **6**, we evaluated its reaction with phloroglucinol (**9**) as a model reaction (Scheme 6). The CSA‐promoted reaction of **9** and **6** in 1/1 ratio cleanly gave monoannulation product **10**.^[7]^ Interestingly, a trace amount of bisannulation product **11** was also isolated, whose formation in a higher yield would endorse the synthetic plan stated above. Pleasingly, a simple change in the molar ratio of **6** and **9** to 2.4/1 improved the yield of bisannulation product **11** to 87 %, which was achieved via **10** through a second annulation.

**Scheme 7:**
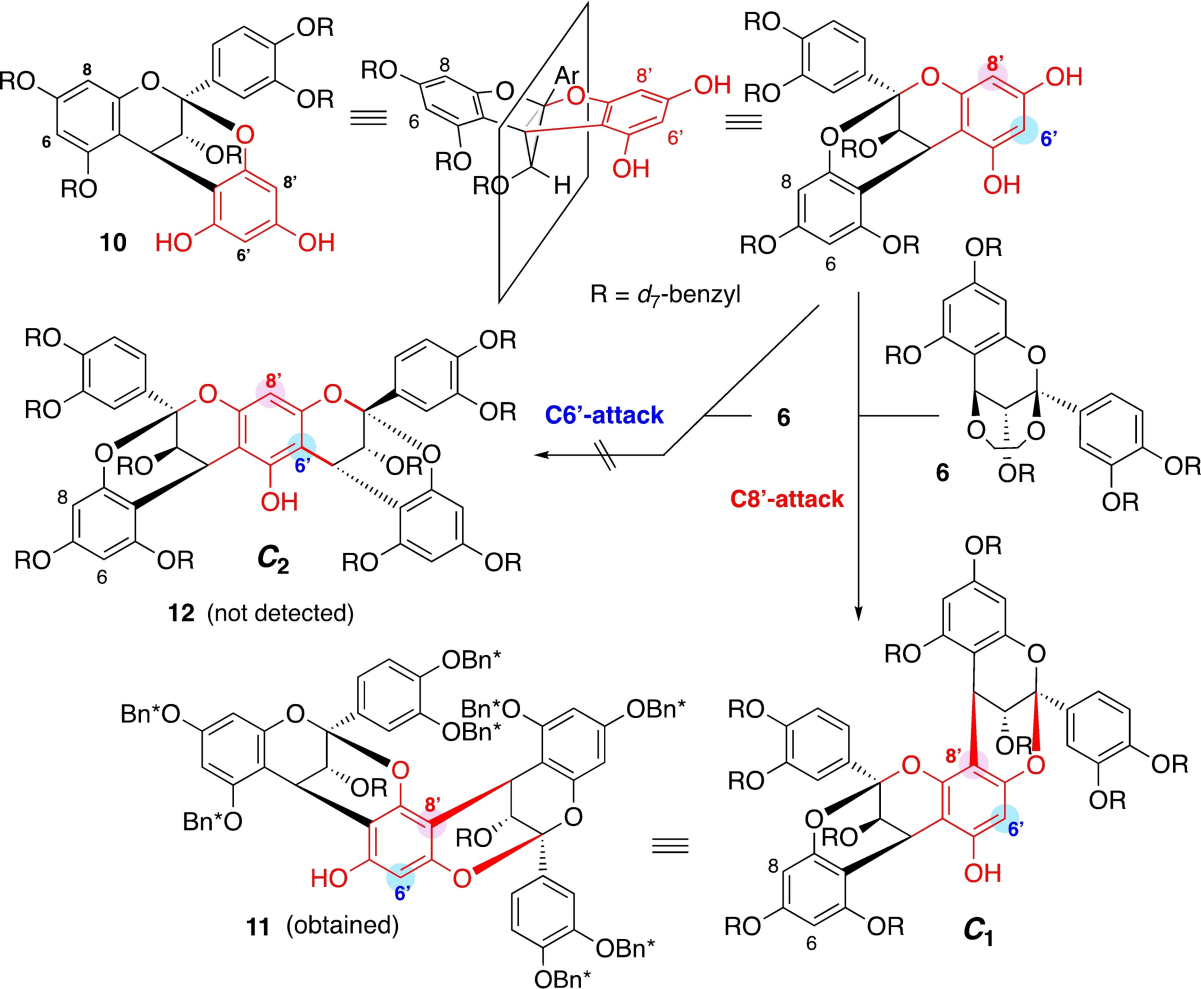
Exposure of the phloroglucinol trick for circumventing the C4−C6 bond‐forming issue.

With this positive result in hand, the reason why the second annulation proceeded with **10** but not with **7** (vide supra) was analyzed. Notably, intermediate **10** exhibited an intriguing structural feature that could be exploited to circumvent the C4−C6 bond‐forming issue (Scheme 7). Specifically, **10** contains two flavan skeletons, i.e., one originates from **6** (black), and the other (red) is an *artifact* generated by the annulation with **9**. The latter moiety (red) has two free phenols and two possible nucleophilic carbon centers at the C8′ and C6′ positions. In fact, the C8′ position underwent a second annulation to give **11**. Interestingly, no *C*_2_ symmetric isomer **12** stemming from the analogous reaction at the C6′ position was detected.

**Scheme 8:**
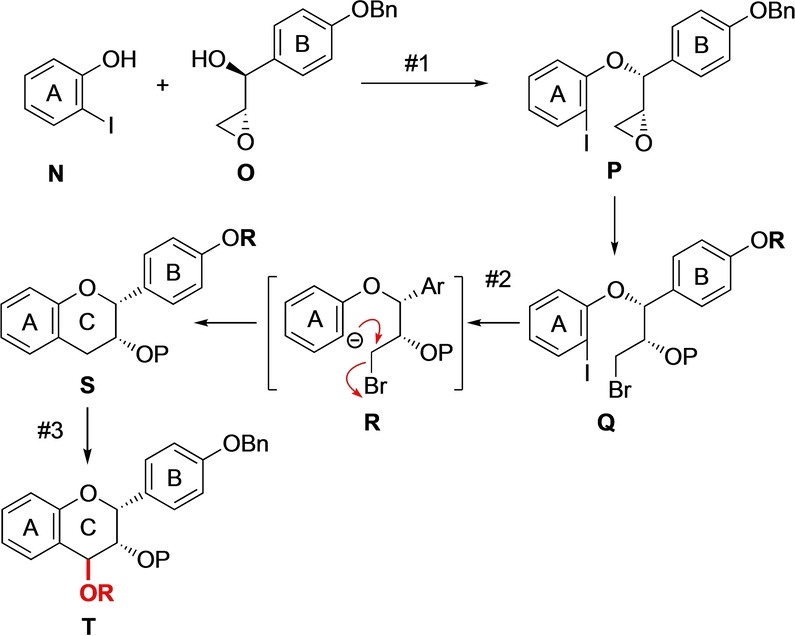
Previous de novo synthetic route to a dimerization‐ready flavan unit.

After the successful union of two A‐type linkages, our next task was to construct the middle EC unit. For this purpose, one of our previous approaches for the de novo flavan synthesis could be considered (Scheme 8).^[10]^ In such an approach, the combination of stereodefined epoxy alcohol **O** with iodophenol **N** via the Mitsunobu reaction produces epoxy ether **P**, which is cleaved to give bromide **Q**. Selective iodine−metal exchange generates anion **R**, which undergoes an internal S_N_2 reaction to give flavan **S**. To provide a reactive site for the interflavan linking, an oxy‐leaving group (red) is then installed at the C4 position in **S** via oxidation by using 2,3‐dichloro‐5,6‐dicyanobenzoquinone (DDQ), giving electrophilic flavan unit **T**.^[11]^

Although this protocol served as a basis for our OPA synthesis, allowing flexible access to various flavan congeners including scarcely available derivatives, we suspected that it might not be effective in the present context due to the challenging installation of a leaving group at C4 position in intermediates having other benzylic C−H bonds more prone to undergo DDQ oxidation.^[12]^

**Scheme 9:**
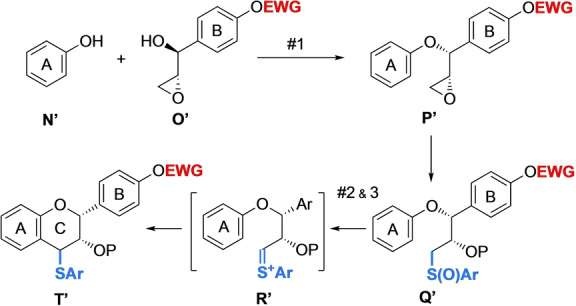
De novo synthetic access to a dimerization‐ready flavan unit. EWG=electron‐withdrawing group.

Therefore, we resorted to performing an alternative reaction to enable direct access to a dimerization‐ready flavan unit. As shown in Scheme 9, this approach would involve the conversion of epoxide **P′** into sulfoxide **Q′**, which would be subjected to Pummerer conditions to afford cationic species **R′**. Finally, the Friedel–Crafts cyclization of **R′** would furnish flavan unit **T′** bearing a thio‐leaving group at C4 position.

To prevent the potential stereochemical erosion in the Mitsunobu reaction from **O′** to **P′**, which we had observed in some substrates having an electron‐rich B‐ring, leading to an S_N_1 ionization,^[10b]^ we protected the *p*‐phenol on the B‐ring using an electron‐withdrawing group (EWG).

**Scheme 10:**
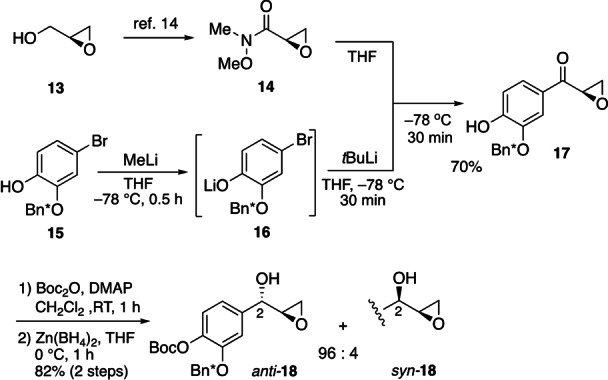
Synthesis of epoxy alcohol **18**. Boc=*tert*‐butoxycarbonyl, DMAP=4‐dimethylaminopyridine.

Following this strategy, we synthesized epoxy alcohol *anti*‐**18** as depicted in Scheme 10. Using (*S*)‐glycidol (**13**)^[13]^ as a substrate, we prepared Weinreb amide **14** (66 % yield in two steps),^[14]^ which turned out to be labile and had to be used immediately for the next step. Meanwhile, to introduce the aryl moiety, we used aryl bromide **15** having a free phenol, which would later be protected using *tert*‐butoxycarbonyl (Boc) as an EWG. Thus, phenol **15** ^[15]^ was treated with MeLi (THF, 0.5 h),^[16]^ and the resulting phenoxide **16** was subjected to bromine‐lithium exchange (*t*BuLi, −78 °C, 0.5 h). Amide **14** (−78 °C, 0.5 h) was added to the resulting aryllithium solution, furnishing ketone **17** in 70 % yield. After protection of the B‐ring phenol in **17** with a Boc group (Boc_2_O, 4‐dimethylaminopyridine, room temperature, 1 h),^[17]^ reduction with Zn(BH_4_)_2_ (THF, 0 °C, 1 h)^[18]^ gave epoxy alcohol **18** with high stereoselectivity (*anti*/*syn=*96 : 4). We successfully separated the *anti‐* **18** and *syn*‐**18** diastereomers by silica gel column chromatography.

**Scheme 11:**
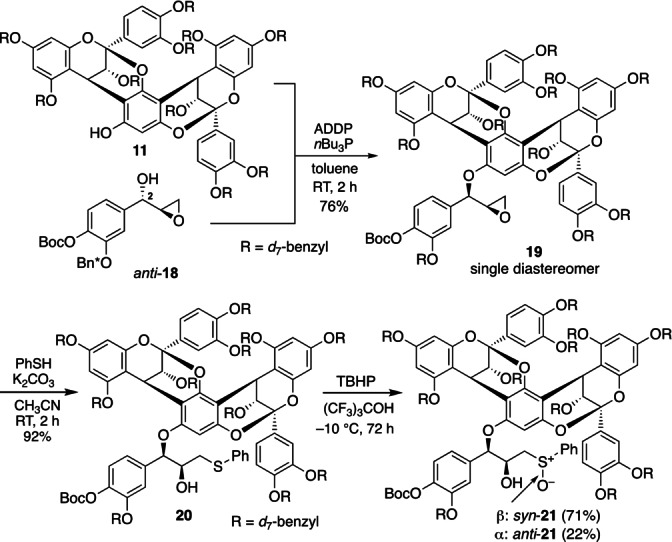
Synthesis of sulfoxide **21**. ADDP= 1,1′‐(azodicarbonyl)dipiperidine, TBHP=*tert‐*butyl hydroperoxide.

Subsequently, we performed the modified Mitsunobu reaction (1,1′‐azodicarbonyl)dipiperidine, *n*Bu_3_P)^[19]^ of epoxy alcohol *anti*‐**18** and phenol **11**, which proceeded with a complete inversion of the stereochemistry to give epoxy ether **19** in 76 % yield (Scheme 11). We then treated epoxide **19** with thiophenol in the presence of K_2_CO_3_, giving hydroxy‐sulfide **20**. Oxidation of **20** [*tert‐*butyl hydroperoxide, (CF_3_)_3_COH, −10 °C, 72 h]^[20]^ gave a separable mixture of *syn*‐ and *anti*‐**21** in 71 % and 22 % yield, respectively.^[21]^

Having sulfoxides **21** in hand, we examined the planned cyclization via Pummerer/Friedel–Crafts cascade^[22]^ (Table 1). However, the reaction of *syn*‐**21** with TMSOTf in the presence of Et_3_N (CH_2_Cl_2_, 0 °C, 0.5 h) gave sulfide **23** in 60 % yield instead of the desired cyclized product **22** (run 1).^[23]^ After an extensive screening of conditions, we found that the use of *i*Pr_2_NEt led to the formation of **22**[^24^, ^25^] in 68 % yield, although a small amount of **23** was still obtained (run 2). Upon further pursuit, we found that an even bulkier base, 1,2,2,6,6‐pentamethylpiperidine, enabled a slow but clean conversion of *syn*‐**21** (TMSOTf, CH_2_Cl_2_, 0 °C, 14 h) into **22** in 76 % yield (run 3) without formation of the reduced product **23**.

**Table 1:** Cyclization of *syn*‐**21** via Pummerer/Friedel–Crafts cascade.

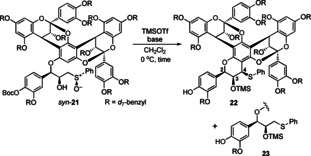				
Run	Base	*t* [h]	**22**: yield/%	**23**: yield/%
1	Et_3_N	0.5	–^[a]^	60
2	*i*Pr_2_NEt	1.5	68	ca. 7
3	*N*‐Me‐TMP^[b]^	14	76	–^[a]^

[a] Not detected. [b] *N*‐Me‐TMP=1,2,2,6,6‐pentamethylpiperidine.

According to the diagnostic NOEs between the hydrogen atoms at C2, C3, and C4 positions, we assigned the relative stereochemistry of **22** as all *syn*.

**Scheme 12:**
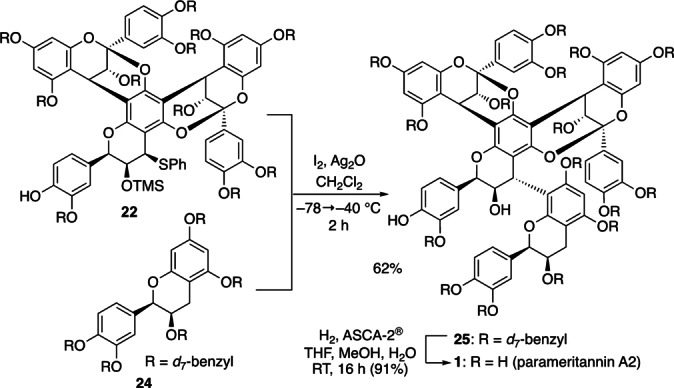
Endgame.

We then tackled the union of the final EC unit (Scheme 12). The activation of sulfide **22** using I_2_ and Ag_2_O (CH_2_Cl_2_, −78 °C→−40 °C, 2 h)[^4^, ^5^] and its union with the bottom EC unit **24** afforded tetramer **25** in 62 % yield. Subsequent detachment of the benzyl groups in **25** [H_2_ (1 atm), ASCA‐2®,^[26]^ MeOH, THF, H_2_O], anaerobic filtration (argon), removal of the volatiles, and lyophilization gave crude **1**. Reverse‐phase preparative HPLC^[27]^ and lyophilization afforded **1** as an ivory amorphous solid (91 % yield), whose physical data were indistinguishable from the reported data of natural **1** {^1^H and ^13^C NMR, IR, high‐resolution MS (ESI),^[1]^ [α]^28^_D_=+13 (*c*=0.50, MeOH) [lit.^[2]^ [α]^28^_D_=+12.1 (*c*=0.5, MeOH)]}.

## Conclusion

In summary, we achieved the first total synthesis of parameritannin A2 (**1**). The key features include (1) a phloroglucinol trick to circumvent the C4−C6 bond‐forming issue and (2) a de novo construction of the flavan skeleton with a leaving group at C4 position, which served for the final assembly of the bottom EC unit to complete the construction of the branched tetrameric structure. The strategies and tactics presented herein will provide flexible synthetic access to various oligomeric catechins with potential biological activities.

## Conflict of interest

The authors declare no conflict of interest.

1

## Supporting information

As a service to our authors and readers, this journal provides supporting information supplied by the authors. Such materials are peer reviewed and may be re‐organized for online delivery, but are not copy‐edited or typeset. Technical support issues arising from supporting information (other than missing files) should be addressed to the authors.

Supporting Information

## Data Availability

The data that support the findings of this study are available in the Supporting Information of this article.
