# Supplementary material for: Total Synthesis of Parameritannin A2, a Branched Epicatechin Tetramer with Two Double Linkages
Source: Angew Chem Int Ed Engl. 2022 May 20;61(28):e202205106. doi: 10.1002/anie.202205106 (PMC9401032; doi:10.1002/anie.202205106)
Supplement: Supplementary file 1 — Supporting Information [file ANIE-61-0-s001.pdf]

Angewandte Chemie
Eine Zeitschrift der Gesellschaft Deutscher Chemiker

Supporting Information

Total Synthesis of Parameritannin A2, a Branched Epicatechin Tetramer with Two Double Linkages

*V. V. Betkekar, K. Suzuki, K. Ohmori**

General

All reactions utilizing air- or moisture-sensitive reagents were performed in flame-dried glasswares under an atmosphere of dry argon. Ethereal solvents and dichloromethane (anhydrous; Kanto Chemical Co., Inc.) were purified under argon, using an Organic Solvent Pure Unit (Wako Pure Chemical Industries, Ltd.). For thin-layer chromatography (TLC) analysis, Merck pre-coated plates (TLC silica gel 60 F254, Art 5715, 0.25 mm) were used. Silica-gel preparative thin-layer chromatography (PTLC) was performed using plates prepared from Merck Silica gel 60 PF254 (Art 7747). For flash column chromatography, silica gel 60N (Spherical, neutral, 63–210 μm) from Kanto Chemical was used. Melting point (mp) determinations were performed by using a METTLER TOLEDO MP70 melting point system and are uncorrected. ^{1}H-, and ^{13}C-NMR were measured on a Bruker Avance III (600 MHz) spectrometer equipped with the cold probe (CryoProbe Prodigy^{TM}) and in the solvent indicated; Chemical shifts (d) are expressed in parts per million (ppm) downfield from internal standard (tetramethylsilane 0.00 ppm) or referenced to residual undeuterated solvents as internal standard. All coupling constants (*J*) are reported as hertz (Hz). Splitting patterns are indicated as follows: s = singlet, d = doublet, t = triplet, q = quartet, quint = quintet, m = multiplet, br = broad. Infrared (IR) spectra were recorded on a Thermo SCIENTIFIC NICOLET iS5 FT-IR spectrometer. Attenuated total reflectance Fourier transform infrared (ATR-FTIR) spectra were recorded by using Thermo SCIENTIFIC NICOLET iS5 FTIR spectrometer equipped iD5 ATR accessory. High-resolution mass spectra (HRMS) were obtained with Bruker Daltonics micrOTOF-QII. Optical rotations ($[\alpha]_D$) were measured on a JASCO P-3000 polarimeter. High performance liquid chromatography (HPLC) analyses were performed on a LC-Net II/ADC controller (JASCO) equipped with a Jasco PU-2080 Plus Intelligent Pump, a Jasco MD-2010 Plus multiwavelength detector, a Jasco DG-2080-54 degasser and LG-2080-02 Ternary Gradient Unit. Preparative HPLC separation was performed on a LC-Net II/ADC controller (JASCO) equipped with a Jasco PU-2086 Plus Intelligent Prep Pump, a Jasco UV-1575 UV/Vis detector and a Jasco DG-2080-54 degasser.

Reaction of dimer 7 and 6

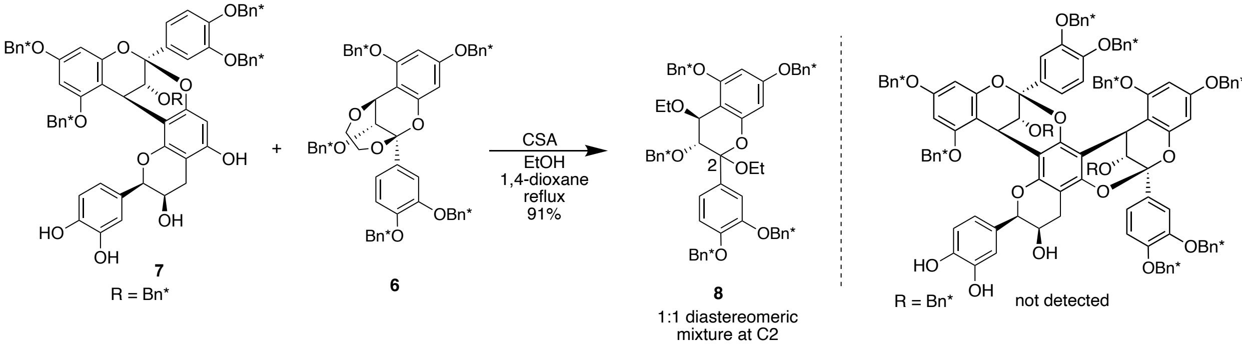

To a mixture of dimer **7**[1] (13 mg 0.012 mmol) and acetal **6** (10 mg, 0.012 mmol) in mixed solvent EtOH and 1,4-dioxane (v/v =1, 0.5 mL) was added camphor sulfonic acid (CSA) (5.5 mg, 0.024 mmol) at room temperature. The reaction mixture was stirred at 70 °C for 90 min. After cooled to room temperature, reaction mixture was quenched sat. aqueous $NaHCO_3$ solution. The crude products were extracted with EtOAc (x3). Combined organic extracts were washed with water, brine, dried (Na_2SO_4) and concentrated in vacuo. The residue was purified by preparative TLC (silica-gel, hexane/EtOAc = 3/2) to afford **8** (9.5 mg, 91%) as a viscous oil. R_f = 0.83 (hexane/EtOAc = 7/3); $[\alpha]_D^{20}$ = –28 (*c* 1.5, $CHCl_3$); ^{1}H NMR (600 MHz, $CDCl_3$) δ 0.96 (t, *J* = 4.8 Hz, 3H), 1.01 (t, *J* = 4.8 Hz, 3H), 3.20–3.23 (m, 1H), 3.35–3.42 (m, 2H), 3.50–3.52 (m, 1H), 3.69 (d, *J* = 1.2 Hz, 1H), 4.37 (d, *J* = 1.2 Hz, 1H), 6.28 (d, *J* = 2.4 Hz, 1H), 6.30 (d, *J* = 2.4 Hz, 1H), 6.96 (d, *J* = 7.8 Hz, 1H), 7.15 (d, *J* = 7.8 Hz, 1 H) 7.16 (s, 1H); ^{13}C NMR (150 MHz, $CDCl_3$) δ 15.4, 15.7, 58.1, 64.5, 69.2–69.8 (m), 70.2–70.9 (m), 70.5, 71.5–72.0 (m), 94.7, 95.3, 101.2, 103.8, 114.4, 120.9, 127.3–128.3 (m), 133.1, 136.8, 137.0, 137.2, 137.3, 148.6, 148.9, 153.0, 159.8, 160.6; IR (neat) 2912, 2848, 1666, 1650, 1643, 1580, 1538, 1503, 1462, 1454, 1159, 1089, 503 cm^{-1}; HRMS (ESI) calcd for $C_{54}H_{18}D_{35}O_8$ $[(M+H)^+]$ *m/z* 864.5931, Found *m/z* 864.5890.

Synthesis of Phenol 11

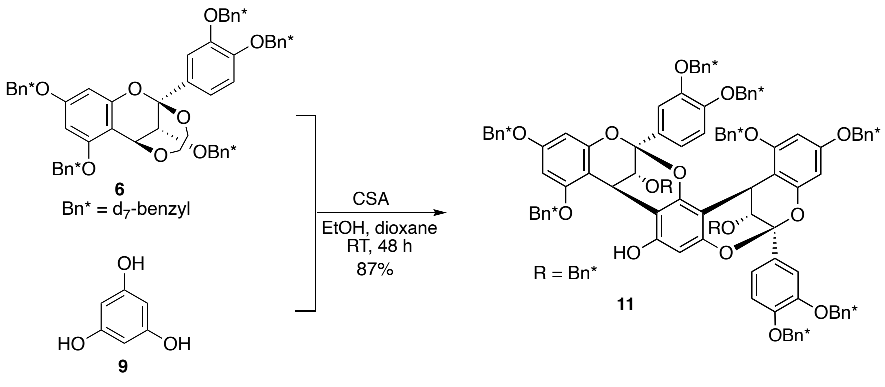

To a solution of acetal **6** (6.4 g, 7.6 mmol) and phloroglucinol (**9**) (0.40 g, 3.2 mmol) in mixed solvent EtOH and 1,4-dioxane (v/v =1, 60 mL) was added camphor sulfonic acid (CSA) (3.3 g, 14 mmol) at room temperature. After stirring for 48 h, it was quenched with sat. aqueous $NaHCO_3$ solution. The crude products were extracted with EtOAc (x3). Combined organic extracts were washed water, brine, dried (Na_2SO_4) and concentrated in vacuo. The residue was purified by flash column chromatography (silica-gel, hexane/EtOAc/CH_2Cl_2 = 7/1/1) to afford phenol **11** (4.6 g, 87%) as an ivory foam. R_f = 0.65 (hexane/EtOAc/CH_2Cl_2 = 4/1/1); $[\alpha]_D^{20}$ = –19 (*c* 0.82, $CHCl_3$); ^{1}H NMR (600 MHz, $CDCl_3$) δ 3.81 (d, *J* = 3.0 Hz, 1H), 3.84 (d, *J* = 3.6 Hz, 1H), 4.37 (d, *J* = 3.6 Hz, 1H), 4.98 (d, *J* = 3.0 Hz, 1H), 5.96 (d, *J* = 1.8 Hz, 1H), 6.15 (s, 1H), 6.24 (d, *J* = 2.4 Hz, 1H), 6.26 (d, *J* = 2.4 Hz, 1H), 6.30 (d, *J* = 2.4 Hz, 1H), 6.91 (s, 1H), 6.95 (d, *J* = 8.4 Hz, 1H), 6.96 (d, *J* = 8.4 Hz, 1H), 7.19–7.22 (m, 2H), 7.25 (d, *J* = 1.8 Hz, 1H), 7.37 (d, *J* = 1.8 Hz, 1H); ^{13}C NMR (150 MHz, $CDCl_3$) δ 25.5, 25.9, 68.7–71.3 (m), 72.4, 72.9, 94.5, 94.9, 95.3, 96.1, 98.2, 98.4, 98.6, 105.0, 105.1, 106.4, 106.7, 114.4, 114.5, 114.5, 115.5, 120.58, 120.61, 127.0–128.8 (m), 132.5, 132.6, 135.0, 135.6, 136.8, 137.1, 137.2, 137.4, 138.1, 148.5, 141.6, 149.4, 149.6, 152.4, 152.8, 153.4, 143.7, 155.4, 157.6, 158.9, 159.1; IR (neat) 3419, 3011, 2359, 2341, 2188, 1617, 1508, 1490, 1418, 1273, 1183, 1140, 1085, 1052, 1036, 960, 819, 751, 541 cm^{-1}; HRMS (ESI) calcd for $C_{106}H_{17}D_{70}O_{15}$ $[(M+H)^+]$ *m/z* 1670.0433, Found *m/z* 1670.0453.

Synthesis of Bromo-phenol 15

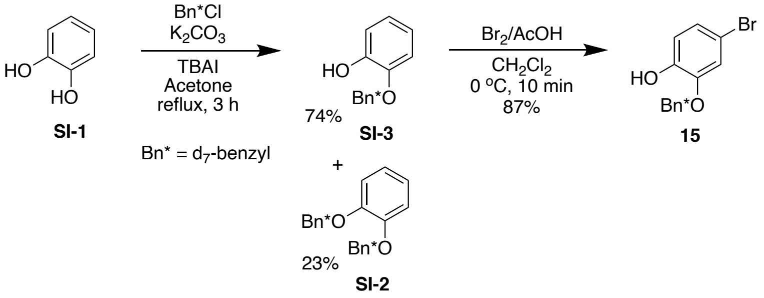

2-(*d_7*-benzyloxy)phenol (SI-3): To a suspension of catechol **SI-1** (5.0 g, 45 mmol) and K_2CO_3 (6.3 g, 45 mmol) in acetone (65 mL), was added *d_7*-benzyl chloride (7.3 mL, 64 mmol) and tetrabutylammonium iodide (1.7 g, 4.5 mmol). The resulting mixture was stirred at reflux for 3 h. After cooled to room temperature, Et_2NH (5 mL) was added and stirred for additional 1 h. Reaction mixture was diluted with diethyl ether and filtered through pad of Celite® and residue was washed with diethyl ether. The combined filtrate was washed with water, brine, dried (Na_2SO_4) and concentrated in vacuo. The residue was purified by flash column chromatography (silica-gel, hexane/EtOAc = 6/1) to afford phenol **SI-2** (3.2 g, 23%) as a white solid and **SI-3** (7.0 g, 74%) as a colorless oil, respectively.

SI-2: R_f = 0.65 (hexane/EtOAc = 3/1); mp: 60.9–61.2 °C; ^{1}H NMR (600 MHz, $CDCl_3$) δ 6.88–6.90 (m, 2H), 6.94–6.97 (m, 2H); ^{13}C NMR (150 MHz, $CDCl_3$) δ 70.6 (quint, $J_{C\text{-}d}$ = 22.5 Hz), 115.2, 121.7, 127.0 (t, $J_{C\text{-}d}$

= 24.0 Hz), 127.4 (t, $J_{C\text{-}d}$ = 24.0 Hz), 128.1 (t, $J_{C\text{-}d}$ = 24.0 Hz), 137.2, 149.0; IR (neat) 2209, 1589, 1496, 1324, 1290, 1217, 1197, 1057, 1037, 976, 872, 738, 729 cm^{-1}; HRMS (ESI) calcd for $C_{20}H_4D_{14}O_2Na$ $[(M+Na)^+]$ *m/z* 327.2078, Found *m/z* 327.2079.

SI-3: R_f = 0.60 (hexane/EtOAc = 3/1); ^1H NMR (600 MHz, $CDCl_3$) δ 5.79 (s, 1H), 6.89 (dt, *J* = 1.2, 7.8 Hz, 1H), 6.94 (dt, *J* = 1.2, 7.8 Hz, 1H), 6.97 (dd, *J* = 1.2, 7.8 Hz, 1H), 7.01 (dd, *J* = 1.2, 7.8 Hz, 1H); ^{13}C NMR (150 MHz, $CDCl_3$) δ 70.4 (quint, $J_{C\text{-}d}$ = 22.5 Hz), 112.3, 114.9, 120.3, 121.9, 127.6 (t, $J_{C\text{-}d}$ = 24.0 Hz), 127.8 (t, $J_{C\text{-}d}$ = 24.0 Hz), 128.3 (t, $J_{C\text{-}d}$ = 24.0 Hz), 136.2, 145.9, 150.0; IR (neat) 3063, 2580, 2210, 1589, 1496, 1254, 1125, 1057, 1037, 823, 738, 729, 666 cm^{-1}; HRMS (ESI) calcd for $C_{13}H_5D_7O_2Na$ $[(M+Na)^+]$ *m/z* 230.1169, Found *m/z* 230.1168.

2-(d_7-benzyloxy)-4-bromophenol (15):[2] To a solution of 2-(d_7-benzyloxy)phenol **SI-3** (6.0 g, 29 mmol) in CH_2Cl_2/AcOH (50 mL, v/v = 2/1) at 0 °C was added a solution of Br_2 (1.5 mL, 29 mmol) in AcOH (15 mL) dropwise over a period of 10 min and stirred for 10 min. Reaction mixture was quenched by addition of sat. aqueous Na_2SO_3 solution. The crude products were extracted with CH_2Cl_2 (x3). Combined organic extracts was successively washed with sat. aqueous Na_2SO_3 solution, water, brine, dried (Na_2SO_4) and concentrated in vacuo. The crude product was purified by flash column chromatography (silica-gel, hexane/EtOAc = 3/1) to afford phenol **15** (7.2 g, 87%) as a pale-yellow solid. R_f = 0.75 (hexane/EtOAc = 2/1); mp: 66–68 °C; ^1H NMR (600 MHz, $CDCl_3$) δ 5.61 (s, 1H), 6.83 (d, *J* = 8.4 Hz, 1H), 7.02 (dd, *J* = 1.8, 8.4, 1H), 7.07 (d, *J* = 1.8 Hz, 1H); ^{13}C NMR (150 MHz, $CDCl_3$) δ 70.8 (quint, $J_{C\text{-}d}$ = 22.5 Hz), 111.6, 115.7, 116.1, 124.7, 127.4 (t, $J_{C\text{-}d}$ = 24.0 Hz), 128.2–128.7 (m), 135.5, 145.3, 146.6; IR (neat) 3521, 1605, 1586, 1493, 1417, 1358, 1329, 1200, 1114, 1050, 1037, 982, 839, 808, 544 cm^{-1}; HRMS (ESI) calcd for $C_{13}H_4D_7O_2BrNa$ $[(M+Na)^+]$ *m/z* 308.0274, Found *m/z* 308.0262.

Synthesis of Weinreb amide 14

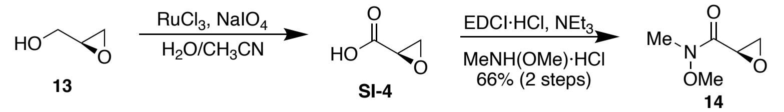

(*R*)-*N*-Methoxy-*N*-methyloxirane-2-carboxamide (14):[3] To the solution of (+)-(*S*)-glycidol **13** (3.0 g, 41 mmol) in CH_3CN (70 mL) was added H_2O (1.90 mL, 105 mmol) and $NaIO_4$ (26.0 g, 121 mmol), followed by addition of $RuCl_3$ (84 mg, 0.41 mmol). The stirring of the reaction mixture at room temperature for 3 h gave a green suspension, and an additional portion of H_2O (1.9 mL, 105 mmol) was added to the reaction mixture at 0 °C. The stirring was continued for 4 h at 0 °C and 8 h at room temperature. The reaction mixture was diluted with Et_2O, and the resultant suspension was filtered through a pad of silica-gel (ca. 20 mm thick, eluted with Et_2O). The filtrate was concentrated in vacuo and the residue was again dissolved in Et_2O. The Et_2O solution was again filtered through a pad of silica gel (ca. 20 mm thick, eluted with Et_2O). The solvent

was removed under vacuo to afford the crude product of (*R*)-glycidic acid **SI-4** (3.4 g) as a yellow oil, which was used immediately in the next step without further purification.

To the solution of the crude acid (*R*)-glycidic acid **SI-4** (3.4 g, ca. 39 mmol) in CH_2Cl_2 (80 mL) was added NEt_3 (60 mL, 43 mmol), DMAP (5 mg, 0.04 mmol) and *N,O*-dimethyl-hydroxylamine hydrochloride (4.8 g, 49 mmol) at 0 °C. The resultant mixture was stirred for 10 min at −5 °C and 1-(3-dimethylaminopropyl)-3-ethylcarbodiimide hydrochloride (EDCI·HCl, 9.9 g, 52 mmol) was added. The reaction mixture was stirred for 4 h at 0 °C and diluted with hexane. The mixture was filtered through a pad of silica gel (2 cm thick, eluted with Et_2O). The solvent was removed under reduced pressure to obtain the crude amide **14** (3.5 g, 66% from **13**) as yellow oil. This crude product was dissolved in CH_2Cl_2 (10 mL) and dried over molecular sieves 4A (beads) at −20 °C for 2 h before used in the next step.

Synthesis of epoxy-alcohol *anti*-18

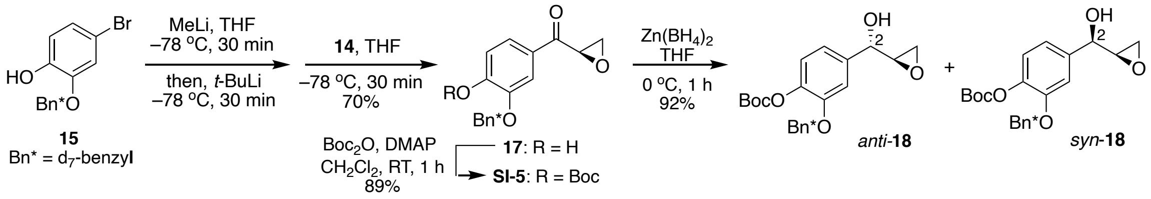

Phenol 17: To the solution of bromophenol **15** (4.0 g, 14 mmol) in THF (60 mL) at –78 °C, was added MeLi in Et_2O (1.2 M, 13 mL, 16 mmol) and stirred at same temperature. After 30 min, a solution of *tert*-butyllithium in pentane (1.6 M, 19 mL, 30 mmol) was added to the reaction mixture and stirred for additional 30 min. To this, a solution of crude **14** (2.7 g, ca. 21 mmol) in THF (10 mL) was added dropwise at –78 °C and stirred for additional 30 min. The reaction was carefully quenched by addition of sat. aqueous NH_4Cl solution. The crude products were extracted with EtOAc (x3). Combined organic extracts were washed with water, brine, dried (Na_2SO_4) and the solvent removed in vacuo. The crude product was purified by flash column chromatography (silica-gel, hexane/EtOAc = 4/3) to afford phenol **17** (2.7 g, 70%) as a white solid. R_f = 0.55 (hexane/EtOAc = 2/1); mp: 100–101 °C; $[\alpha]_D^{20}$ = +15 (*c* 0.85, $CHCl_3$); ^{1}H NMR (600 MHz, $CDCl_3$) δ 2.93 (dd, J = 2.4, 6.6 Hz, 1H), 3.08 (dd, J = 4.2, 6.6 Hz, 1H), 4.19 (dd, J = 2.4, 4.2 Hz, 1H), 6.40 (brs, 1H), 7.00 (d, J = 8.4 Hz, 1H), 7.66 (d, J = 1.8 Hz, 1H), 7.70 (dd, J = 1.8, 8.4 Hz, 1H); ^{13}C NMR (150 MHz, $CDCl_3$) δ 47.7, 50.9, 70.6 (quint, $J_{C\text{-}d}$ = 22.5 Hz), 111.4, 114.5, 124.3, 127.8 (t, $J_{C\text{-}d}$ = 24.0 Hz), 128.2–128.6 (m), 135.3, 146.2, 151.6, 192.9; IR (neat) 3375, 1673, 1590, 1513, 1439, 1385, 1290, 1189, 1052, 896, 734, 625, 546 cm^{-1}; HRMS (ESI) calcd for $C_{16}H_7D_7O_4Na$ [(M+Na)$^+$] *m/z* 300.1224, Found *m/z* 300.1228.

Ketone (SI-5): To the solution of phenol **17** (2.0 g, 7.2 mmol) in CH_2Cl_2 (25 mL) at 0 °C, was added DMAP (0.44 g, 3.6 mmol) and Boc_2O (1.7 g, 7.9 mmol). It was warmed to room temperature and stirred for additional 1 h. The reaction was quenched by addition of aqueous 1 M HCl and the crude products were extracted with EtOAc (x3). Combined organic extracts were washed with water, brine, dried (Na_2SO_4) and the solvent removed in vacuo. The crude product was purified by flash column chromatography (silica-gel, hexane/EtOAc = 1/1) to afford **SI-5** (2.4 g, 89%) as a white solid. R_f = 0.65 (hexane/EtOAc = 2/1); mp: 129.6–130.1 °C; $[\alpha]_D^{20}$ = +14 (*c* 0.90, $CHCl_3$); ^1H NMR (600 MHz, $CDCl_3$) δ 1.47 (s, 9H), 2.94 (dd, *J* = 2.4, 6.0 Hz, 1H), 3.10 (dd, *J* = 4.2, 6.0 Hz, 1H), 4.16 (dd, *J* = 2.4, 4.2 Hz, 1H), 7.25–7.27 (m, 1H), 7.70–7.71 (m, 2H); ^{13}C NMR (150 MHz, $CDCl_3$) δ 27.6, 47.7, 51.3, 70.2 (quint, $J_{C\text{-}d}$ = 22.5 Hz), 84.2, 113.1, 122.2, 123.0, 127.4 (t, $J_{C\text{-}d}$ = 24.0 Hz), 127.8 (t, $J_{C\text{-}d}$ = 24.0 Hz), 128.3 (t, $J_{C\text{-}d}$ = 24.0 Hz), 134.0, 135.6, 145.1, 150.6, 151.1, 193.6; IR (neat) 2982, 1761, 1703, 1599, 1480, 1395, 1256, 1203, 1256, 1203, 1184, 1147, 1125, 1084, 1052, 889, 780, 734, 636, 546 cm^{-1}; HRMS (ESI) calcd for $C_{21}H_{15}D_7O_6Na$ $[(M+Na)^+]$ *m/z* 400.1748, Found *m/z* 400.1763.

Epoxy alcohol *anti*-18: To the solution of ketone **SI-5** (2.00 g, 5.29 mmol) in THF (40 mL) at 0 °C, was added $Zn(BH_4)_2$ solution[4] (0.66 M in THF, 18 mL, 11.8 mmol) dropwise. After stirring for 1 h, the reaction was carefully quenched by adding MeOH/THF (v/v = 1/9) followed by water. The crude products were extracted with EtOAc (x3). Combined organic extracts were washed with water, brine, dried (Na_2SO_4) and the solvent removed in vacuo. The crude product was purified by silica-gel flash column chromatography (hexane/EtOAc = 1/1) to afford epoxy alcohol **18** (1.84 g, 92%) as a diastereomeric mixture (96:4). The minor diastereomer, i.e., *syn*-**18**, was removed by column chromatography (silica-gel, hexane/acetone = 3/1) to afford *anti*-**18** (1.78 g) as a single diastereomer. R_f = 0.35 (hexane/EtOAc = 2/1); mp: 87–89 °C; $[\alpha]_D^{20}$ = +36 (*c* 1.6, $CHCl_3$); ^1H NMR (600 MHz, $CDCl_3$) δ 1.47 (s, 9H), 2.52 (d, *J* = 1.8 Hz, 1H), 2.68 (t, *J* = 4.8 Hz, 1H), 2.84 (dd, *J* = 3.0, 4.8 Hz, 1H), 3.14 (m, 1H), 4.82 (brm, 1H), 6.95 (dd, *J* = 1.8, 8.4 Hz, 1H), 7.06 (d, *J* = 1.8 Hz, 1H), 7.13 (d, *J* = 8.4 Hz, 1H); ^{13}C NMR (150 MHz, $CDCl_3$) δ 27.7, 43.6, 55.1, 70.1 (quint, $J_{C\text{-}d}$ = 22.5 Hz), 70.6, 83.5, 112.0, 118.9, 122.7, 127.2 (t, $J_{C\text{-}d}$ = 24.0 Hz), 127.6–128.2 (m), 136.3, 138.5, 140.3, 150.7, 151.5; IR (neat) 3475, 2981, 2932, 1759, 1606, 1509, 1474, 1429, 1395, 1230, 1083, 1051, 911, 889, 841, 815, 733, 546 cm^{-1}; HRMS (ESI) calcd for $C_{21}H_{17}D_7O_6Na$ $[(M+Na)^+]$ *m/z* 402.1905, Found *m/z* 402.1910.

Synthesis of sulfide 20

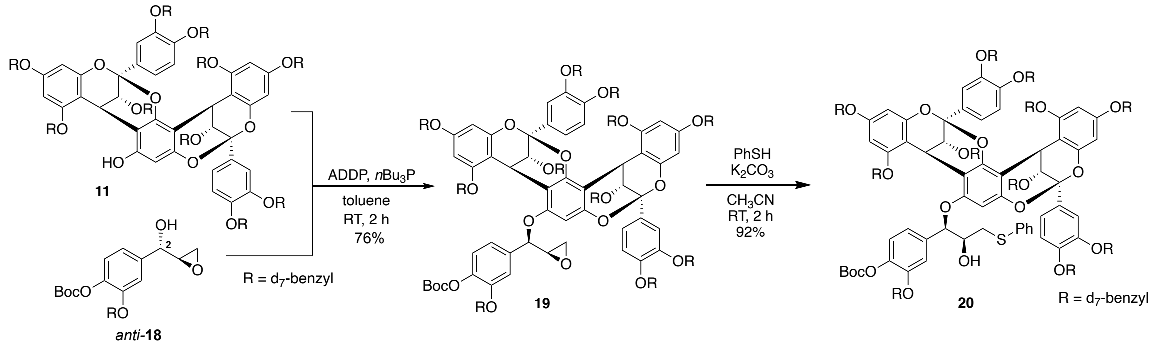

Epoxy ether 19: To a solution of **11** (2.00 g, 1.19 mmol), epoxide *anti*-**18** (0.68 g, 1.8 mmol) and 1,1'-(azodicarbonyl)dipiperidine (ADDP) (0.91 g, 3.6 mmol) in toluene (35 mL) was added *n*-Bu_3P (0.90 mL, 3.6 mmol) at 0 ºC. The reaction mixture was warmed to room temperature and stirred for 2 h. The reaction was quenched by addition of phosphate buffer (pH = 7) solution. The crude products were extracted with EtOAc (x3). Combined organic extracts were washed with water, brine, dried (Na_2SO_4) and the solvent was removed in vacuo. The crude product was purified by flash column chromatography (silica-gel, hexane/EtOAc/CH_2Cl_2= 5/1/1) to afford **19** (1.86 g, 76%) as an ivory foam. R_f = 0.65 (hexane/EtOAc/CH_2Cl_2 = 4/1/1); $[\alpha]_D^{20}$ = –32 (*c* 0.47, $CHCl_3$); ^{1}H NMR (600 MHz, $CDCl_3$) δ 1.47 (s, 9H), 2.41 (dd, *J* = 2.4, 4.8 Hz, 1H), 2.50 (t, *J* = 4.8 Hz, 1H), 2.97 (ddd, *J* = 2.4, 4.8, 6.0 Hz, 1H), 3.80 (d, *J* = 3.0 Hz, 1H), 3.81 (d, *J* = 3.0 Hz, 1H), 4.67 (d, *J* = 6.0 Hz, 1H), 5.00 (d, *J* = 3.0 Hz, 1H), 5.14 (d, *J* = 3.0 Hz, 1H), 6.00 (d, *J* = 2.4 Hz, 1H), 6.07 (s, 1H), 6.22 (d, *J* = 2.4 Hz, 1H), 6.25 (d, *J* = 2.4 Hz, 1H), 6.26 (d, *J* = 2.4 Hz, 1H), 6.91 (dd, *J* = 1.8, 8.4 Hz, 1H), 6.94 (d, *J* = 7.8 Hz, 1H), 6.95 (d, *J* = 8.4 Hz, 1H), 6.99 (d, *J* = 1.8 Hz, 1H), 7.05 (d, *J* = 8.4 Hz, 1H), 7.11 (dd, *J* = 1.8, 7.8 Hz, 1H), 7.17 (dd, *J* = 1.8, 8.4 Hz, 1H), 7.27 (d, *J* = 1.8 Hz, 1H), 7.38 (d, *J* = 1.8 Hz, 1H); ^{13}C NMR (150 MHz, $CDCl_3$) δ 25.3, 26.1, 27.7, 44.9, 54.6, 69.1–71.2 (m), 72.6, 73.0, 82.0, 83.6, 94.5, 94.8, 94.9, 95.5, 104.9, 105.2, 106.9, 108.5, 104.9, 105.2, 106.9, 108.5, 112.4, 114.46, 114.50, 115.1, 119.1, 120.3, 123.0, 127.1–128.2 (m), 132.4, 133.0, 136.0, 136.5, 136.8, 136.9, 137.1, 137.2, 137.4, 137.8, 138.1, 140.5, 148.5, 148.6, 149.3, 149.6, 150.8, 151.5, 151.9, 153.3, 154.0, 154.1, 157.6, 157.8, 158.8, 159.0; IR (neat) 2980, 2119, 1759, 1508, 1490, 1419, 1327, 1183, 1145, 1086, 1052, 1038, 869, 839, 818, 736, 543 cm^{-1}; HRMS (ESI) calcd for $C_{127}H_{31}D_{77}O_{20}Na$ $[(M+Na)^+]$ *m/z* 2053.2160, Found *m/z* 2053.2182.

Sulfide 20: To a solution of **19** (1.80 g, 0.886 mmol), and K_2CO_3 (0.61 g, 4.4 mmol) in CH_3CN (30 mL) was added PhSH (0.45 mL, 4.4 mmol) at room temperature. It was stirred for 2 h at room temperature and diluted with diethyl ether. Reaction mixture was filtered through a Celite® pad and washed with diethyl ether (x3). The combined filtrate was concentrated in vacuo and purified by flash column chromatography

(silica-gel, hexane/EtOAc/CH_2Cl_2= 5/1/1) to afford **20** (1.75 g, 92%) as an ivory foam. R_f = 0.80 (hexane/EtOAc/CH_2Cl_2 = 3/1/1); $[\alpha]_D^{20}$ = –67 (*c* 0.38, $CHCl_3$); ^{1}H NMR (600 MHz, $CDCl_3$) δ 1.45 (s, 9H), 2.65 (dd, *J* = 6.0, 13.2 Hz, 1H), 2.88 (dd, *J* = 4.2, 13.2 Hz, 1H), 3.52–3.52 (m, 1H), 3.75 (d, *J* = 3.6 Hz, 1H), 3.83 (d, *J* = 4.2 Hz, 1H), 4.32 (d, *J* = 4.2 Hz, 1H), 4.91 (d, *J* = 4.2 Hz, 1H), 4.93 (d, *J* = 3.6 Hz, 1H), 5.01 (d, *J* = 7.2 Hz, 1H), 5.99 (d, *J* = 2.4 Hz, 1H), 6.00 (s, 1H), 6.24 (d, *J* = 2.4 Hz, 1H), 6.34 (d, *J* = 2.4 Hz, 1H), 6.42 (d, *J* = 2.4 Hz, 1H), 6.78 (dd, *J* = 1.8, 8.4 Hz, 1H), 6.88 (d, *J* = 1.8 Hz, 1H), 6.90 (d, *J* = 8.4 Hz, 1H), 6.94 (d, *J* = 8.4 Hz, 2H), 7.01–7.06 (m, 4H), 7.10–7.13 (m, 2H), 7.16 (dd, *J* = 1.8, 8.4 Hz, 1H), 7.22 (d, *J* = 1.8 Hz, 1H), 7.29 (d, *J* = 2.4 Hz, 1H); ^{13}C NMR (150 MHz, $CDCl_3$) δ 25.5, 26.8, 27.8, 29.9, 35.7, 68.7–71.2 (m), 72.7, 72.9, 73.6, 81.3, 83.5, 94.5, 94.8, 95.4, 96.8, 97.5, 98.68, 98.73, 105.1, 106.8, 106.85, 106.90, 112.8, 114.4, 114.46, 114.5, 115.0, 119.5, 120.2, 120.6, 123.0, 125.8, 127.0–128.0 (m), 132.3, 132.5, 135.9, 136.4, 136.6, 136.66, 136.7, 136.8, 137.0, 137.1, 137.2, 137.4, 138.1, 140.5, 148.5, 148.6, 149.1, 149.3, 149.4, 150.9, 151.4, 152.0, 153.4, 153.8, 154.6, 157.2, 157.6, 158.9, 159.0; IR (neat) 3480, 2976, 2118, 1758, 1595, 1506, 1489, 1417, 1327, 1272, 1255, 1181, 1140, 1083, 1050, 1033, 837, 817, 739, 631 cm^{-1}; HRMS (ESI) calcd for $C_{133}H_{37}D_{77}O_{20}SNa$ $[(M+Na)^+]$ *m/z* 2163.2350, Found *m/z* 2163.2323.

Synthesis of *syn*-21

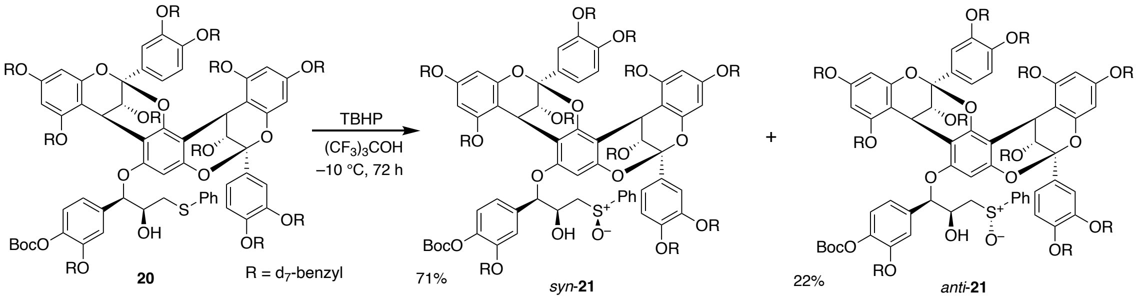

***Syn*-21**: To a solution of **20** (100 mg, 0.047 mmol), in $(CF_3)_3COH$ (3.0 mL) was added TBHP (ca. 5 M in *n*-decane, 0.14 mL, ca. 0.70 mmol) at –10 °C. After stirring for 72 h, the reaction was quenched by adding sat. aqueous Na_2SO_3 solution. The crude products were extracted with EtOAc (x3). Combined organic extracts were washed with water, brine, dried (Na_2SO_4) and concentrated in vacuo. The crude product was purified by preparative TLC (silica-gel, hexane/EtOAc/CH_2Cl_2= 3/1/1, developed three times) to afford sulfoxide *syn*-**21** (72 mg, 71%, more-polar) and *anti*-**21** (22 mg, 22%, less-polar) as an ivory foam, respectively.

Note: The relative stereochemistry of the major (more-polar) and the minor (less-polar) diastereomers of sulfoxide **21** were assigned by the method described by García Ruano.[5,6] The method relies on observation of coupling constant values of methylene protons α to the sulfoxide with the adjacent proton attached to

carbon bearing hydroxy group. It is documented that, *anti*-diastereomers of β-hydroxy sulfoxides display larger $J_{1,2}$ and lower $J_{1,3}$ values compared to those of *syn*-diastereomers. Accordingly, ^{1}H NMR recorded in $CDCl_3$ displayed larger $J_{1,2}$ coupling constant for the less-polar diastereomer of **21** (10.8 Hz) in comparison to that of the more-polar diastereomer (5.4 Hz) (Fig. 1). Along with this, lower values of $J_{1,3}$ for the less-polar diastereomer of **21** (1.8 Hz) was observed in comparison to that of more-polar diastereomer of **21** (4.8 Hz). Thus, with respect to hydroxy group, stereochemistry of sulfoxide was assigned as *syn* in the more-polar (major) diastereomer and *anti in* the less-polar (minor) diastereomer.

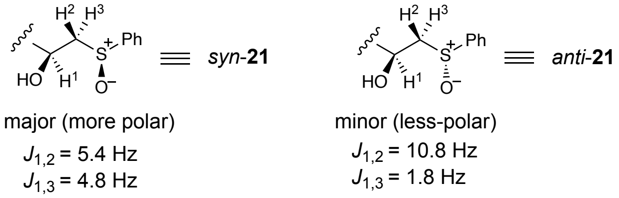

Figure 1: ^{1}H NMR in $CDCl_3$ (600 MHz)

syn-**21**: R_f = 0.25 (hexane/EtOAc/CH_2Cl_2 = 3/1/1); $[\alpha]_D^{20}$ = –75 (*c* 0.62, $CHCl_3$); ^{1}H NMR (600 MHz, $CDCl_3$) δ 1.44 (s, 9H), 2.52 (dd, *J* = 5.4, 13.8 Hz, 1H), 2.78 (dd, *J* = 4.8, 13.8 Hz, 1H), 3.65–3.68 (m, 2H), 3.75 (d, *J* = 3.6 Hz, 1H), 3.83 (d, *J* = 3.6 Hz, 1H), 4.52 (d, *J* = 1.8 Hz, 1H), 4.93 (d, *J* = 3.6 Hz, 1H), 4.94 (d, *J* = 3.6 Hz, 1H), 5.40 (d, *J* = 7.8 Hz, 1H), 5.98 (d, *J* = 2.4 Hz, 1H), 6.14 (s, 1H), 6.23 (d, *J* = 2.4 Hz, 1H), 6.31 (d, *J* = 2.4 Hz, 1H), 6.41 (d, *J* = 2.4 Hz, 1H), 6.82 (dd, *J* = 1.8, 8.4 Hz, 1H), 6.89 (d, *J* = 8.4 Hz, 1H), 6.94 (d, *J* = 8.4 Hz, 1H), 6.98 (d, *J* = 8.4 Hz, 1H), 7.04 (dd, *J* = 1.8, 8.4 Hz, 1H), 7.16 (dd, *J* = 1.8, 8.4 Hz, 1H), 7.19 (d, *J* = 1.8 Hz, 1H), 7.23 (d, *J* = 1.8 Hz, 1H), 7.29 (d, *J* = 1.8 Hz, 1H), 7.40–7.41 (m, 3H), 7.48–7.50 (m, 2H); ^{13}C NMR (150 MHz, $CDCl_3$) δ 25.5, 26.8, 27.7, 59.6, 68.7–70.9 (m), 71.2, 72.7, 72.9, 81.0, 83.5, 94.5, 95.1, 95.4, 96.9, 97.6, 98.5, 114.2, 114.4, 114.5, 114.9, 119.8, 120.2, 120.5, 123.0, 124.3, 127.0–128.5 (m), 129.4, 131.1, 132.3, 132.6, 136.0, 136.1, 136.6, 136.7, 136.8, 137.0, 137.1, 137.2, 137.3, 137.4, 138.1, 140.4, 148.50, 148.54, 149.2, 149.3, 149.4, 150.8, 151.4, 152.1, 153.4, 153.6, 154.5, 157.1, 157.5, 158.9, 159.0; IR (neat) 3483, 1760, 1615, 1508, 1419, 1273, 1182, 1144, 1086, 1052, 1036, 818, 530 cm^{-1}; HRMS (ESI) calcd for $C_{133}H_{37}D_{77}O_{21}SNa$ [$(M+Na)^+$] *m/z* 2179.2299, Found *m/z* 2179.2303.

anti-**21**: R_f = 0.27 (hexane/EtOAc/CH_2Cl_2 = 3/1/1); $[\alpha]_D^{20}$ = –34 (*c* 0.34, $CHCl_3$); ^{1}H NMR (600 MHz, $CDCl_3$) δ 1.44 (s, 9H), 2.48 (dd, *J* = 1.8, 13.2 Hz, 1H), 2.67 (dd, *J* = 10.8, 13.2 Hz, 1H), 3.74 (d, *J* = 3.6 Hz, 1H), 3.81 (d, *J* = 4.2 Hz, 1H), 4.65–4.66 (brm, 1H), 4.79 (d, *J* = 7.8 Hz, 1H), 4.91 (d, *J* = 4.2 Hz, 1H), 4.93 (d, *J* = 3.6 Hz, 1H), 5.96 (s, 1H), 6.00 (d, *J* = 1.8 Hz, 1H), 6.24 (d, *J* = 1.8 Hz, 1H), 6.35 (d, *J* = 1.8 Hz, 1H), 6.45 (d, *J* = 1.8 Hz, 1H), 6.82 (dd, *J* = 1.8, 7.8 Hz, 1H), 6.88 (d, *J* = 1.8 Hz, 1H), 6.90 (d, *J* = 9.0 Hz, 1H), 6.93 (d, *J* = 8.4 Hz, 1H), 7.01 (d, *J* = 8.4 Hz, 1H), 7.05 (dd, *J* = 1.8, 8.4 Hz, 1H), 7.15 (dd, *J* = 1.8, 9.0 Hz, 1H), 7.22 (d, *J* = 2.4 Hz, 1H), 7.27–7.30 (m, 2H), 7.36–7.39 (m, 2H), 7.47–7.49 (m, 2H); ^{13}C NMR (150

MHz, $CDCl_3$) δ 25.4, 26.6, 27.7, 60.5, 69.2, 69.4–71.1 (m), 72.67, 72.71, 82.5, 83.6, 94.47, 94.5, 95.3, 96.9, 97.7, 98.67, 98.71, 105.0, 106.9, 107.0, 107.1, 112.7, 114.40, 114.42, 114.44, 114.9, 119.9, 120.2, 120.5, 123.1, 124.0, 126.3–128.7 (m), 129.4, 131.1, 132.2, 132.5, 135.6, 135.9, 136.3, 136.6, 136.8, 137.0, 137.1, 137.3, 138.0, 140.8, 144.2, 148.49, 148.54, 149.2, 149.3, 149.4, 151.0, 151.2, 152.0, 153.4, 153.7, 154.6, 157.0, 157.6, 158.89, 158.94; IR (neat) 3600, 2915, 2847, 1761, 1617, 1508, 1419, 1273, 1144, 1086, 746, 527 cm^{-1}; HRMS (ESI) calcd for $C_{133}H_{37}D_{77}O_{21}SNa$ [$(M+Na)^+$] *m/z* 2179.2299, Found *m/z* 2179.2298.

Attempt on the cyclization of *syn*-21 using Et_3N as a base

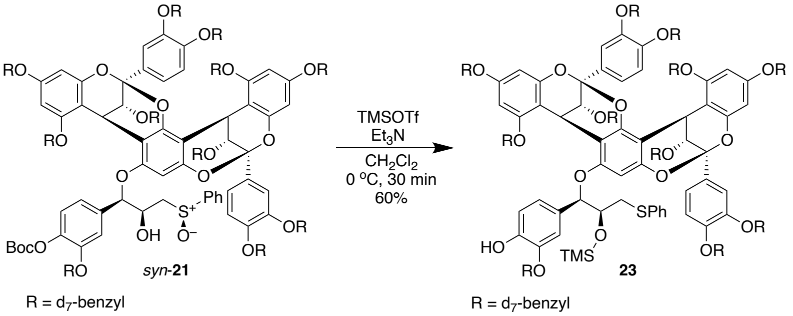

To the solution of *syn*-**21** (50.0 mg, 0.0231 mmol) in CH_2Cl_2 (1.5 mL) at 0 °C, was added Et_3N (49 μL, 0.35 mmol) followed by TMSOTf (55 μL, 0.30 mmol). After stirring for 30 min at 0 °C, reaction was quenched by adding sat. aqueous $NaHCO_3$ solution. The crude products were extracted with EtOAc (x3). The combined organic extracts were washed with water, brine, dried (Na_2SO_4) and concentrated in vacuo. The residue was purified by preparative TLC (silica-gel, hexane/EtOAc/CH_2Cl_2 = 4/1/1) to afford **23** (29.4 mg, 60%) as an ivory foam.

Attempt on the cyclization reaction of *syn*-21 using Hünig's base

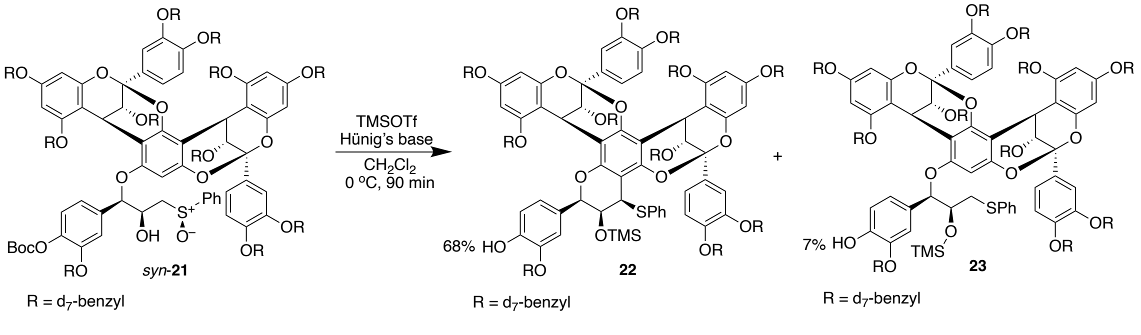

To the solution of *syn*-**21** (50.0 mg, 0.0231 mmol) in CH_2Cl_2 (1.5 mL) at 0 °C was added Hünig's base (61 μL, 35 mmol) followed by TMSOTf (55 μL, 0.30 mmol). After stirring for 90 min at 0 °C, the reaction was quenched by adding sat. aqueous $NaHCO_3$ solution. The crude products were extracted with EtOAc (x3). The combined organic extracts were washed with water, brine, dried (Na_2SO_4) and concentrated in vacuo.

The residue was purified by preparative TLC (silica-gel, hexane/EtOAc/CH_2Cl_2 = 4/1/1) to afford **22** (33.3 mg, 68%) as an ivory foam and **23** (3.4 mg, 7%) as an ivory foam, respectively.

Cyclization reaction of *syn*-21 using *N*-Me-TMP as a base

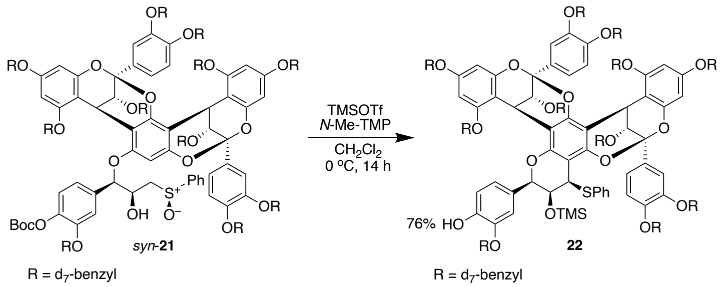

To a solution of *syn*-**21** (50 mg, 0.023 mmol) in CH_2Cl_2 (1.5 mL) at 0 °C was added 1,2,2,6,6-pentamethylpiperidine (*N*-Me-TMP, 64 μL, 0.35 mmol) and TMSOTf (55 μL, 0.30 mmol), and the mixture was stirred at 0 °C. After 14 h, the reaction was stopped by adding sat. aqueous $NaHCO_3$ solution and the products were extracted with EtOAc (x3). The combined organic extracts were washed with water, brine, dried (Na_2SO_4), and concentrated in vacuo. The residue was purified by preparative TLC (silica-gel, hexane, EtOAc, CH_2Cl_2 = 4/1/1) to afford **22** (37 mg, 76%) as an ivory foam.

22: R_f = 0.55 (hexane/EtOAc/CH_2Cl_2 = 4/1/1); $[\alpha]_D^{20}$ = –1.3x10^2 (*c* 0.84, $CHCl_3$); ^{1}H NMR (600 MHz, benzene-d_6) δ 0.20 (s, 9H), 4.19 (d, *J* = 3.0 Hz, 1H), 4.29 (d, *J* = 3.6 Hz, 1H), 4.60 (d, *J* = 5.4 Hz, 1H), 4.73 (t, *J* = 5.4 Hz, 1H), 5.09 (d, *J* = 5.4 Hz, 1H), 5.49 (s, 1H), 5.68 (d, *J* = 3.0 Hz, 1H), 5.74 (d, *J* = 3.6 Hz, 1H), 6.20 (d, *J* = 1.8 Hz, 1H), 6.31 (d, *J* = 1.8 Hz, 1H), 6.38 (d, *J* = 1.8 Hz, 1H), 6.55 (d, *J* = 1.8 Hz, 1H), 6.74–6.75 (m, 3H), 6.87–6.92 (m, 4H), 7.01 (d, *J* = 8.4 Hz, 1H), 7.45 (dd, *J* = 2.4, 8.4 Hz, 1H), 7.56–7.57 (m, 2H), 7.62 (d, *J* = 1.8 Hz, 1H), 7.77 (dd, *J* = 1.8, 8.4 Hz, 1H), 7.89 (d, *J* = 1.8 Hz, 1H); ^{13}C NMR (150 MHz, benzene-d_6) δ 0.3, 26.2, 26.3, 49.6, 68.7–71.2 (m), 70.8, 73.6, 73.9, 77.7, 94.6, 94.99, 95.02, 96.0, 99.5, 99.6, 105.1, 105.4, 105.6, 106.7, 107.4, 112.2, 114.1, 114.6, 114.7, 115.2, 116.2, 121.2, 121.4, 122.0, 127.4–129.1 (m), 133.5, 134.4, 136.7, 137.36, 137.39, 137.60, 137.64, 137.7, 137.8, 137.9, 138.6, 140.5, 145.4, 146.1, 149.3, 149.4, 149.6, 149.9, 150.28, 150.30, 150.8, 154.5, 155.4, 157.9, 158.0, 159.5, 159.8; IR (neat) 3533, 2923, 2119, 1614, 1510, 1492, 1437, 1418, 1327, 1199, 1150, 1052, 1035, 885, 819, 732, 693 cm^{-1}; HRMS (ESI) calcd for $C_{131}H_{35}D_{77}O_{18}SiSNa$ [(M+Na)$^+$] *m/z* 2133.2064, Found *m/z* 2133.2054.

23: R_f = 0.58 (hexane/EtOAc/CH_2Cl_2 = 4/1/1); $[\alpha]_D^{20}$ = +3.6 (*c* 1.1, $CHCl_3$); ^{1}H NMR (600 MHz, benzene-d_6) δ 0.01 (s, 9H), 3.08 (dd, *J* = 7.8, 13.2 Hz, 1H), 3.68 (dd, *J* = 4.2, 13.2 Hz, 1H), 3.99 (d, *J* = 3.6 Hz, 1H), 4.08 (d, *J* = 3.6 Hz, 1H), 4.41 (ddd, *J* = 4.2, 4.8, 7.8 Hz, 1H), 5.45 (s, 1H), 5.46 (d, *J* = 4.8 Hz, 1H), 5.58

(d, J = 3.6 Hz, 1H), 5.61 (d, J = 3.6 Hz, 1H), 6.19 (d, J = 2.4 Hz, 1H), 6.37 (d, J = 2.4 Hz, 1H), 6.38 (d, J = 2.4 Hz, 1H), 6.44 (d, J = 2.4 Hz, 1H), 6.67–6.69 (m, 1H), 6.70 (s, 1H), 6.86–6.91 (m, 3H), 6.95 (d, J = 8.4 Hz, 1H), 6.99 (d, J = 8.4 Hz, 1H), 7.05 (dd, J = 1.8, 7,8 Hz, 1H), 7.09 (d, J = 1.8 Hz, 1H), 7.31 (dd, J = 1.2, 8.4 Hz, 1H), 7.32 (d, J = 7.8 Hz, 1H), 7.55 (dd, J = 2.4, 8.4 Hz, 1H), 7.64 (dd, J = 1.8, 7.8 Hz, 1H), 7.70 (d, J =2.4 Hz, 1H), 7.85 (d, J = 1.8 Hz, 1H); ^{13}C NMR (150 MHz, benzene-d_6) δ 0.4, 26.3, 26.6, 37.4, 68.9–70.9 (m), 74.0, 74.6, 75.5, 83.6, 95.3, 95.6, 96.1, 97.2, 98.9, 99.4, 105.6, 105.9, 108.1, 109.7, 112.7, 114.5, 114.7, 115.3, 115.4, 116.1, 120.9, 121.2, 121.4, 126.08, 127.2–128.6 (m), 129.21, 129.23, 130.1, 133.5, 133.8, 136.1, 137.1, 137.3, 137.4, 137.56, 137.60, 137.69, 137.73, 138.0, 138.3, 146.0, 146.5, 149.2, 149.3, 150.1, 150.2, 150.3, 153.2, 154.4, 154.8, 155.3, 157.7, 158.0, 159.51, 159.54; IR (neat) 3527, 2956, 2205, 1615, 1597, 1509, 1489, 1439, 1418, 1328, 1327, 1200, 1183, 1140, 1085, 1037, 960, 840, 812, 742, 691 cm^{-1}; HRMS (ESI) calcd for $C_{131}H_{37}D_{77}O_{18}SiSNa$ [(M+Na)$^+$] m/z 2135.2220, Found m/z 2133.2238.

Union of trimer 22 with bottom EC unit 24

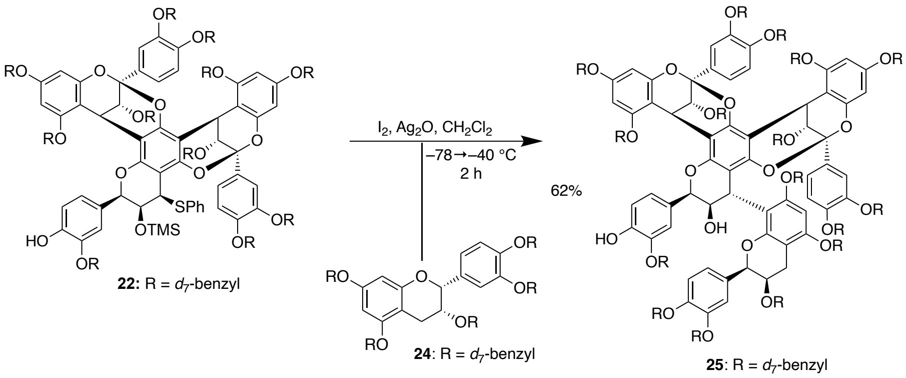

To a solution of sulfide **22** (40 mg, 0.019 mmol) and epicatechin derivative **24** (29 mg, 0.038 mmol) in CH_2Cl_2 (3 mL) was added Ag_2O (11 mg, 0.047 mmol) and solution of I_2 (6.2 mg, 0.024 mmol) in CH_2Cl_2 (1 mL) at –78 °C. The reaction mixture was stirred at the same temperature for 1 h before warming up to –40 °C over 30 min and stirring was continued for additional 30 min. The reaction was quenched by adding aqueous 10% $Na_2S_2O_3$ solution and sat. aqueous $NaHCO_3$ solution. The crude products were extracted with EtOAc (x3). The combined organic extracts were successively washed with water, brine, dried (Na_2SO_4) and concentrated in vacuo. The residue was purified by preparative TLC (silica-gel, hexane/EtOAc/CH_2Cl_2 = 2/1/1) to afford tetramer **25** (32 mg, 62%) as an ivory foam. R_f = 0.25 (hexane/EtOAc/CH_2Cl_2 = 4/1/1); $[\alpha]_D^{20}$ = +57 (c 0.47, $CHCl_3$); ^{1}H NMR (600 MHz, $CDCl_3$) δ 2.09 (d, J = 8.4 Hz, 1H), 2.75 (dd, J = 4.2, 17.4 Hz, 1H), 3.06 (brs, 1H), 3.21 (d, J = 17.4 Hz, 1H), 3.53 (d, J = 3.6 Hz, 1H), 3.60 (d, J = 3.6 Hz, 1H), 4.02 (s, 1H), 4.25 (d, J = 6.6 Hz, 1H), 4.60 (brs, 1H), 4.98 (d, J = 3.6 Hz, 1H), 5.06 (d, J = 3.6 Hz, 1H), 5.46 (s, 1H), 5.84–5.86 (m, 3H), 5.88 (d, J = 2.4 Hz, 1H), 5.99 (d, J = 2.4 Hz, 1H), 6.02 (d, J = 1.8 Hz, 1H), 6.19 (brs, 1H), 6.23 (brs, 1H), 6.45 (d, J = 7.8 Hz, 1H), 6.49 (d, J = 8.4 Hz, 1H), 6.57 (d, J = 7.8 Hz, 1H),

6.94 (d, J = 8.4 Hz, 1H), 7.14–7.17 (m, 3H); 7.28 (brs, 1H), 7.37 (d, J = 1.8 Hz, 1H); ^{13}C NMR (150 MHz, $CDCl_3$) δ 25.6, 25.2, 25.8, 29.9, 68.3–71.4 (m), 72.4, 72.5, 72.8, 73.2, 79.0, 92.9, 94.0, 94.8, 95.1, 95.2, 97.8, 98.7, 104.9, 105.3, 105.7, 106.1, 111.2, 112.0, 112.2, 112.8, 113.8, 114.0, 114.2, 115.5, 116.7, 118.1, 119.5, 120.8, 121.3, 126.6–128.3 (m), 131.8, 132.8, 133.8, 136.0, 136.6, 136.7, 136.88, 136.94, 136.97, 138.04, 137.1, 137.2, 137.3, 137.4, 137.5, 137.6, 138.2, 138.5, 138.8, 145.5, 145.8, 147.6, 147.8, 148.0, 148.1, 148.3, 148.6, 148.7, 149.2, 150.1, 153.9, 154.3, 154.5, 156.4, 156.5, 157.3, 157.8, 158.4, 158.8; IR (neat) 3546, 2924, 2204, 2118, 1610, 1594, 1508, 1438, 1327, 1271, 1201, 1182, 1121, 1086, 1035, 988, 909, 838, 812, 732, 647 cm^{-1}; HRMS (ESI) calcd for $C_{172}H_{30}D_{112}O_{24}Na$ [$(M+Na)^{-}$] *m/z* 2726.6813, Found *m/z* 2726.6822.

Synthesis of 1

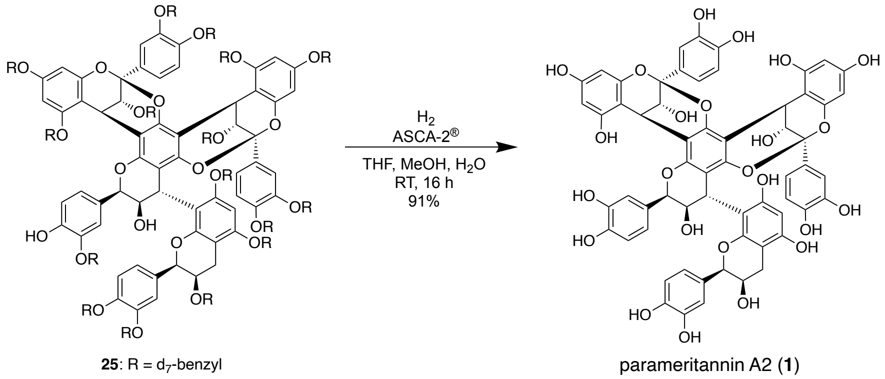

A solution of tetramer **25** (40.2 mg, 0.0149 mmol) in the presence of ASCA-2® (100 mg, 0.0357 mmol) in a mixture of THF, MeOH and H_2O (v/v/v = 2/2/1, 35 mL) was stirred under H_2 atmosphere at room temperature. After stirring for 16 h, the mixture was carefully filtered through a glass filter (MeOH) under argon atmosphere and the filtrate was evaporated to remove organic solvents and lyophilized to give a crude material, which was further purified by preparative HPLC [InertSustain® C18, 20 mm ϕ × 250 mm, MeOH, H_2O (35/65) containing 0.1% TFA, flow rate 8 mL/min, detected at 280 nm] and subsequent lyophilization gave **1** (15.5 mg, 91%) as an ivory amorphous solid. $[\alpha]_D^{28}$ = +13 (*c* 0.50, CH_3OH); IR (neat) 3243, 1671, 1609, 1520, 1443, 1371, 1282, 1197, 1064, 1004, 971, 945, 853, 819, 785, 639 cm^{-1}; HRMS (ESI) calcd for $C_{60}H_{45}O_{24}$ [$(M-H)^{+}$] *m/z* 1149.2306, Found *m/z* 1149.2310.

HPLC analysis of Paramerítannin A2

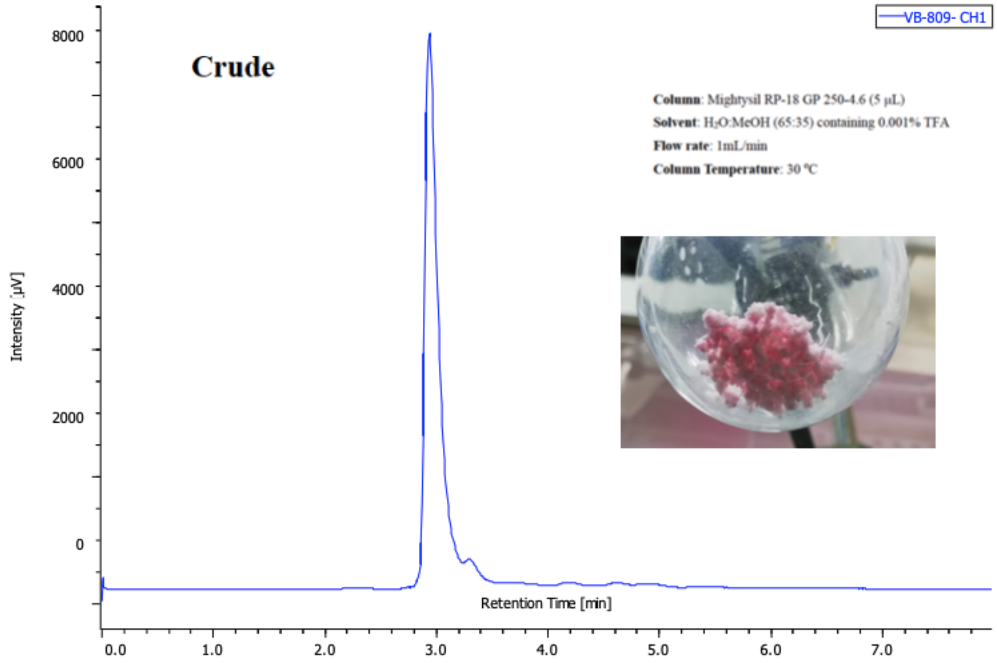

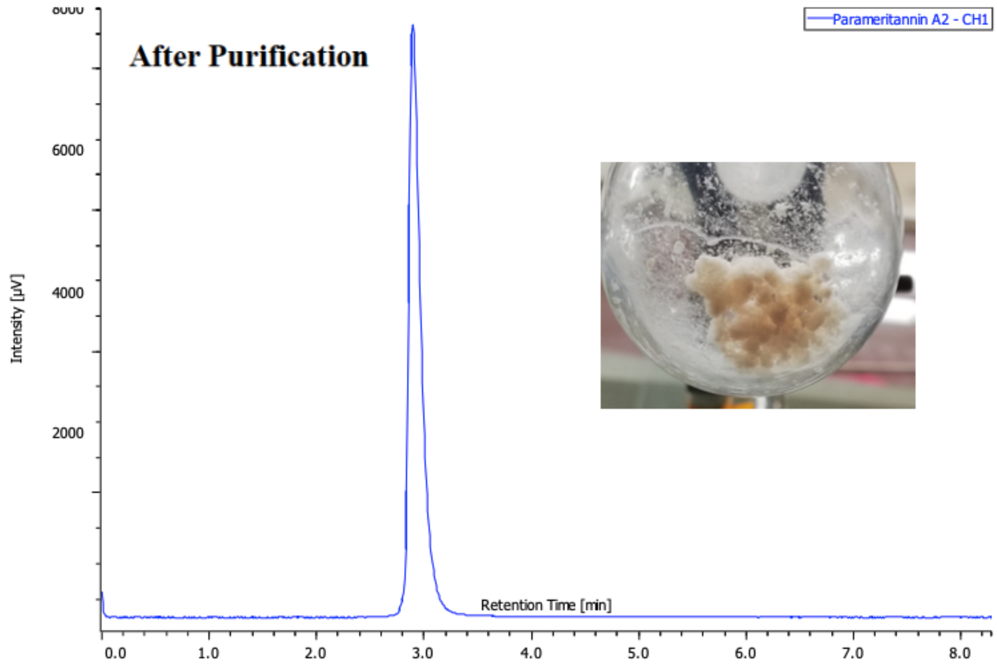

^{1}H and ^{13}C NMR data of Parameritannin A2 (1)

Ring	Carbon number	Natural (*lit.*)[7]		Synthetic	
		^{13}C (100 MHz)	^{1}H (400 MHz)	^{13}C (150MHz)	^{1}H (600MHz)
**Upper unit**					
C	C-2	100.80	—	100.81	—
	C-3	66.43	3.45 (d, *J* = 3.5 Hz)	66.41	3.45 (d, *J* = 3.0 Hz)
	C-4	28.86	4.25 (d, *J* = 3.5 Hz)	28.86	4.24 (d, *J* = 3.0 Hz)
A	C-5	157.76	—	157.77	—
	C-6	98.69	5.92 (d, *J* = 2.4 Hz)	98.63	5.92 (d, *J* = 1.8 Hz)
	C-7	157.90[a]	—	157.91[a]	—
	C-8	96.78	6.04 (d, *J* = 2.4 Hz)	96.52	6.03 (d, *J* = 1.8 Hz)
	C-9	153.66	—	153.62	—
	C-10	104.19	—	104.17	—
B	C-1’	131.24	—	131.26	—
	C-2’	115.62	7.12 (d, *J* = 2.2 Hz)	115.63	7.12 (d, *J* = 1.8 Hz)
	C-3’	145.68	—	145.69	—
	C-4’	147.01	—	147.03	—
	C-5’	116.11	6.87 (d, *J* = 8.4 Hz)	116.03	6.87 (d, *J* = 8.4 Hz)
	C-6’	119.77	6.94 (dd, *J* = 2.2, 8.4 Hz)	119.74	6.93 (dd, *J* = 1.8, 8.4 Hz)
**Middle Unit**					
F	C-2	78.78	5.59 (brs)	78.81	5.59 (brs)
	C-3	72.14	4.09 (brd, *J* = 1.6 Hz)	72.17	4.08 (brd, *J* =1.8 Hz)
	C4	38.81	4.50 (brd, *J* = 1.6 Hz)	38.83	4.49 (br)
D	C-5	149.71	—	149.73	—
	C-6	107.04	—	107.06	—
	C-7	145.54	—	145.54	—
	C-8	108.08	—	108.09	—
	C-9	150.27	—	150.29	—
	C-10	107.64	—	107.66	—
E	C-1’	131.52	—	131.53	—
	C-2’	116.66	7.29 (d, *J* = 2.0 Hz)	116.67	7.29 (d, *J* = 2.4 Hz)
	C-3’	145.81	—	145.83	—
	C-4’	146.26	—	146.30	—
	C-5’	116.11	6.80 (d, *J* = 8.3 Hz)	116.03	6.80 (d, *J* = 8.4 Hz)
	C-6’	121.29	7.17 (dd, *J* = 2.0, 8.3 Hz)	121.30	7.17 (dd, *J* = 2.4, 8.4 Hz)
**Lower Unit**					
I	C-2	80.56	4.40 (brs)	80.58	4.40 (brs)
	C-3	67.57	3.85 (m)	67.60	3.85 (m)
	C-4	29.97	2.94 (dd, *J* = 4.5, 17.4)<br>2.88 (dd, *J* = 1.6, 17.2)	30.00	2.94 (dd, *J* = 4.2, 16.8 Hz)<br>2.88 (dd, *J* = 1.8, 16.2 Hz)
G	C-5	156.32	—	156.35	—
	C-6	97.41	6.09 (s)	97.33	6.09 (s)

	C-7	155.85	—	155.90	—
	C-8	109.00	—	108.96	—
	C-9	155.59	—	155.63	—
	C-10	100.20[b]	—	100.15[b]	—
H	C-1’	132.89	—	132.91	—
	C-2’	115.29	6.81 (d, J = 1.9 Hz)	115.27	6.81 (d, J = 1.8 Hz)
	C-3’	145.74	—	145.76	—
	C-4’	145.45	—	145.47	—
	C-5’	116.02	6.78 (d, J = 8.2 Hz)	115.94	6.78 (d, J = 8.4 Hz)
	C-6’	119.10	6.67 (dd, J = 1.9, 8.2 Hz)	119.06	6.67 (dd, J = 1.8, 8.4 Hz)
**Side (Branched) Unit**					
L	C-2	100.23[b]	—	100.24[b]	—
	C-3	67.96	3.80 (d, J = 3.6 Hz)	67.97	3.79 (d, J = 3.6 Hz)
	C-4	29.45	4.01 (d, J = 3.6 Hz)	29.46	4.01 (d, J = 3.6 Hz)
J	C-5	156.20	—	156.22	—
	C-6	98.42	5.81 (d, J =2.4 Hz)	98.36	5.80 (d, J =2.4 Hz)
	C-7	157.87[a]	—	157.88[a]	—
	C-8	96.57	5.93 (d, J =2.4 Hz)	96.52	5.93 (d, J =2.4 Hz)
	C-9	154.06	—	154.03	—
	C-10	103.87	—	103.85	—
K	C-1’	131.85	—	131.87	—
	C-2’	116.54	6.92 (d, J = 2.2 Hz)	116.55	6.92 (d, J = 2.4 Hz)
	C-3’	144.73	—	144.74	—
	C-4’	146.19	—	146.22	—
	C-5’	115.96	6.50 (d, J = 8.3 Hz)	115.94	6.50 (d, J = 8.4 Hz)
	C-6’	119.60	5.94 (dd, J = 2.2, 8.3 Hz)	119.58	5.94 (dd, J = 2.4, 8.4 Hz)

NMR solvent = CD_3OD. Symbols *a* and *b* in each column may be interchanged.

References:

1. V. V. Betkekar, M. Harachi, K. Suzuki, K. Ohmori, *Org. Biol. Chem.* **2019**, *17*, 9129–9134.
2. L. I. Pilkington, J. Wagoner, S. J. Polyak, D. Barker, *Org. Lett*. **2015**, *17*, 1046–1049.
3. G. Zhang, Y. Jing, D. C. Myles, Y. Li, Y. Chen, *Chin. J. Chem*. **2013**, *31*, 773–778.
4. S. Narasimhan, S. Madhavan, K. G. Prasad, *J. Org. Chem*. **1995**, *60*, 5314–5315.
5. a) E. Brunet, J. L. García Ruano, M. A. Hoyos, J. H. Rodríguez, P. Prados, F. Alcudia, *Org. Magn. Reson.* **1983**, *23*, 643–648; b) J. C. Carretero, J. L. García Ruano, M. C. Martinez, J. H. Rodríguez, *Tetrahedron* **1985**, *41*, 2419–2433; c) E. Brunet, J. L. García Ruano, J. H. Rodríguez, M. A. Secundíno, J. M. García de la Vega, *J. Mol. Struct*. **1986**, *144*, 109–119; d) M. C. Carreño, J. L. García Ruano, A. M. Martín, C. Pedregal, J. H. Rodriguez, A. Rubio, J. Sanchez, G. Solladié, *J. Org. Chem.* **1990**, *55*, 2120–2128.
6. a) J. L. García Ruano, C. Pedregal, J. H. Rodríguez, *Tetrahedron* **1987**, *43*, 4407–4416; b) C. Alvarez-Ibarra, R. Cuervo-Rodríguez, M. C. Fernández-Monreal, M. P. Ruiz, *J. Org. Chem*. **1994**, *59*, 7284–7291.
7. K. Kamiya, C. Watanabe, H. Endang, M. Umar, T. Satake, *Chem. Pharm. Bull*. **2001**, *49*, 551–557.

^{1}H NMR of 8 (600MHz, $CDCl_3$)

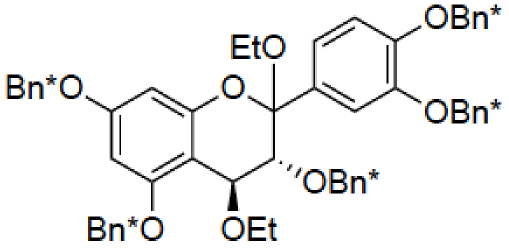

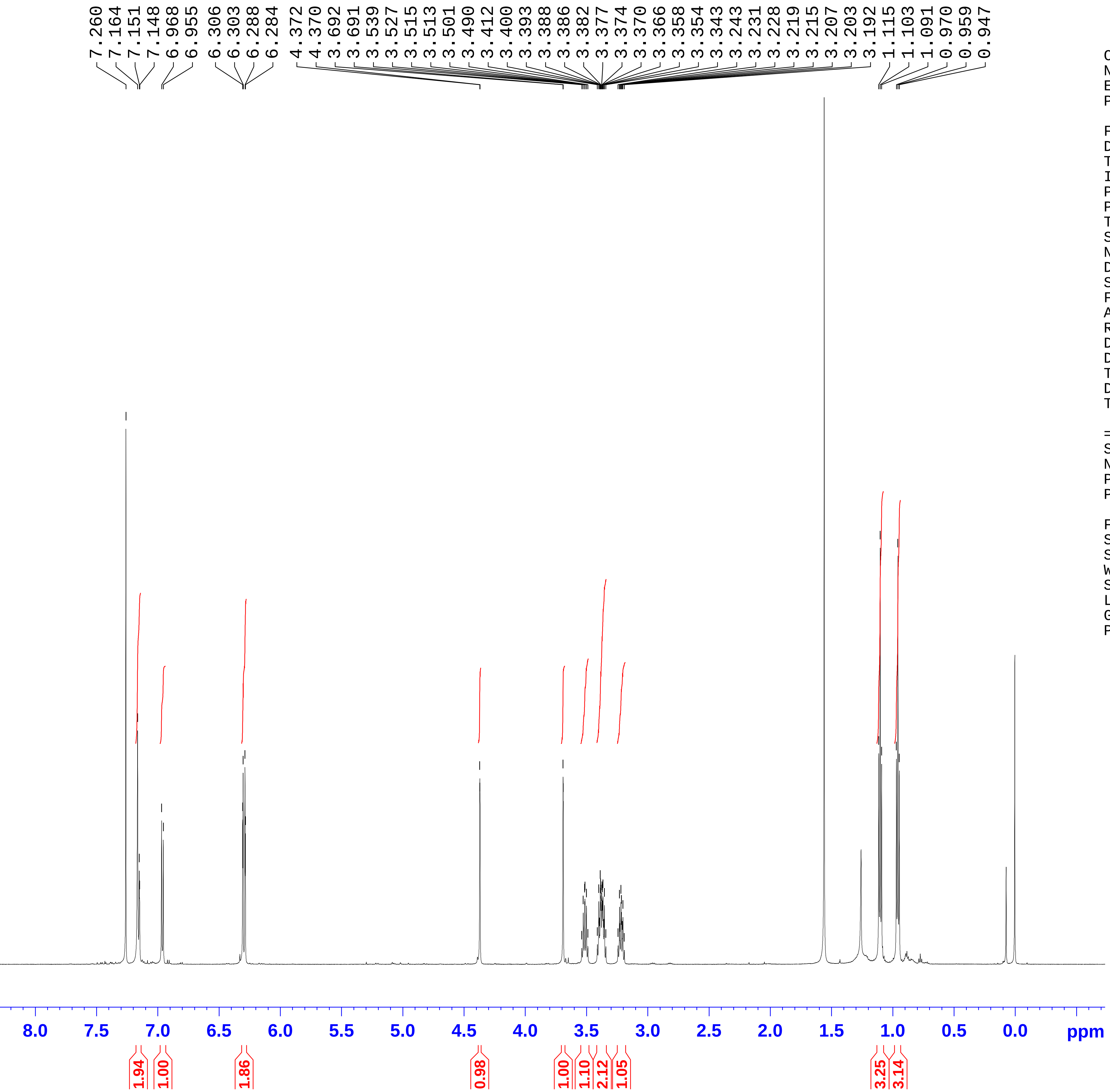

Current Data Parameters
NAME VB-312
EXPNO 10
PROCNO 1

F2 - Acquisition Parameters
Date_ 20190530
Time 21.49
INSTRUM spect
PROBHD 5 mm CPPBBO BB
PULPROG zg30
TD 65536
SOLVENT CDCl3
NS 16
DS 2
SWH 12019.230 Hz
FIDRES 0.183399 Hz
AQ 2.7262976 sec
RG 31.94
DW 41.600 usec
DE 10.00 usec
TE 298.1 K
D1 1.00000000 sec
TD0 1

======== CHANNEL f1 ========
SFO1 600.1337060 MHz
NUC1 1H
P1 12.00 usec
PLW1 21.00000000 W

F2 - Processing parameters
SI 65536
SF 600.1300148 MHz
WDW EM
SSB 0
LB 0.30 Hz
GB 0
PC 1.00

^{13}C NMR of 8 (150MHz, $CDCl_3$)

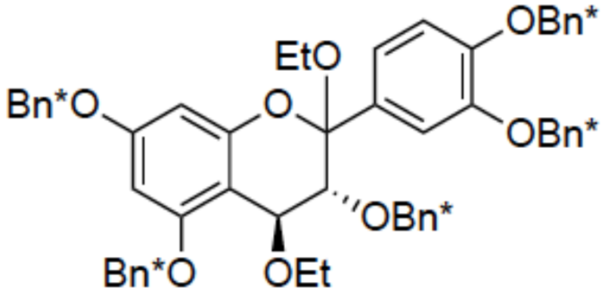

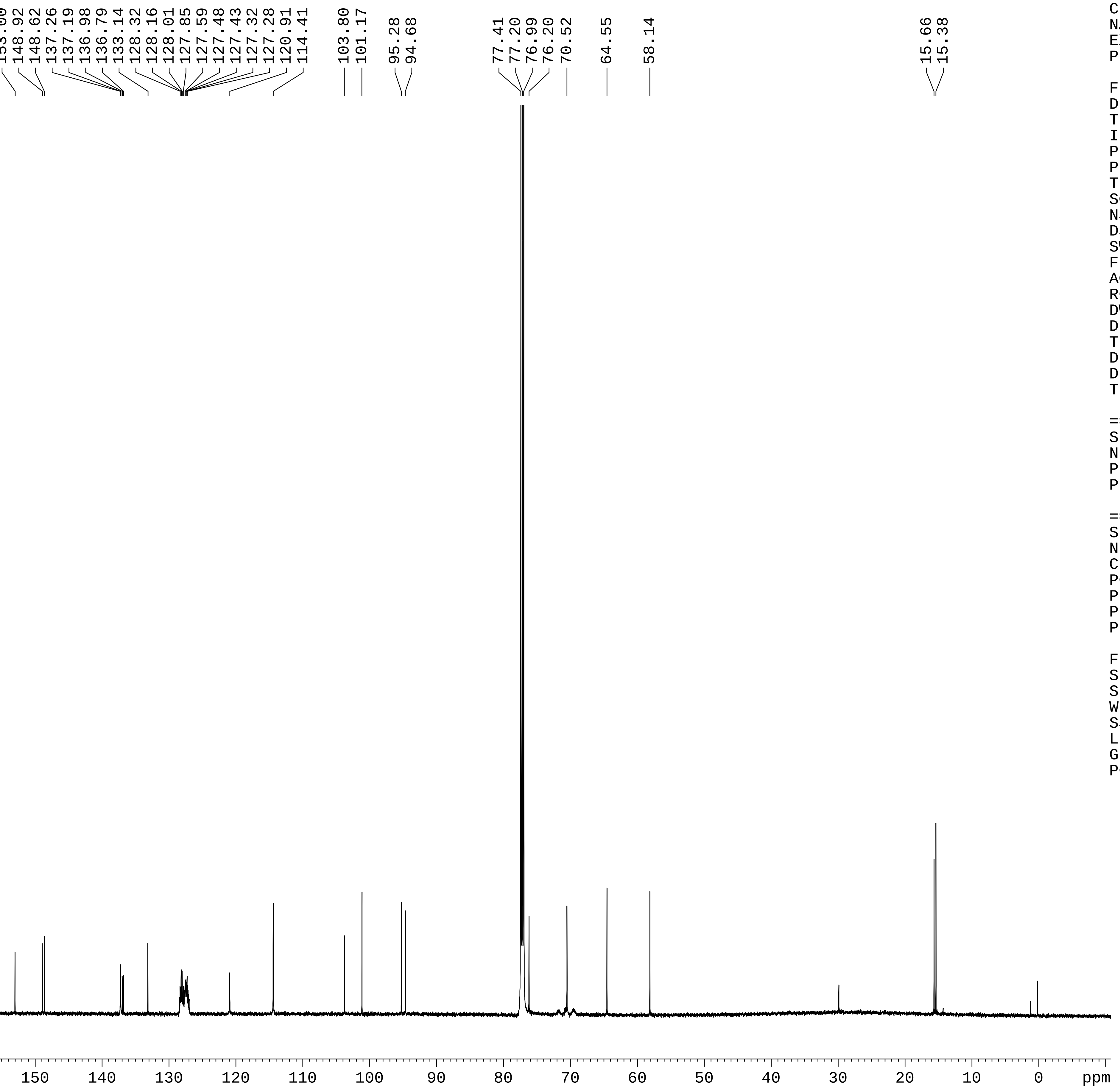

BRUKER

```
Current Data Parameters
NAME              VB-312
EXPNO                 11
PROCNO                 1

F2 - Acquisition Parameters
Date_           20190531
Time                3.25
INSTRUM            spect
PROBHD    5 mm CPPBBO BB
PULPROG          zgpg30
TD                 65536
SOLVENT            CDCl3
NS                  4000
DS                     4
SWH            36057.691 Hz
FIDRES          0.550197 Hz
AQ             0.9087659 sec
RG                175.56
DW                13.867 usec
DE                 18.00 usec
TE                 298.1 K
D1            2.00000000 sec
D11           0.03000000 sec
TD0                    1

======== CHANNEL f1 ========
SFO1         150.9178981 MHz
NUC1                 13C
P1                 10.00 usec
PLW1         80.00000000 W

======== CHANNEL f2 ========
SFO2         600.1324005 MHz
NUC2                  1H
CPDPRG[2         waltz16
PCPD2              70.00 usec
PLW2         13.43999958 W
PLW12         0.61714000 W
PLW13         0.31042001 W

F2 - Processing parameters
SI                 32768
SF           150.9027830 MHz
WDW                   EM
SSB       0
LB                  1.00 Hz
GB        0
PC                  1.40
```

^{1}H NMR of 11 (600MHz, $CDCl_3$)

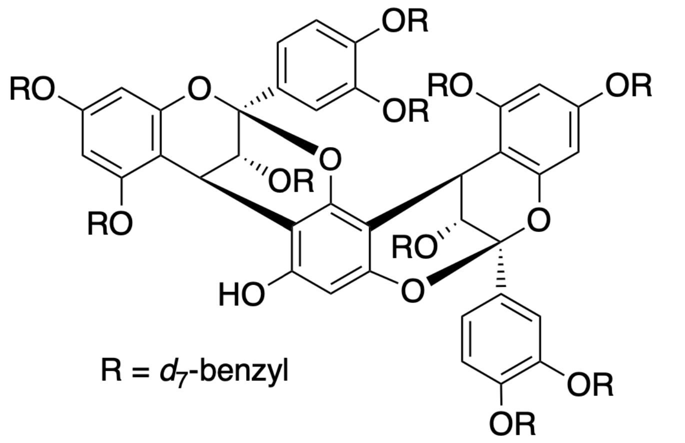

R = d_7-benzyl

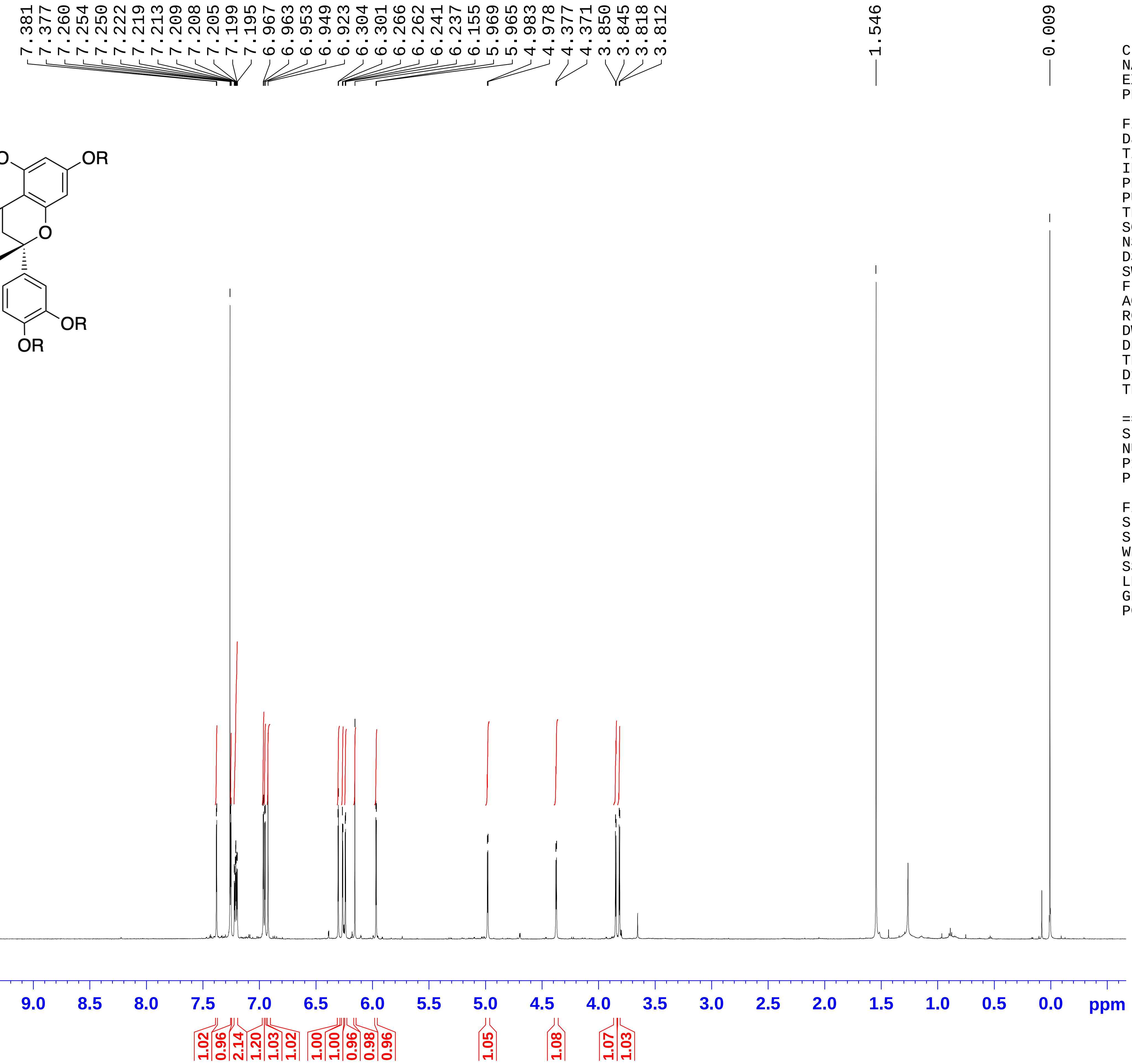


```
Current Data Parameters
NAME              VB-141
EXPNO                 80
PROCNO                 1

F2 - Acquisition Parameters
Date_           20211119
Time               18.21
INSTRUM            spect
PROBHD   5 mm CPPBBO BB
PULPROG             zg30
TD                 65536
SOLVENT            CDCl3
NS                    16
DS                     2
SWH            12019.230 Hz
FIDRES          0.183399 Hz
AQ             2.7262976 sec
RG                  17.5
DW                41.600 usec
DE                 10.00 usec
TE                 298.2 K
D1            1.00000000 sec
TD0                    1

======== CHANNEL f1 ========
SFO1         600.1337060 MHz
NUC1                  1H
P1                 12.00 usec
PLW1         21.00000000 W

F2 - Processing parameters
SI                 65536
SF           600.1300144 MHz
WDW                   EM
SSB      0
LB                  0.30 Hz
GB       0
PC                  1.00
```

^{13}C NMR of 11 (150MHz, $CDCl_3$)

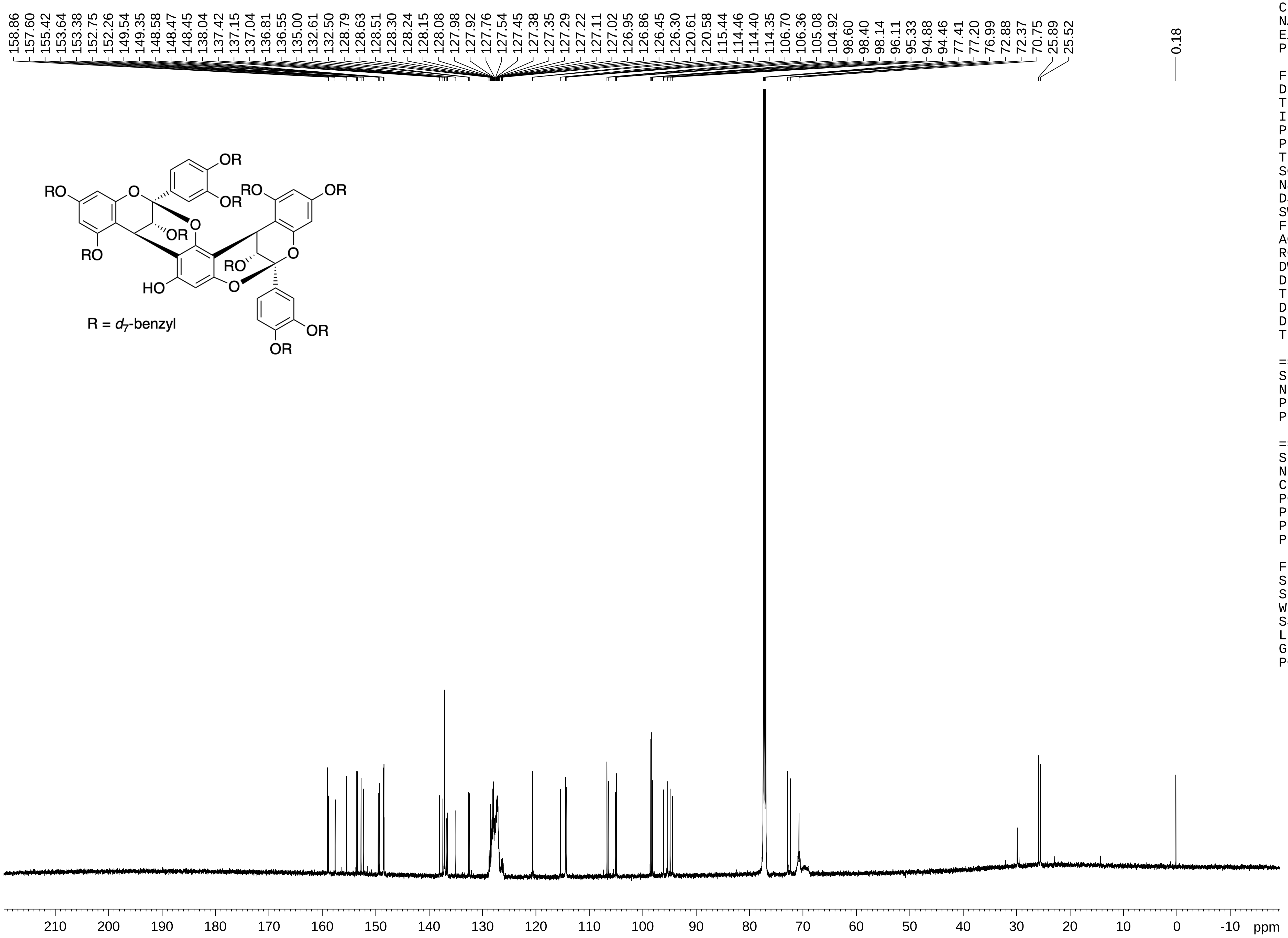


```
Current Data Parameters
NAME                VB-141
EXPNO                   61
PROCNO                   1

F2 - Acquisition Parameters
Date_             20211118
Time                  2.10
INSTRUM              spect
PROBHD   5 mm CPPBBO BB
PULPROG            zgpg30
TD                   65536
SOLVENT              CDCl3
NS                    5000
DS                       4
SWH             36057.691 Hz
FIDRES           0.550197 Hz
AQ              0.9087659 sec
RG                  175.56
DW                  13.867 usec
DE                   18.00 usec
TE                   298.0 K
D1             2.00000000 sec
D11            0.03000000 sec
TD0                      1

======== CHANNEL f1 ========
SFO1          150.9178981 MHz
NUC1                   13C
P1                   10.00 usec
PLW1          80.00000000 W

======== CHANNEL f2 ========
SFO2          600.1324005 MHz
NUC2                    1H
CPDPRG[2           waltz16
PCPD2                70.00 usec
PLW2          13.43999958 W
PLW12          0.61714000 W
PLW13          0.31042001 W

F2 - Processing parameters
SI                   32768
SF            150.9027851 MHz
WDW                     EM
SSB      0
LB                    1.00 Hz
GB       0
PC                    1.40
```

^{1}H NMR of SI-2 (600MHz, $CDCl_3$)

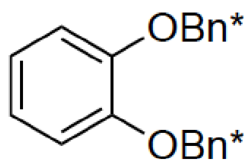

Bn* = d_7-benzyl

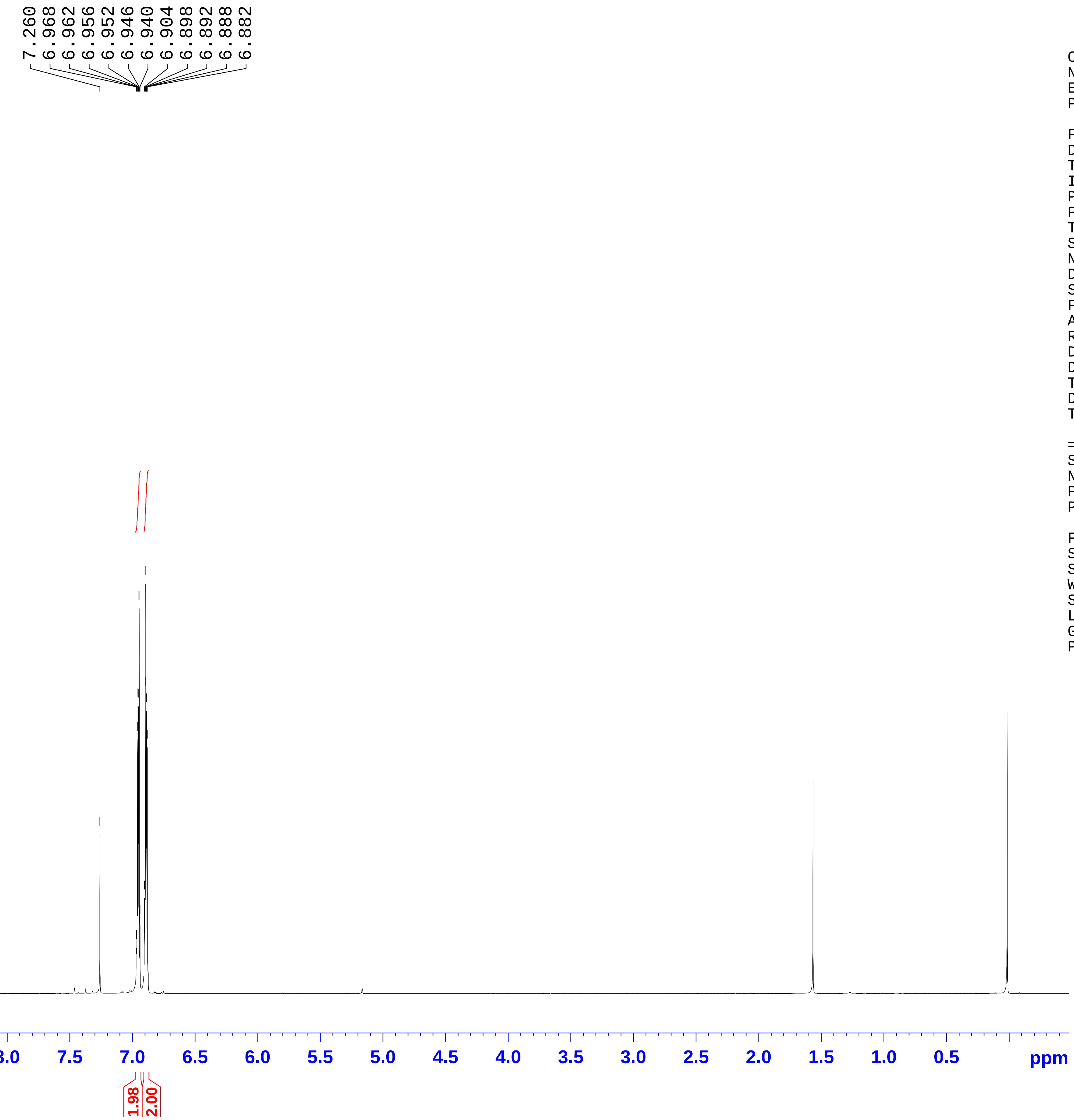

Current Data Parameters

NAME	VB-441-1
EXPNO	10
PROCNO	1

F2 - Acquisition Parameters

Date_	20191003
Time	14.54
INSTRUM	spect
PROBHD	5 mm CPPBBO BB
PULPROG	zg30
TD	65536
SOLVENT	CDCl3
NS	16
DS	2
SWH	12019.230 Hz
FIDRES	0.183399 Hz
AQ	2.7262976 sec
RG	31.94
DW	41.600 usec
DE	10.00 usec
TE	290.7 K
D1	1.00000000 sec
TD0	1

======== CHANNEL f1 ========

SFO1	600.1337060 MHz
NUC1	1H
P1	12.00 usec
PLW1	21.00000000 W

F2 - Processing parameters

SI	65536
SF	600.1300147 MHz
WDW	EM
SSB	0
LB	0.30 Hz
GB	0
PC	1.00

^{13}C NMR of SI-2 (150MHz, $CDCl_3$)

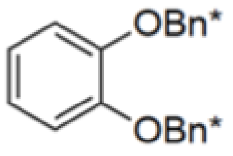

Bn* = d_7-benzyl

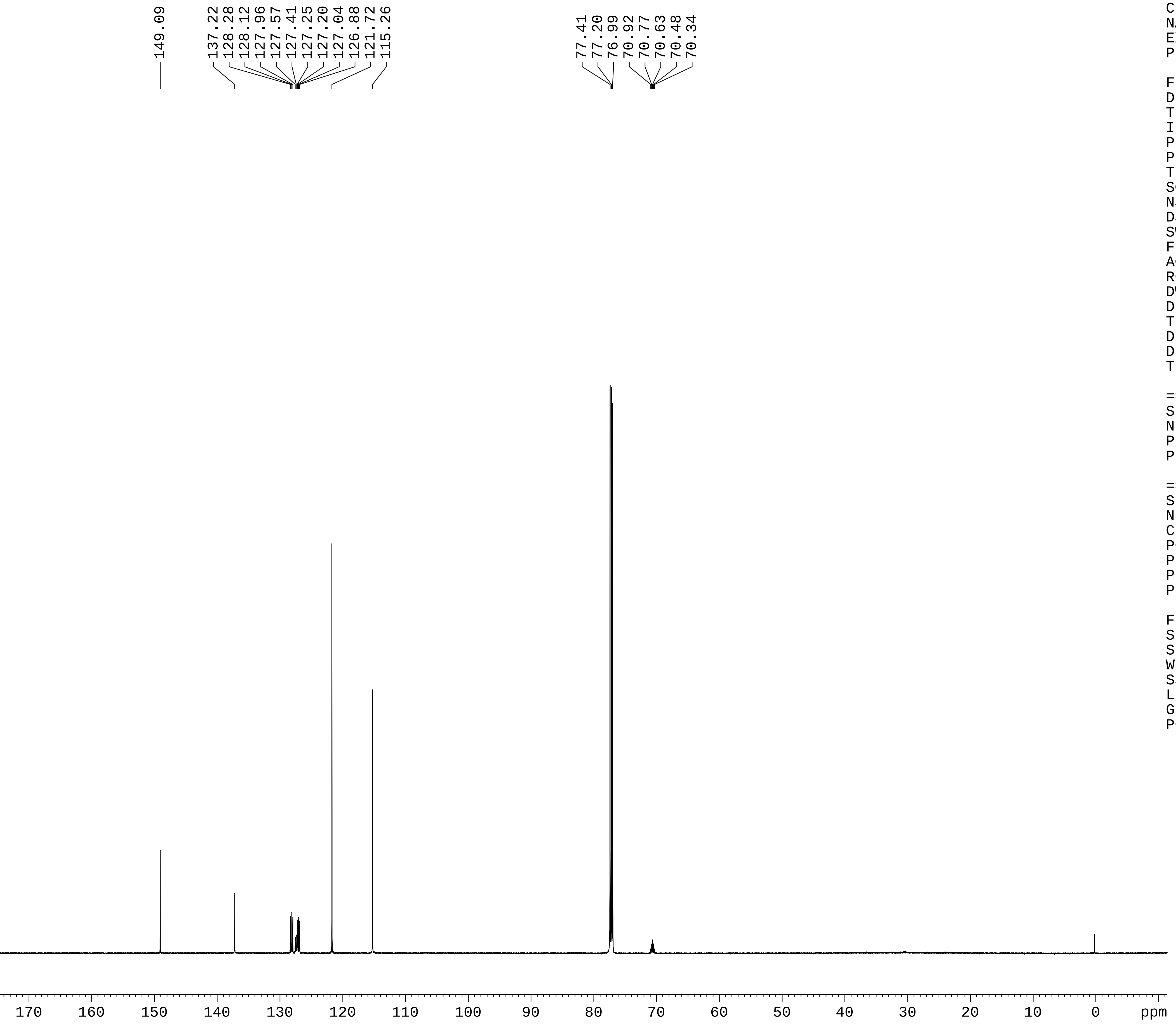

Current Data Parameters
NAME VB-441-1
EXPNO 11
PROCNO 1

F2 - Acquisition Parameters
Date_ 20191004
Time 8.55
INSTRUM spect
PROBHD 5 mm CPPBBO BB
PULPROG zgpg30
TD 65536
SOLVENT CDCl3
NS 1024
DS 4
SWH 36057.691 Hz
FIDRES 0.550197 Hz
AQ 0.9087659 sec
RG 175.56
DW 13.867 usec
DE 18.00 usec
TE 290.1 K
D1 2.00000000 sec
D11 0.03000000 sec
TD0 1

======== CHANNEL f1 ========
SFO1 150.9178981 MHz
NUC1 13C
P1 10.00 usec
PLW1 80.00000000 W

======== CHANNEL f2 ========
SFO2 600.1324005 MHz
NUC2 1H
CPDPRG[2 waltz16
PCPD2 70.00 usec
PLW2 13.43999958 W
PLW12 0.61714000 W
PLW13 0.31042001 W

F2 - Processing parameters
SI 32768
SF 150.9027905 MHz
WDW EM
SSB 0
LB 1.00 Hz
GB 0
PC 1.40

^{1}H NMR of SI-3 (600MHz, $CDCl_3$)

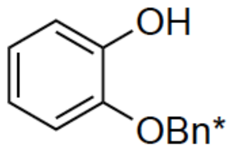

Bn* = d_7-benzyl

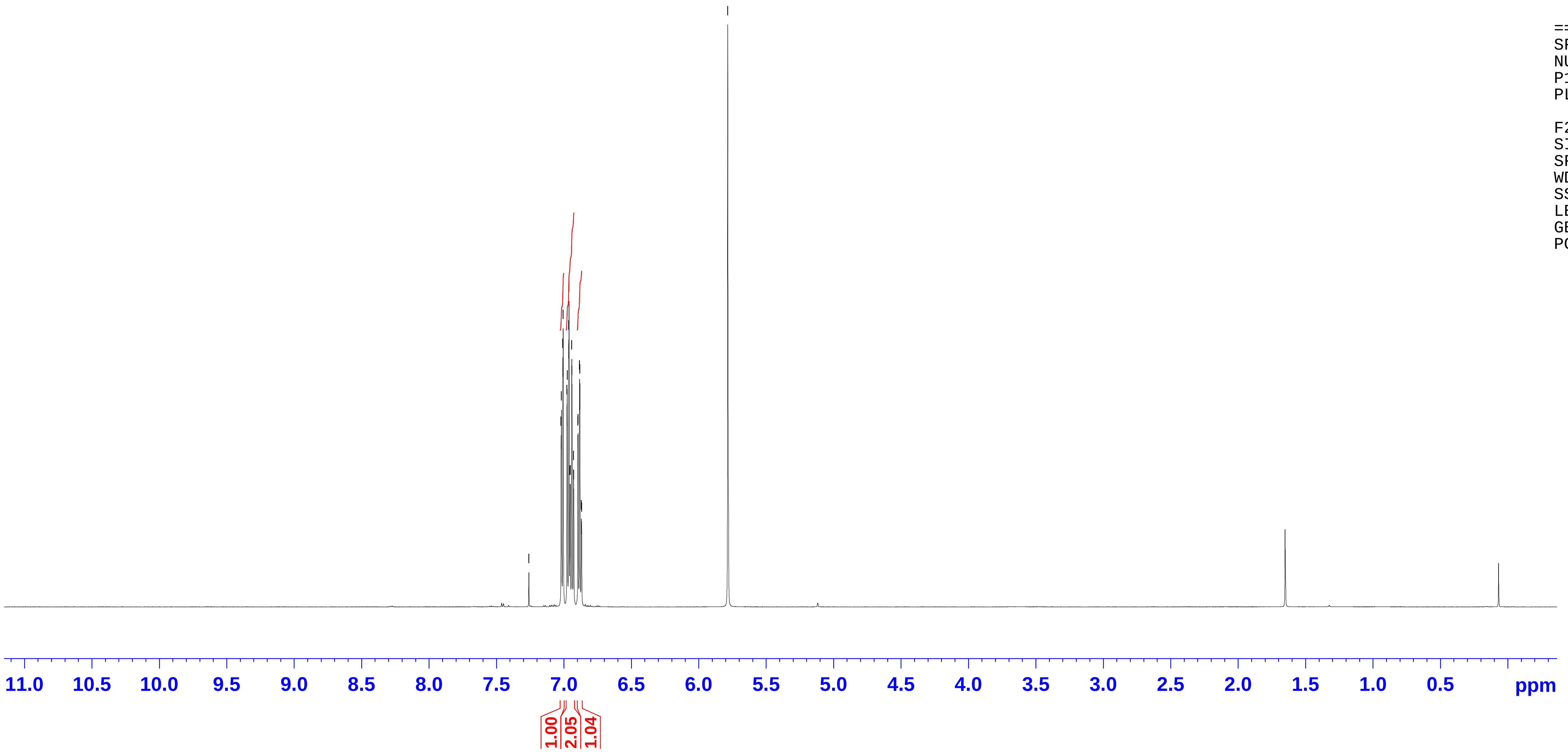


```
Current Data Parameters
NAME              VB-441-2
EXPNO                   20
PROCNO                   1

F2 - Acquisition Parameters
Date_             20191004
Time                 15.03
INSTRUM              spect
PROBHD    5 mm CPPBBO BB
PULPROG               zg30
TD                   65536
SOLVENT              CDCl3
NS                      16
DS                       2
SWH              12019.230 Hz
FIDRES            0.183399 Hz
AQ               2.7262976 sec
RG                    17.5
DW                  41.600 usec
DE                   10.00 usec
TE                   291.2 K
D1              1.00000000 sec
TD0                      1

======== CHANNEL f1 ========
SFO1           600.1337060 MHz
NUC1                    1H
P1                   12.00 usec
PLW1           21.00000000 W

F2 - Processing parameters
SI                   65536
SF             600.1300142 MHz
WDW                     EM
SSB      0
LB                    0.30 Hz
GB       0
PC                    1.00
```

^{13}C NMR of SI-3 (150MHz, $CDCl_3$)

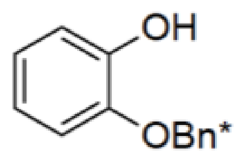

Bn* = d_7-benzyl

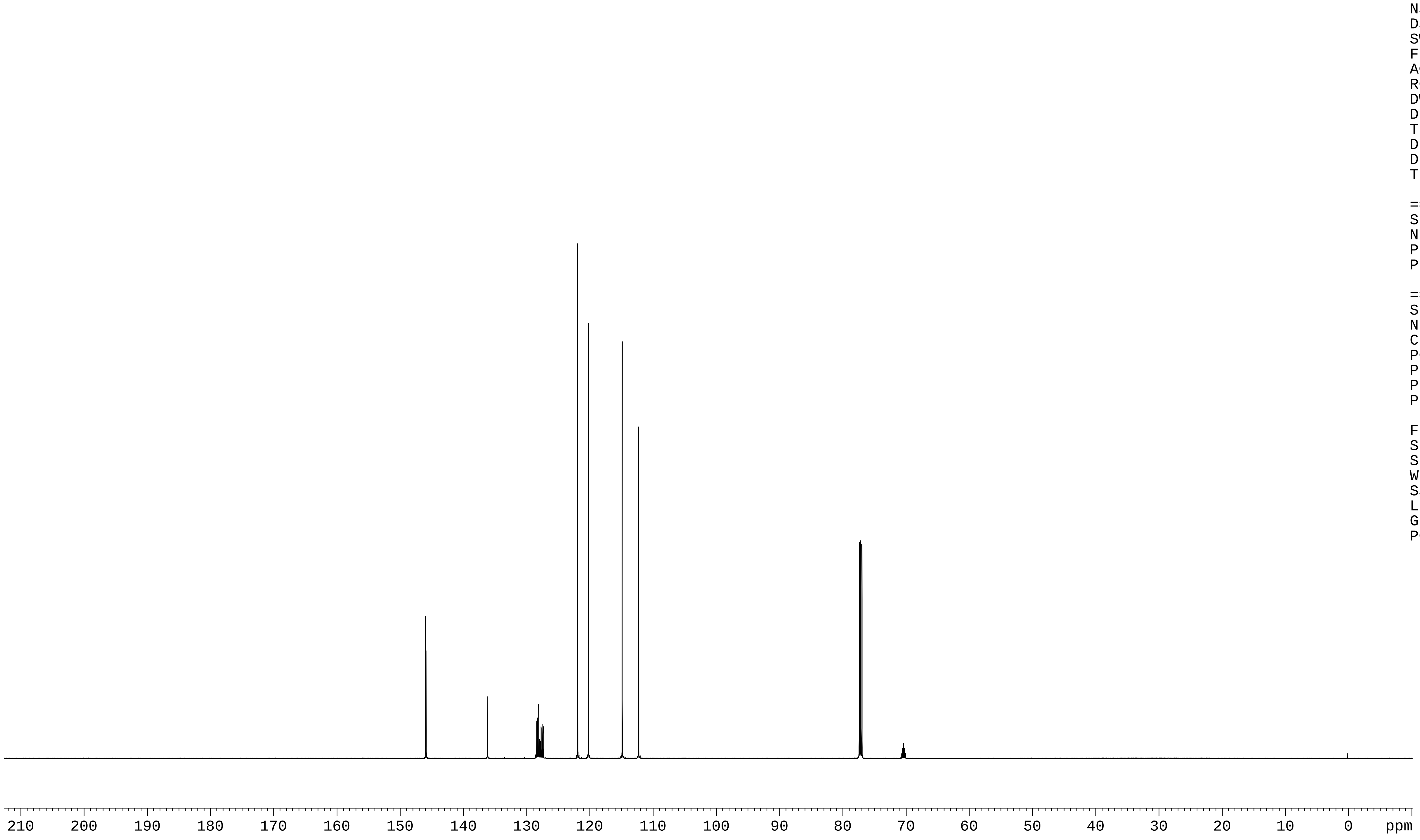

BRUKER

```
Current Data Parameters
NAME               VB-441-2
EXPNO                    21
PROCNO                    1

F2 - Acquisition Parameters
Date_              20191006
Time                   0.55
INSTRUM               spect
PROBHD    5 mm CPPBBO BB
PULPROG             zgpg30
TD                    65536
SOLVENT               CDCl3
NS                     1024
DS                        4
SWH              36057.691 Hz
FIDRES            0.550197 Hz
AQ               0.9087659 sec
RG                   175.56
DW                   13.867 usec
DE                    18.00 usec
TE                    291.3 K
D1               2.00000000 sec
D11              0.03000000 sec
TD0                       1

======== CHANNEL f1 ========
SFO1           150.9178981 MHz
NUC1                    13C
P1                    10.00 usec
PLW1            80.00000000 W

======== CHANNEL f2 ========
SFO2           600.1324005 MHz
NUC2                     1H
CPDPRG[2            waltz16
PCPD2                 70.00 usec
PLW2            13.43999958 W
PLW12            0.61714000 W
PLW13            0.31042001 W

F2 - Processing parameters
SI                    32768
SF             150.9028029 MHz
WDW                      EM
SSB       0
LB                     1.00 Hz
GB        0
PC                     1.40
```

^{1}H NMR of 15 (600MHz, $CDCl_3$)

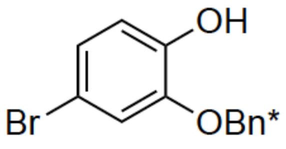

Bn* = d_7-benzyl

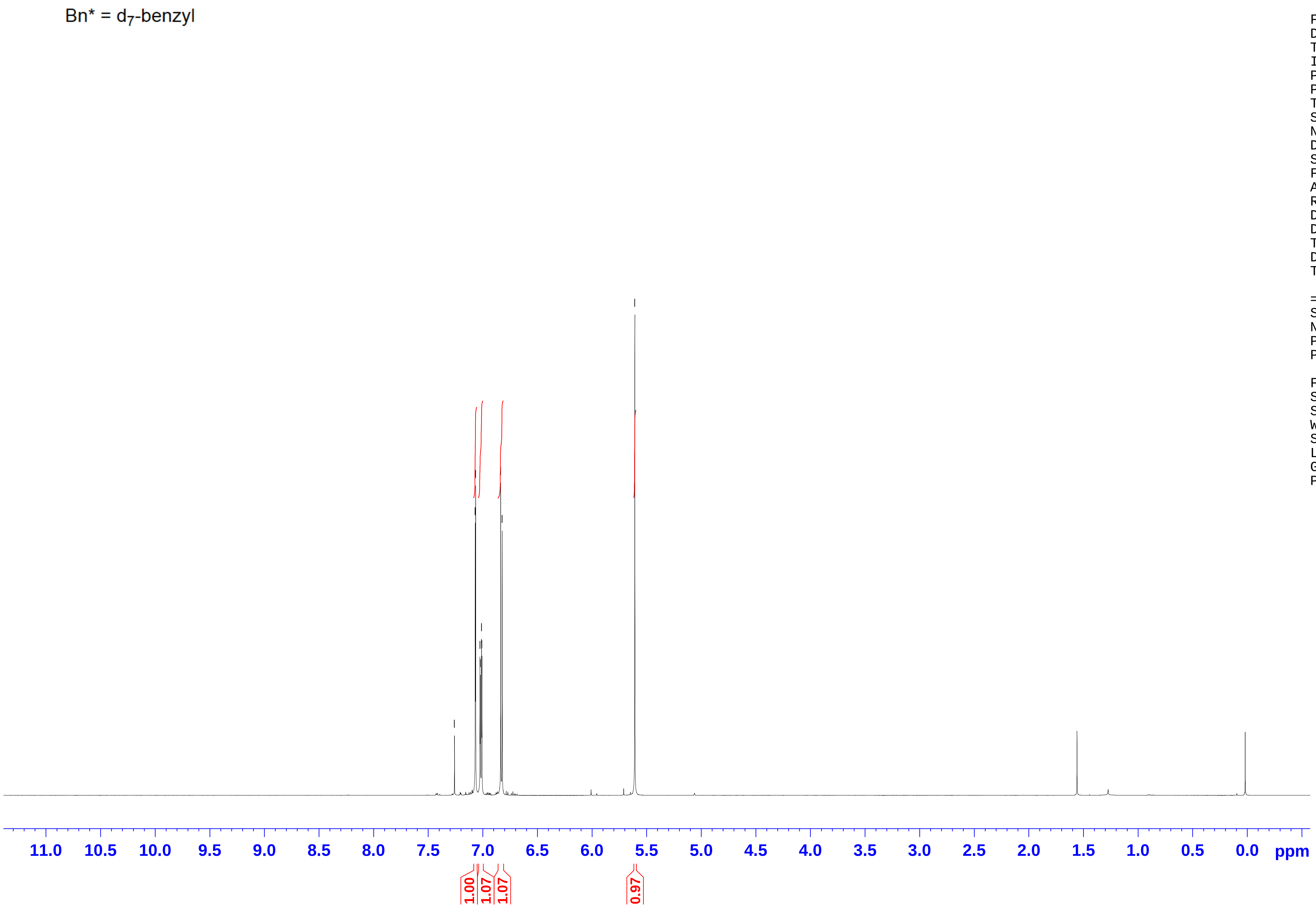


```
Current Data Parameters
NAME              VB-446
EXPNO                 20
PROCNO                 1

F2 - Acquisition Parameters
Date_           20210104
Time               17.56
INSTRUM            spect
PROBHD   5 mm CPPBBO BB
PULPROG             zg30
TD                 65536
SOLVENT            CDCl3
NS                    16
DS                     2
SWH            12019.230 Hz
FIDRES          0.183399 Hz
AQ             2.7262976 sec
RG                 31.94
DW                41.600 usec
DE                 10.00 usec
TE                 298.2 K
D1            1.00000000 sec
TD0                    1

======== CHANNEL f1 ========
SFO1         600.1337060 MHz
NUC1                  1H
P1                 12.00 usec
PLW1         21.00000000 W

F2 - Processing parameters
SI                 65536
SF           600.1300147 MHz
WDW                   EM
SSB      0
LB                  0.30 Hz
GB       0
PC                  1.00
```

^{13}C NMR of 15 (150MHz, $CDCl_3$)

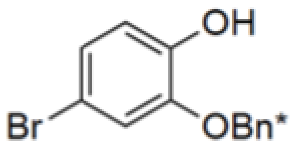

Bn* = d_7-benzyl

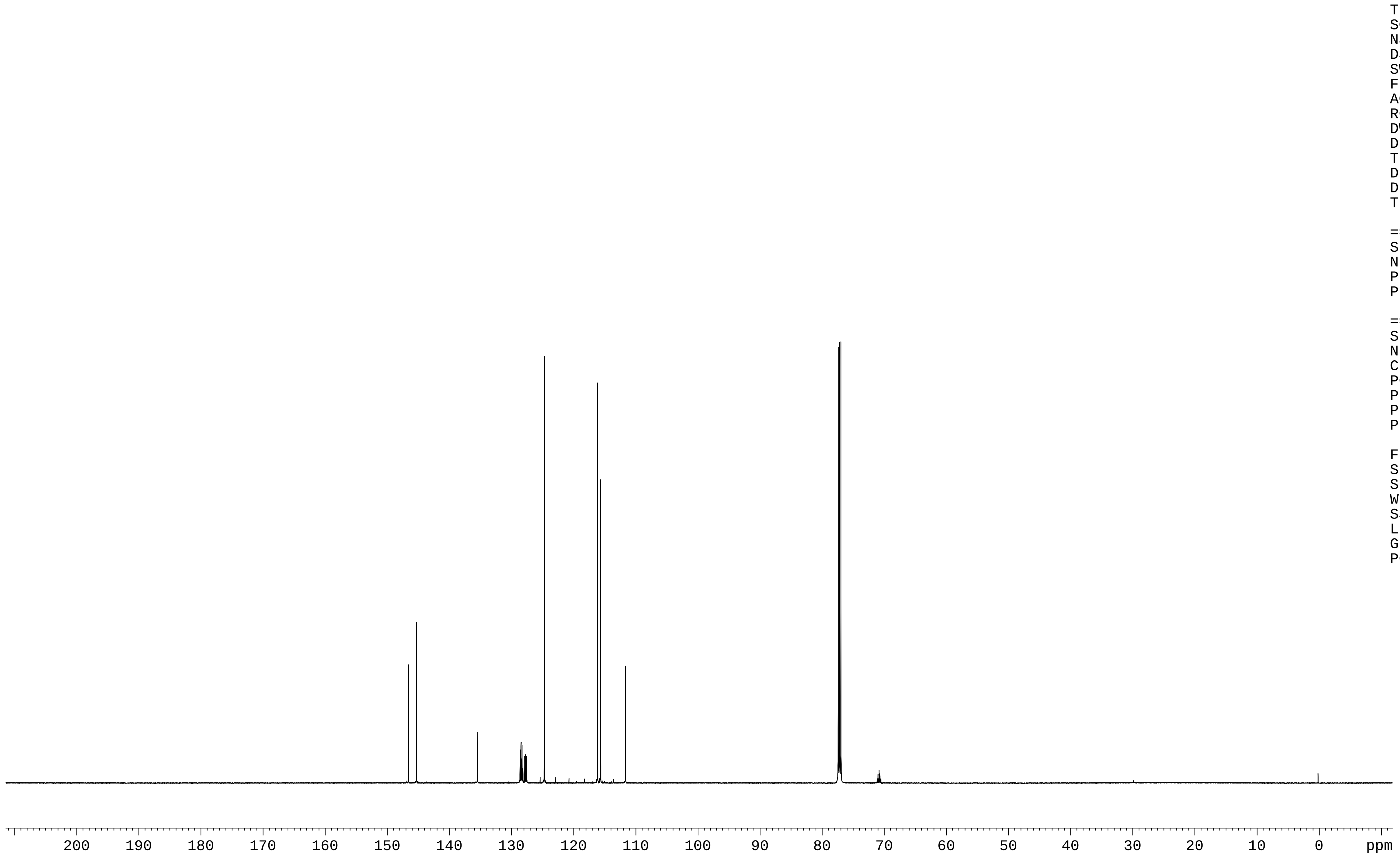


```
Current Data Parameters
NAME             VB-446
EXPNO                21
PROCNO                1

F2 - Acquisition Parameters
Date_          20210105
Time               9.42
INSTRUM           spect
PROBHD   5 mm CPPBBO BB
PULPROG         zgpg30
TD                65536
SOLVENT           CDCl3
NS                 2000
DS                    4
SWH          36057.691 Hz
FIDRES        0.550197 Hz
AQ           0.9087659 sec
RG               175.56
DW               13.867 usec
DE                18.00 usec
TE                298.2 K
D1           2.00000000 sec
D11          0.03000000 sec
TD0                   1

======== CHANNEL f1 ========
SFO1        150.9178981 MHz
NUC1                13C
P1                10.00 usec
PLW1        80.00000000 W

======== CHANNEL f2 ========
SFO2        600.1324005 MHz
NUC2                 1H
CPDPRG[2        waltz16
PCPD2             70.00 usec
PLW2        13.43999958 W
PLW12        0.61714000 W
PLW13        0.31042001 W

F2 - Processing parameters
SI                32768
SF          150.9027875 MHz
WDW                  EM
SSB      0
LB                 1.00 Hz
GB       0
PC                 1.40
```

^{1}H NMR of 17 (600MHz, $CDCl_3$)

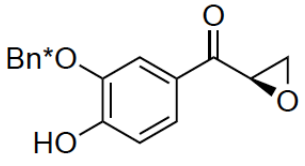

Bn* = d_7-benzyl

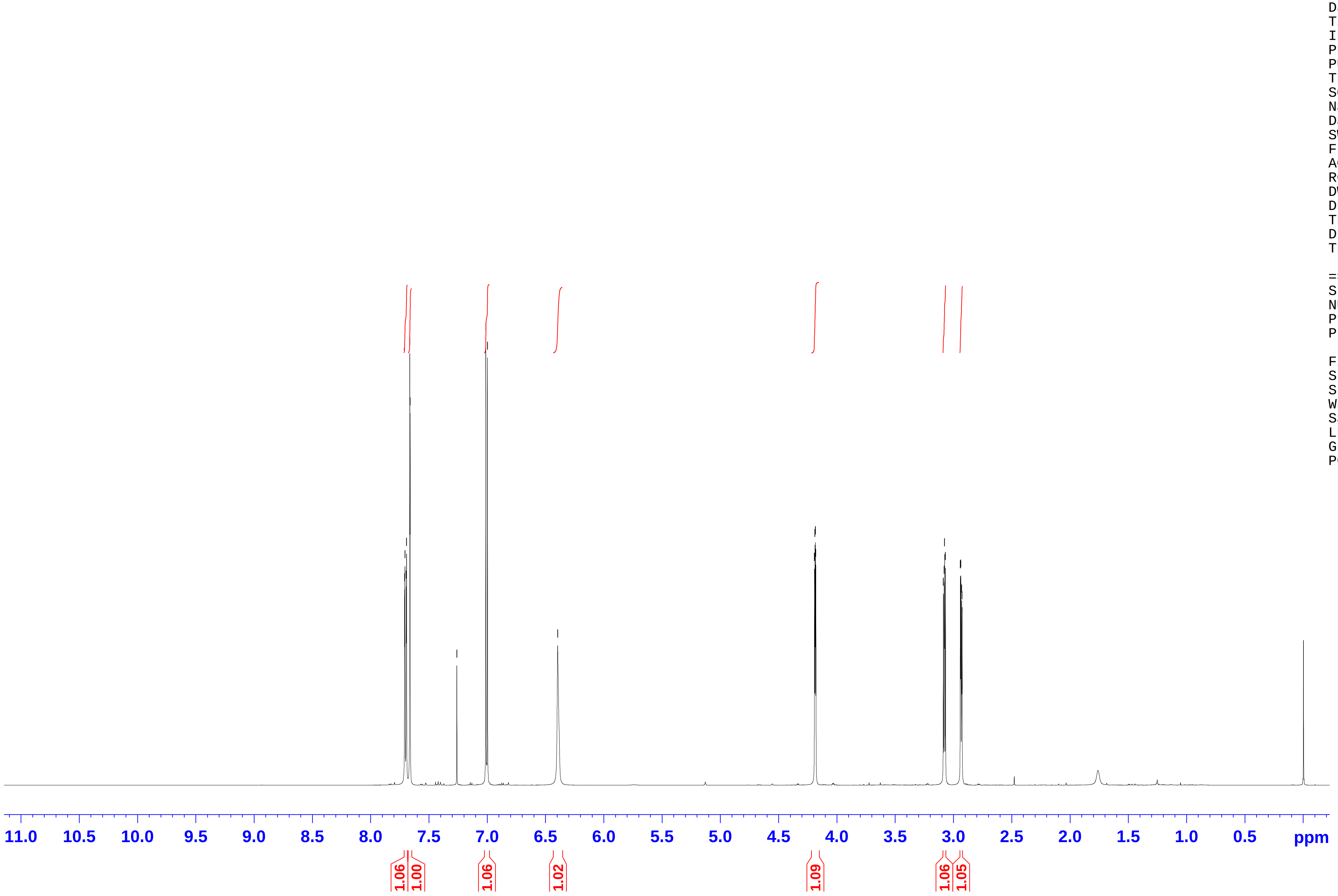

BRUKER

Current Data Parameters
NAME VB-451
EXPNO 20
PROCNO 1

F2 - Acquisition Parameters
Date_ 20191030
Time 21.18
INSTRUM spect
PROBHD 5 mm CPPBBO BB
PULPROG zg30
TD 65536
SOLVENT CDCl3
NS 16
DS 2
SWH 12019.230 Hz
FIDRES 0.183399 Hz
AQ 2.7262976 sec
RG 17.5
DW 41.600 usec
DE 10.00 usec
TE 290.2 K
D1 1.00000000 sec
TD0 1

======== CHANNEL f1 ========
SFO1 600.1337060 MHz
NUC1 1H
P1 12.00 usec
PLW1 21.00000000 W

F2 - Processing parameters
SI 65536
SF 600.1300151 MHz
WDW EM
SSB 0
LB 0.30 Hz
GB 0
PC 1.00

^{13}C NMR of 17 (150MHz, $CDCl_3$)

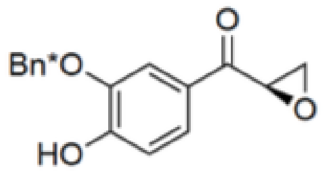

Bn* = d_7-benzyl

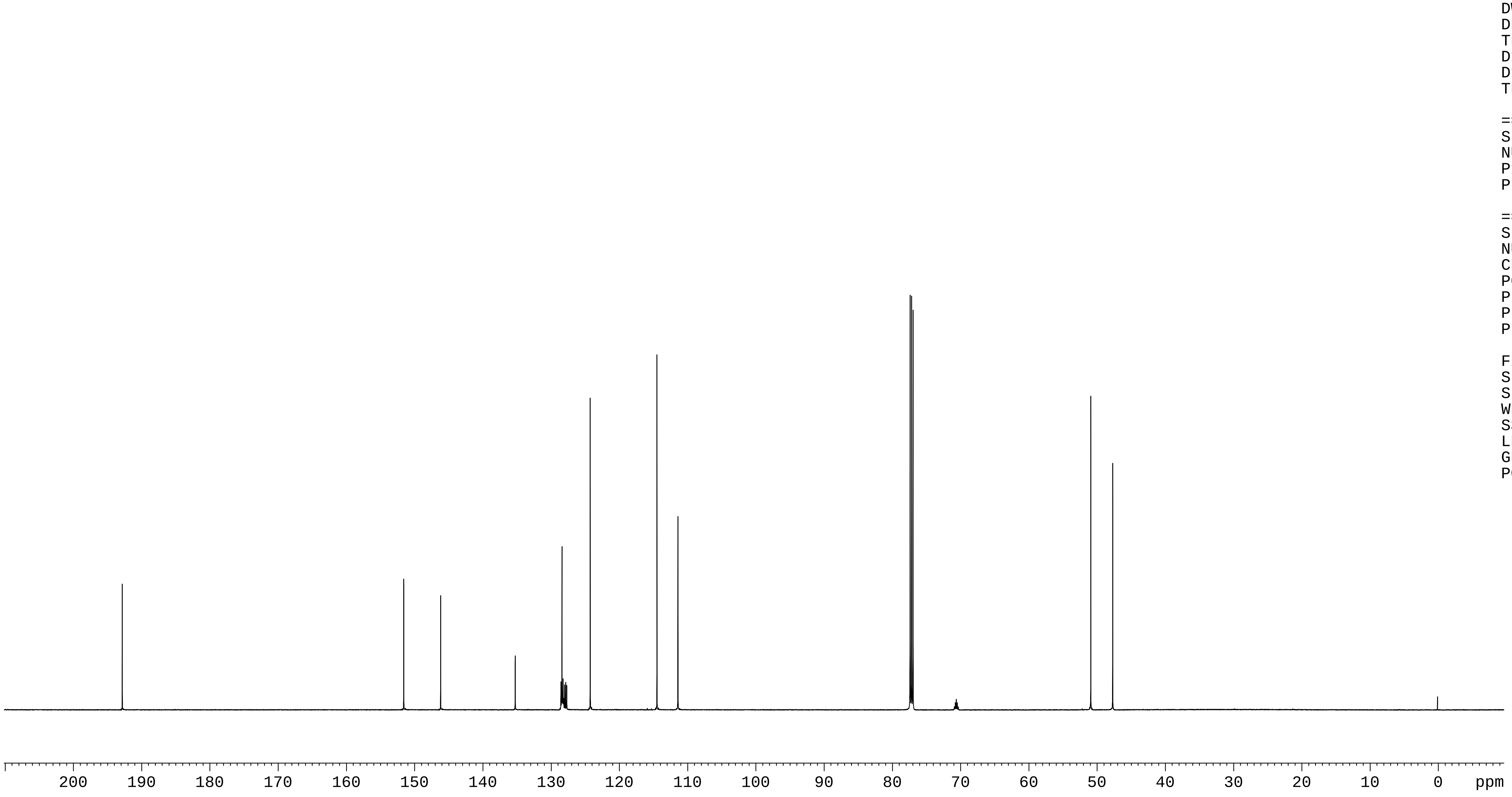


```
Current Data Parameters
NAME             VB-451
EXPNO                21
PROCNO                1

F2 - Acquisition Parameters
Date_          20191031
Time               7.46
INSTRUM           spect
PROBHD   5 mm CPPBBO BB
PULPROG         zgpg30
TD                65536
SOLVENT           CDCl3
NS                 1800
DS                    4
SWH           36057.691 Hz
FIDRES         0.550197 Hz
AQ            0.9087659 sec
RG               175.56
DW               13.867 usec
DE                18.00 usec
TE                289.3 K
D1           2.00000000 sec
D11          0.03000000 sec
TD0                   1

======== CHANNEL f1 ========
SFO1        150.9178981 MHz
NUC1                13C
P1                10.00 usec
PLW1        80.00000000 W

======== CHANNEL f2 ========
SFO2        600.1324005 MHz
NUC2                 1H
CPDPRG[2        waltz16
PCPD2             70.00 usec
PLW2        13.43999958 W
PLW12        0.61714000 W
PLW13        0.31042001 W

F2 - Processing parameters
SI                32768
SF          150.9027960 MHz
WDW                  EM
SSB      0
LB                 1.00 Hz
GB       0
PC                 1.40
```

^{1}H NMR of SI-5 (600MHz, $CDCl_3$)

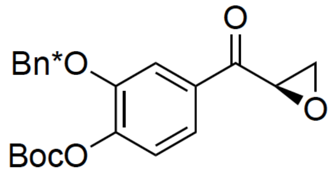

Bn* = d_7-benzyl

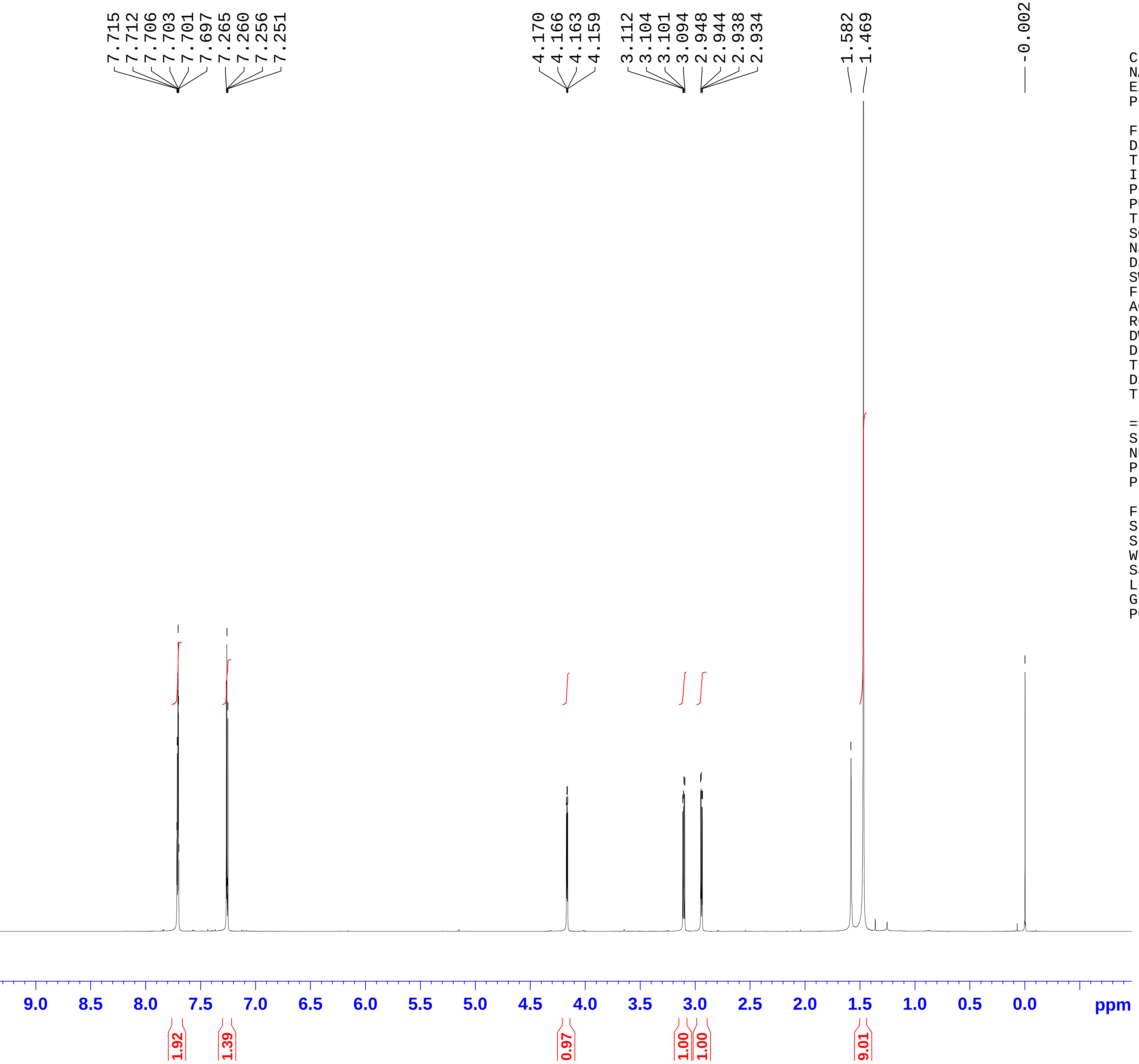

BRUKER

```
Current Data Parameters
NAME                VB-453
EXPNO                   40
PROCNO                   1

F2 - Acquisition Parameters
Date_             20211011
Time                 20.12
INSTRUM              spect
PROBHD    5 mm CPPBBO BB
PULPROG               zg30
TD                   65536
SOLVENT              CDCl3
NS                      16
DS                       2
SWH              12019.230 Hz
FIDRES            0.183399 Hz
AQ               2.7262976 sec
RG                   31.94
DW                  41.600 usec
DE                   10.00 usec
TE                   298.1 K
D1              1.00000000 sec
TD0                      1

======== CHANNEL f1 ========
SFO1           600.1337060 MHz
NUC1                    1H
P1                   12.00 usec
PLW1           21.00000000 W

F2 - Processing parameters
SI                   65536
SF             600.1300150 MHz
WDW                     EM
SSB      0
LB                    0.30 Hz
GB       0
PC                    1.00
```

^{13}C NMR of SI-5 (150MHz, $CDCl_3$)

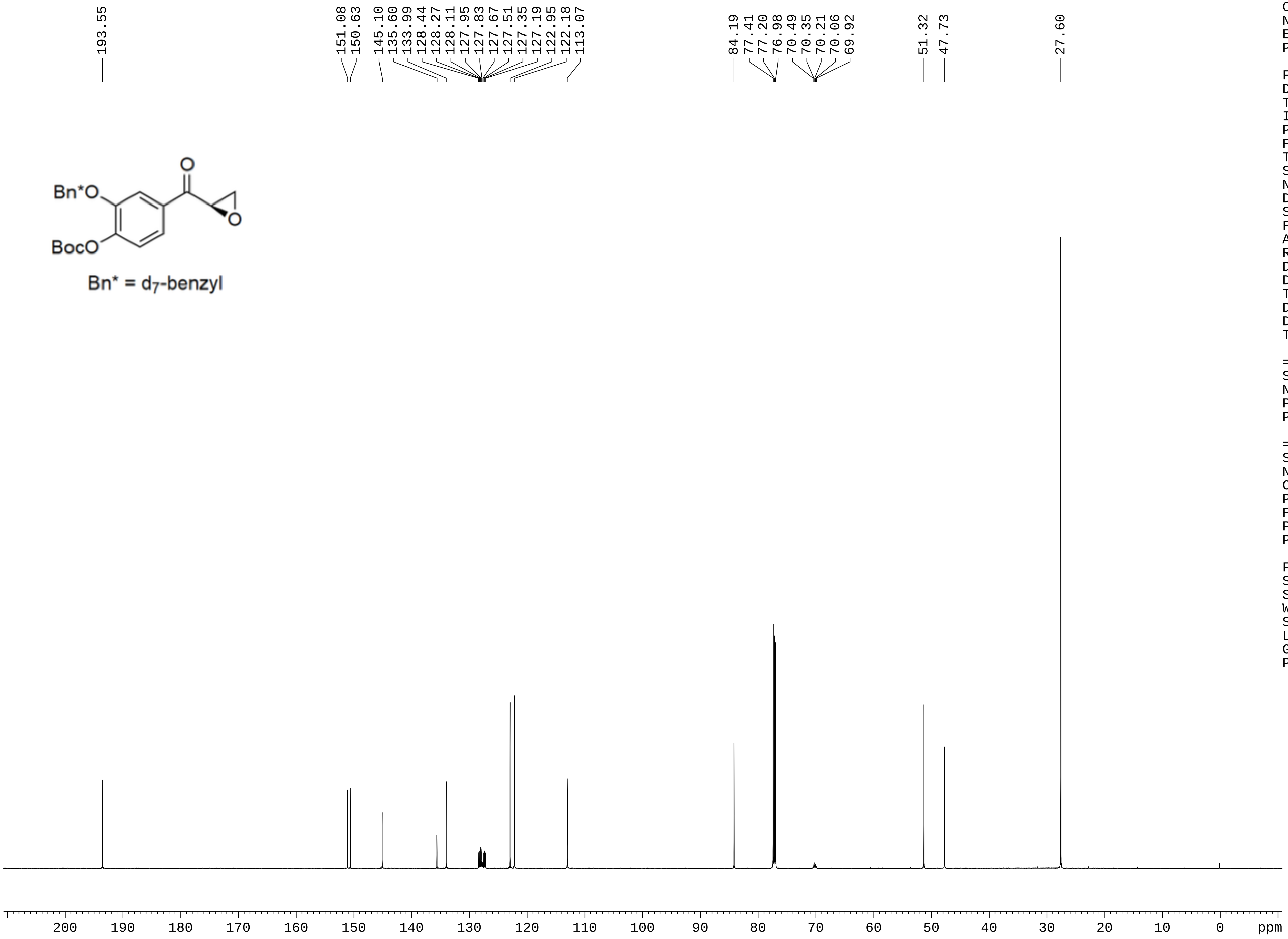


```
Current Data Parameters
NAME              VB-453
EXPNO                 21
PROCNO                 1

F2 - Acquisition Parameters
Date_           20191102
Time                0.58
INSTRUM            spect
PROBHD   5 mm CPPBBO BB
PULPROG          zgpg30
TD                65536
SOLVENT            CDCl3
NS                  1024
DS                     4
SWH           36057.691 Hz
FIDRES         0.550197 Hz
AQ            0.9087659 sec
RG               175.56
DW               13.867 usec
DE                18.00 usec
TE                290.1 K
D1           2.00000000 sec
D11          0.03000000 sec
TD0                    1

======== CHANNEL f1 ========
SFO1        150.9178981 MHz
NUC1                 13C
P1                10.00 usec
PLW1        80.00000000 W

======== CHANNEL f2 ========
SFO2        600.1324005 MHz
NUC2                  1H
CPDPRG[2         waltz16
PCPD2             70.00 usec
PLW2        13.43999958 W
PLW12        0.61714000 W
PLW13        0.31042001 W

F2 - Processing parameters
SI                 32768
SF          150.9027974 MHz
WDW                   EM
SSB      0
LB                 1.00 Hz
GB       0
PC                  1.40
```

^{1}H NMR of *anti*-18 (600MHz, $CDCl_3$)

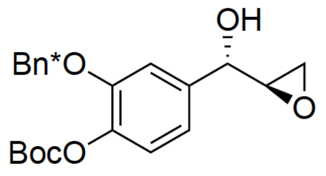

Bn* = d_7-benzyl

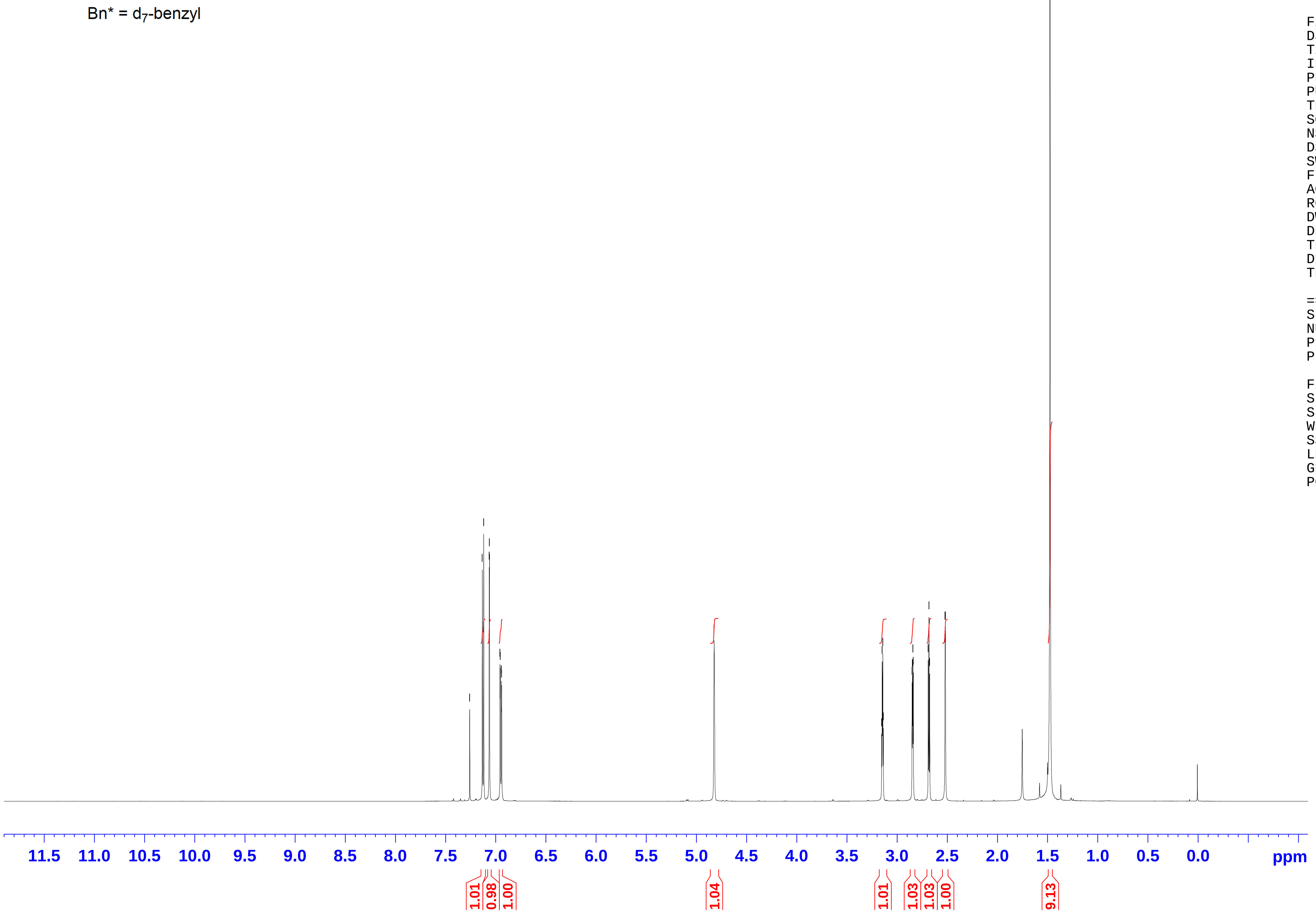


```
Current Data Parameters
NAME              VB-454
EXPNO                 40
PROCNO                 1

F2 - Acquisition Parameters
Date_           20211011
Time               18.02
INSTRUM            spect
PROBHD    5 mm CPPBBO BB
PULPROG             zg30
TD                 65536
SOLVENT            CDCl3
NS                    16
DS                     2
SWH            12019.230 Hz
FIDRES          0.183399 Hz
AQ             2.7262976 sec
RG                 15.79
DW                41.600 usec
DE                 10.00 usec
TE                 298.2 K
D1            1.00000000 sec
TD0                    1

======== CHANNEL f1 ========
SFO1         600.1337060 MHz
NUC1                  1H
P1                 12.00 usec
PLW1         21.00000000 W

F2 - Processing parameters
SI                 65536
SF           600.1300151 MHz
WDW                   EM
SSB      0
LB                  0.30 Hz
GB       0
PC                  1.00
```

^{13}C NMR of *anti*-18 (150MHz, $CDCl_3$)

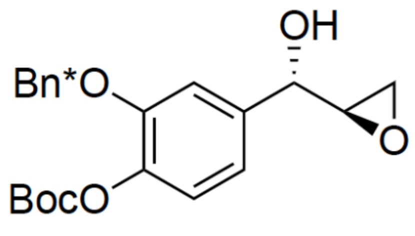

Bn* = d_7-benzyl

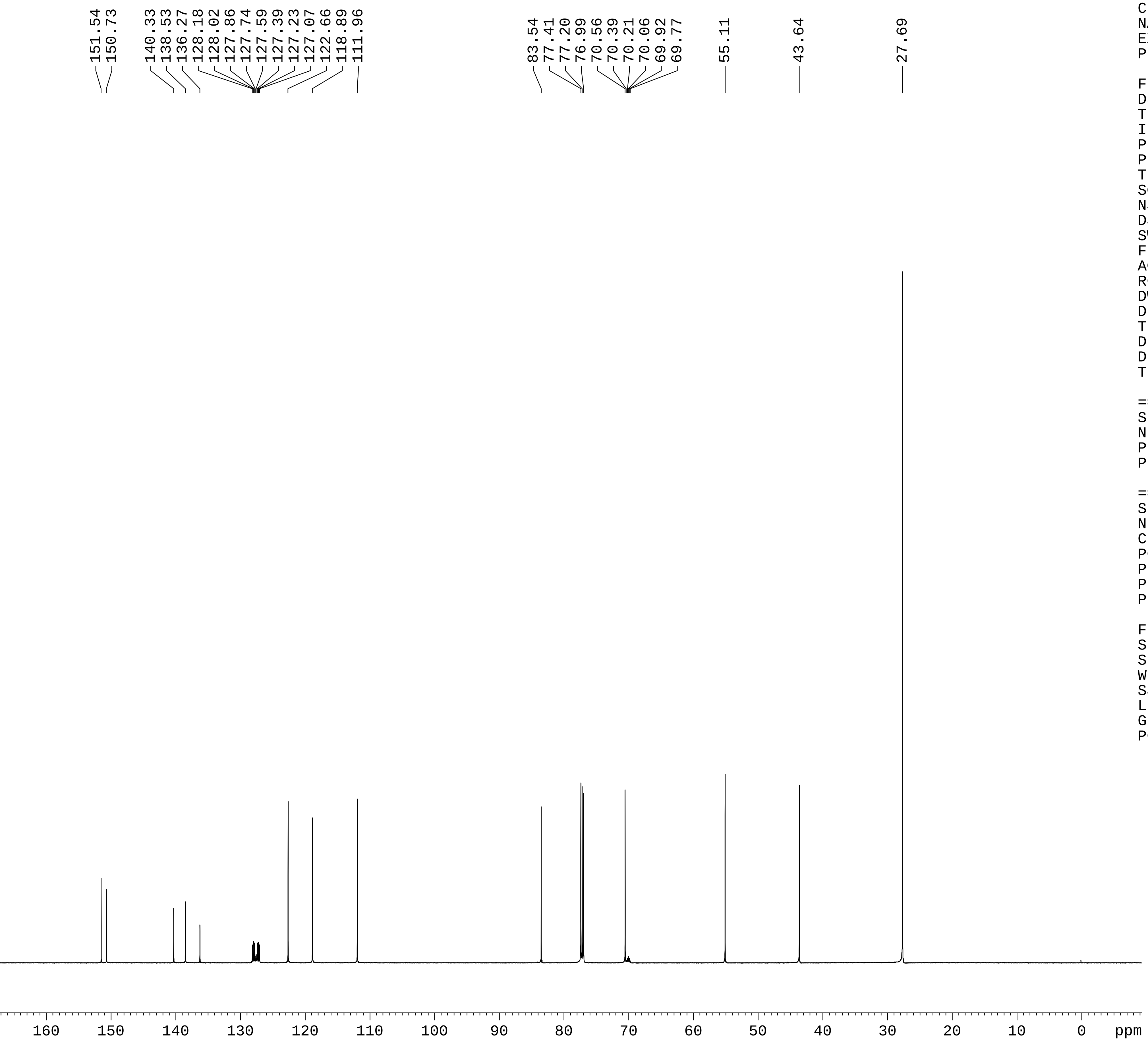


```
Current Data Parameters
NAME              VB-454
EXPNO                 50
PROCNO                 1

F2 - Acquisition Parameters
Date_           20211012
Time               22.55
INSTRUM            spect
PROBHD   5 mm CPPBBO BB
PULPROG          zgpg30
TD                 65536
SOLVENT            CDCl3
NS                  1024
DS                     4
SWH           36057.691 Hz
FIDRES         0.550197 Hz
AQ            0.9087659 sec
RG                175.56
DW                13.867 usec
DE                 18.00 usec
TE                 298.1 K
D1            2.00000000 sec
D11           0.03000000 sec
TD0                    1

======== CHANNEL f1 ========
SFO1        150.9178981 MHz
NUC1                 13C
P1                 10.00 usec
PLW1         80.00000000 W

======== CHANNEL f2 ========
SFO2        600.1324005 MHz
NUC2                  1H
CPDPRG[2         waltz16
PCPD2              70.00 usec
PLW2         13.43999958 W
PLW12         0.61714000 W
PLW13         0.31042001 W

F2 - Processing parameters
SI                 32768
SF          150.9027948 MHz
WDW                   EM
SSB      0
LB                  1.00 Hz
GB       0
PC                  1.40
```

^{1}H NMR of 19 (600MHz, $CDCl_3$)

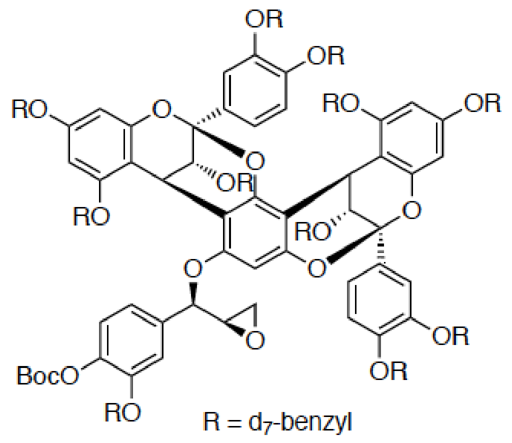

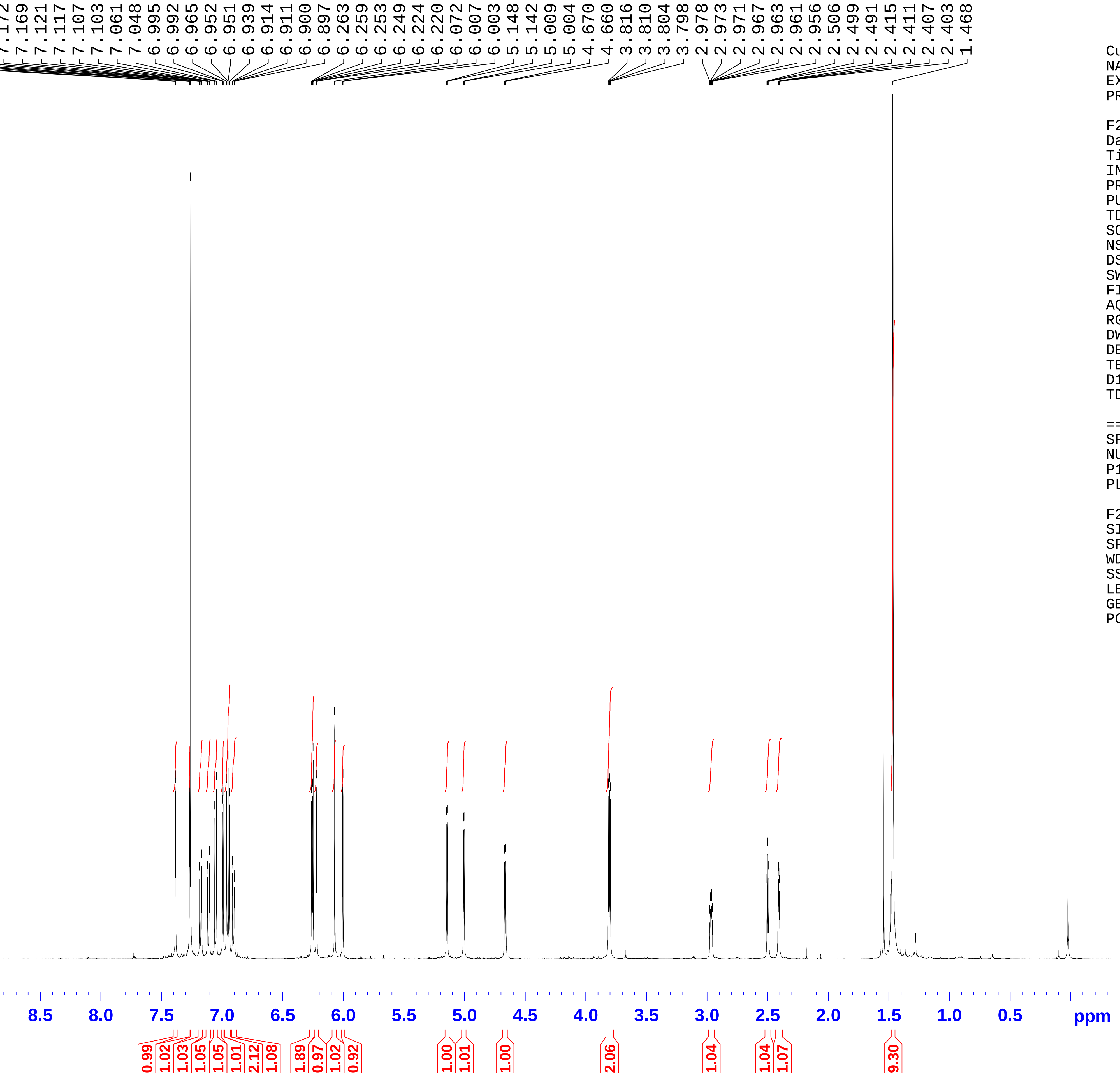


```
Current Data Parameters
NAME              VB-728-1
EXPNO                   30
PROCNO                   1

F2 - Acquisition Parameters
Date_             20201215
Time                 22.08
INSTRUM              spect
PROBHD   5 mm CPPBBO BB
PULPROG               zg30
TD                   65536
SOLVENT              CDCl3
NS                      16
DS                       2
SWH              12019.230 Hz
FIDRES            0.183399 Hz
AQ               2.7262976 sec
RG                   31.94
DW                  41.600 usec
DE                   10.00 usec
TE                   298.2 K
D1              1.00000000 sec
TD0                      1

======== CHANNEL f1 ========
SFO1         600.1337060 MHz
NUC1                    1H
P1                   12.00 usec
PLW1        21.00000000 W

F2 - Processing parameters
SI                   65536
SF           600.1300147 MHz
WDW                     EM
SSB      0
LB                    0.30 Hz
GB       0
PC                    1.00
```

^{13}C NMR of 19 (150MHz, $CDCl_3$)

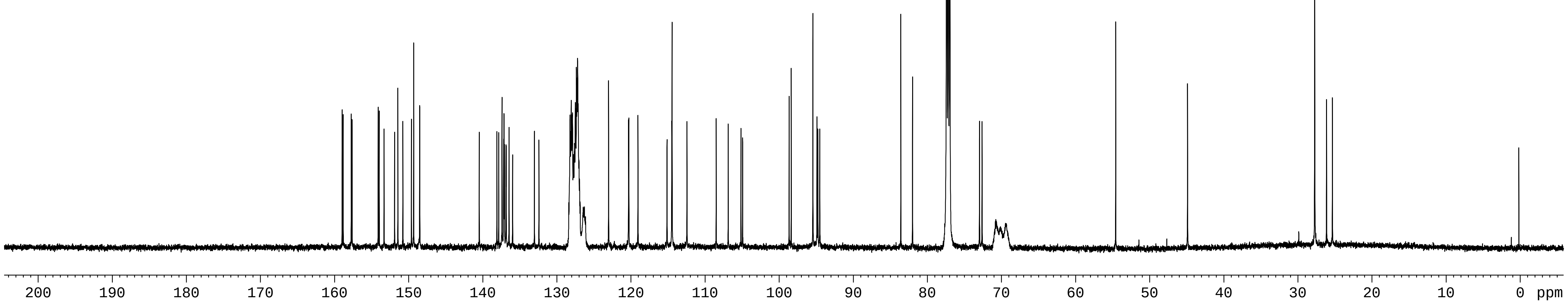

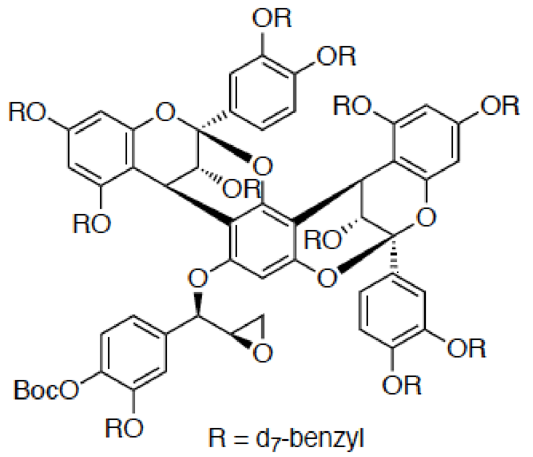

Current Data Parameters

NAME	VB-728-1
EXPNO	31
PROCNO	1

F2 - Acquisition Parameters

Date_	20201216
Time	1.30
INSTRUM	spect
PROBHD	5 mm CPPBBO BB
PULPROG	zgpg30
TD	65536
SOLVENT	CDCl3
NS	4000
DS	4
SWH	36057.691 Hz
FIDRES	0.550197 Hz
AQ	0.9087659 sec
RG	175.56
DW	13.867 usec
DE	18.00 usec
TE	298.0 K
D1	2.00000000 sec
D11	0.03000000 sec
TD0	1

======== CHANNEL f1 ========

SFO1	150.9178981 MHz
NUC1	13C
P1	10.00 usec
PLW1	80.00000000 W

======== CHANNEL f2 ========

SFO2	600.1324005 MHz
NUC2	1H
CPDPRG[2	waltz16
PCPD2	70.00 usec
PLW2	13.43999958 W
PLW12	0.61714000 W
PLW13	0.31042001 W

F2 - Processing parameters

SI	32768
SF	150.9027874 MHz
WDW	EM
SSB	0
LB	1.00 Hz
GB	0
PC	1.40

^{1}H NMR of 20 (600HMz, $CDCl_3$)

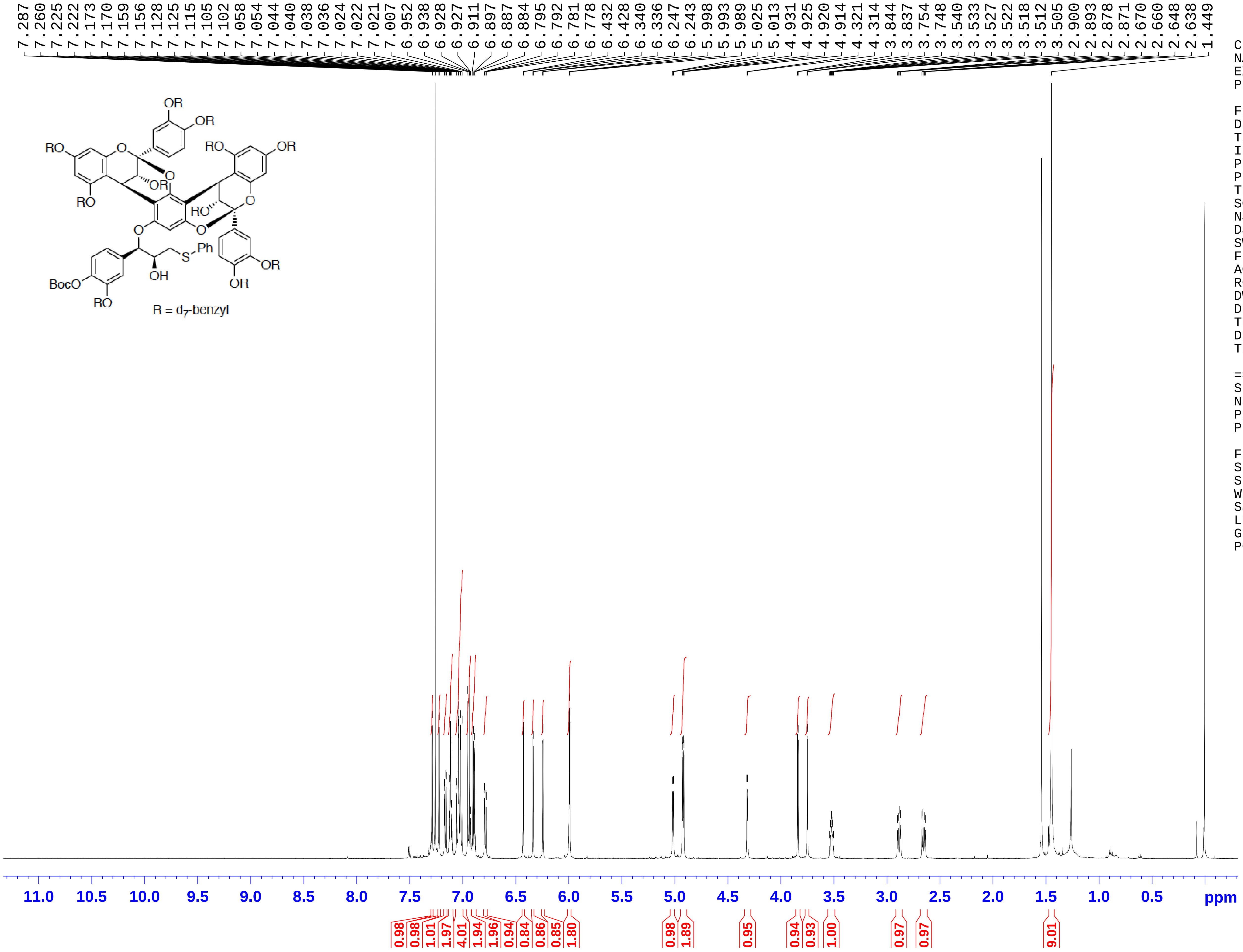

BRUKER

Current Data Parameters
NAME VB-735
EXPNO 12
PROCNO 1

F2 - Acquisition Parameters
Date_ 20210104
Time 18.06
INSTRUM spect
PROBHD 5 mm CPPBBO BB
PULPROG zg30
TD 65536
SOLVENT CDCl3
NS 100
DS 2
SWH 12019.230 Hz
FIDRES 0.183399 Hz
AQ 2.7262976 sec
RG 31.94
DW 41.600 usec
DE 10.00 usec
TE 298.2 K
D1 1.00000000 sec
TD0 1

======== CHANNEL f1 ========
SFO1 600.1337060 MHz
NUC1 1H
P1 12.00 usec
PLW1 21.00000000 W

F2 - Processing parameters
SI 65536
SF 600.1300147 MHz
WDW EM
SSB 0
LB 0.30 Hz
GB 0
PC 1.00

^{13}C NMR of 20 (150MHz, $CDCl_3$)

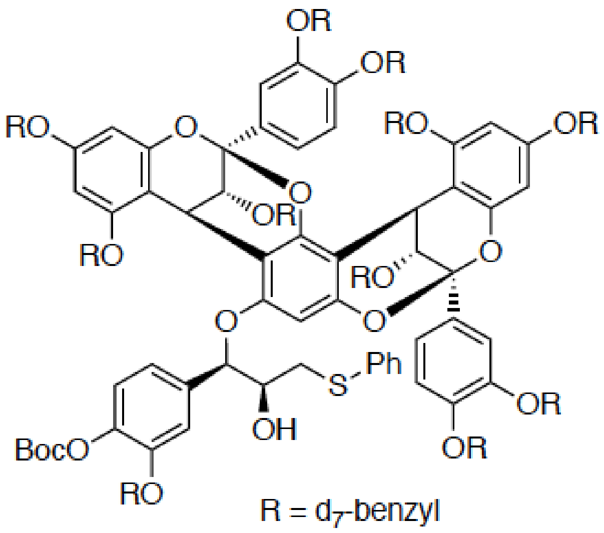

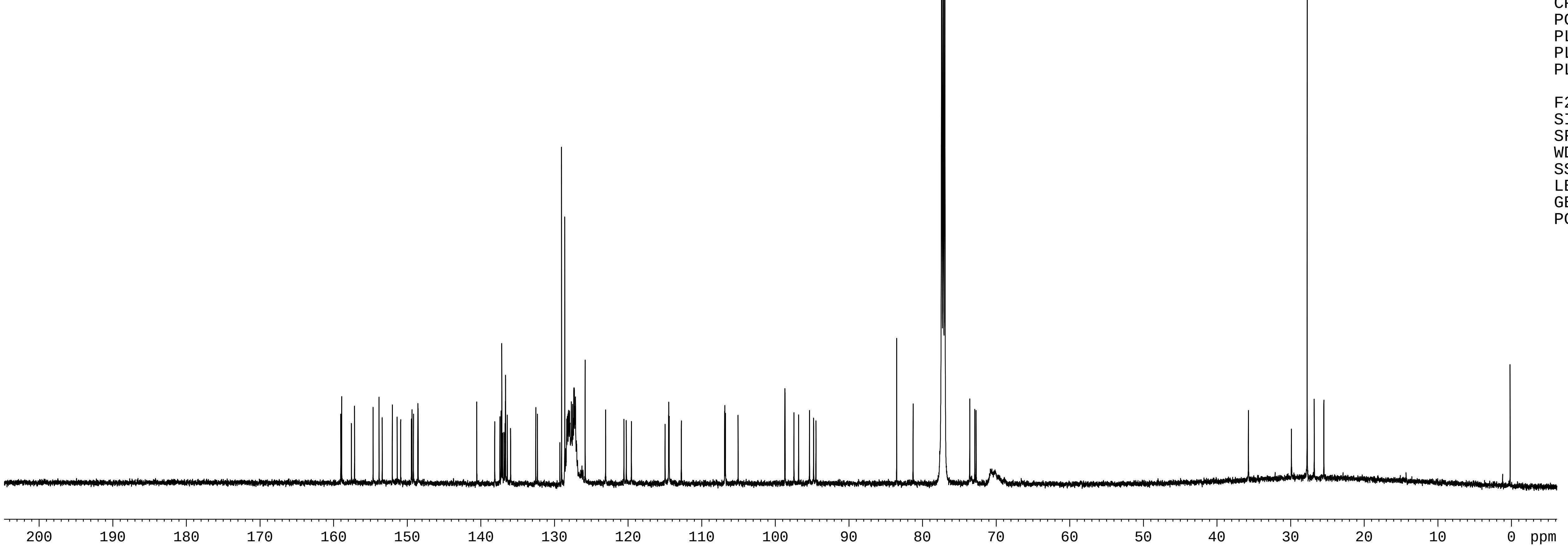

Current Data Parameters
NAME VB-735
EXPNO 13
PROCNO 1

F2 - Acquisition Parameters
Date_ 20210106
Time 4.38
INSTRUM spect
PROBHD 5 mm CPPBBO BB
PULPROG zgpg30
TD 65536
SOLVENT CDCl3
NS 8000
DS 4
SWH 36057.691 Hz
FIDRES 0.550197 Hz
AQ 0.9087659 sec
RG 175.56
DW 13.867 usec
DE 18.00 usec
TE 298.2 K
D1 2.00000000 sec
D11 0.03000000 sec
TD0 1

======== CHANNEL f1 ========
SFO1 150.9178981 MHz
NUC1 13C
P1 10.00 usec
PLW1 80.00000000 W

======== CHANNEL f2 ========
SFO2 600.1324005 MHz
NUC2 1H
CPDPRG[2 waltz16
PCPD2 70.00 usec
PLW2 13.43999958 W
PLW12 0.61714000 W
PLW13 0.31042001 W

F2 - Processing parameters
SI 32768
SF 150.9027829 MHz
WDW EM
SSB 0
LB 1.00 Hz
GB 0
PC 1.40

^{1}H NMR of *syn*-21 (600MHz, $CDCl_3$)

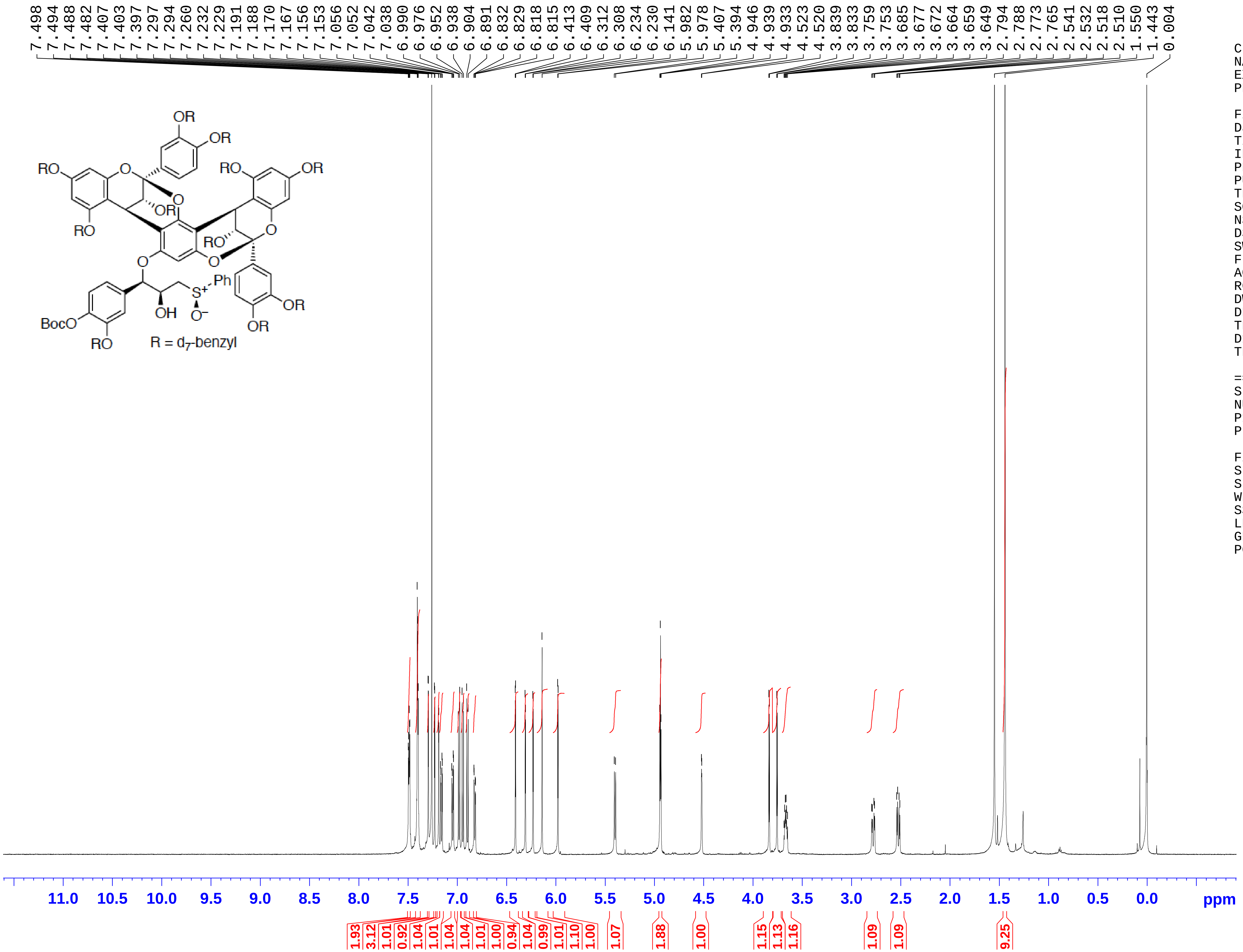


```
Current Data Parameters
NAME            VB-2desired
EXPNO                    30
PROCNO                    1

F2 - Acquisition Parameters
Date_              20201202
Time                  19.43
INSTRUM               spect
PROBHD   5 mm CPPBBO BB
PULPROG                zg30
TD                    65536
SOLVENT               CDCl3
NS                       16
DS                        2
SWH               12019.230 Hz
FIDRES             0.183399 Hz
AQ                2.7262976 sec
RG                    31.94
DW                   41.600 usec
DE                    10.00 usec
TE                    298.2 K
D1               1.00000000 sec
TD0                       1

======== CHANNEL f1 ========
SFO1         600.1337060 MHz
NUC1                     1H
P1                    12.00 usec
PLW1           21.00000000 W

F2 - Processing parameters
SI                    65536
SF           600.1300150 MHz
WDW                      EM
SSB      0
LB                     0.30 Hz
GB       0
PC                     1.00
```

^{13}C NMR of *syn*-21 (150MHz, $CDCl_3$)

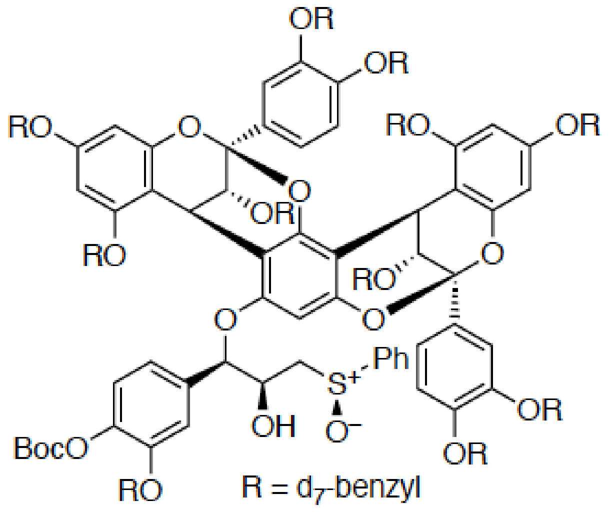

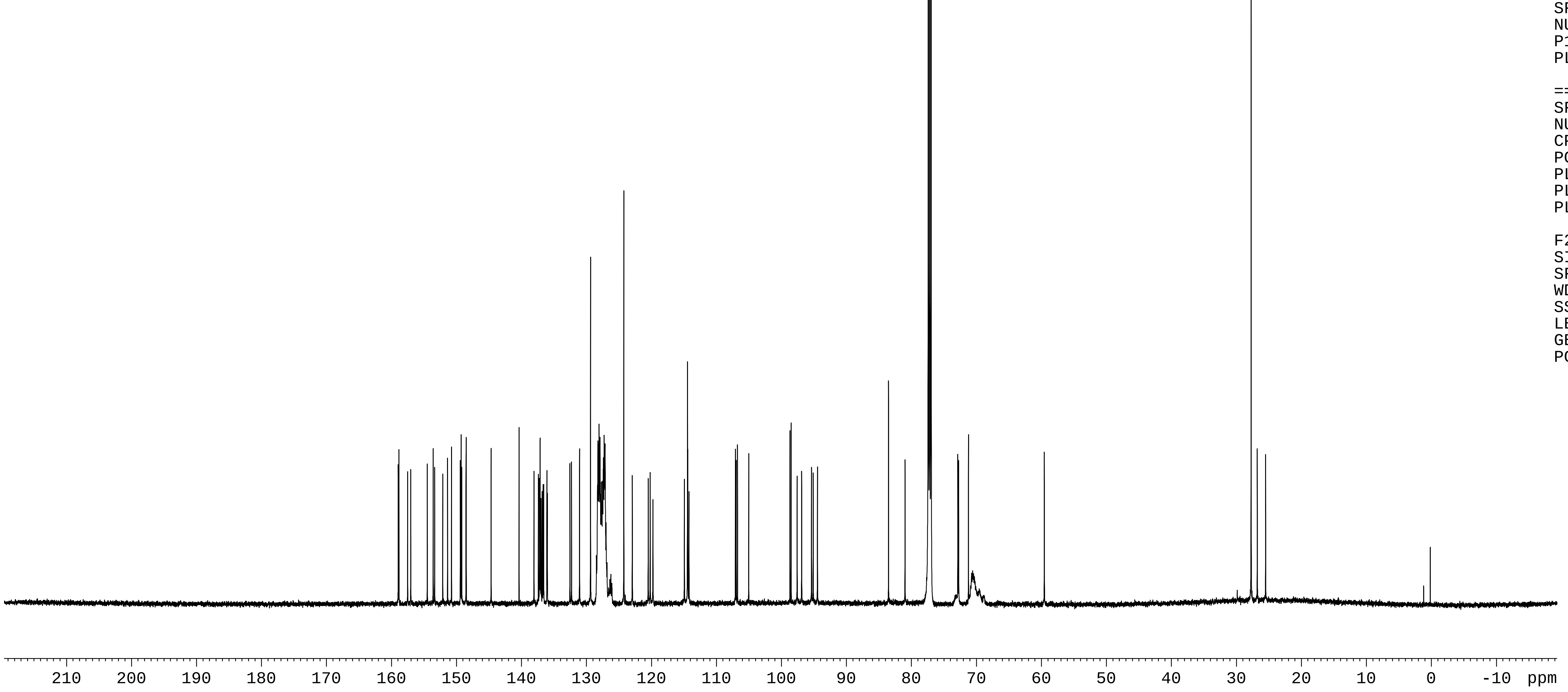

BRUKER

```
Current Data Parameters
NAME          VB-2desired
EXPNO                  21
PROCNO                  1

F2 - Acquisition Parameters
Date_            20201201
Time                 7.51
INSTRUM             spect
PROBHD   5 mm CPPBBO BB
PULPROG           zgpg30
TD                 65536
SOLVENT            CDCl3
NS                  5000
DS                     4
SWH            36057.691 Hz
FIDRES          0.550197 Hz
AQ             0.9087659 sec
RG                175.56
DW                13.867 usec
DE                 18.00 usec
TE                 298.2 K
D1            2.00000000 sec
D11           0.03000000 sec
TD0                    1

======== CHANNEL f1 ========
SFO1         150.9178981 MHz
NUC1                 13C
P1                 10.00 usec
PLW1         80.00000000 W

======== CHANNEL f2 ========
SFO2         600.1324005 MHz
NUC2                  1H
CPDPRG[2         waltz16
PCPD2              70.00 usec
PLW2         13.43999958 W
PLW12         0.61714000 W
PLW13         0.31042001 W

F2 - Processing parameters
SI                 32768
SF           150.9027880 MHz
WDW                   EM
SSB      0
LB                  1.00 Hz
GB       0
PC                  1.40
```

^{1}H NMR of *anti*-21 (600MHz, $CDCl_3$)

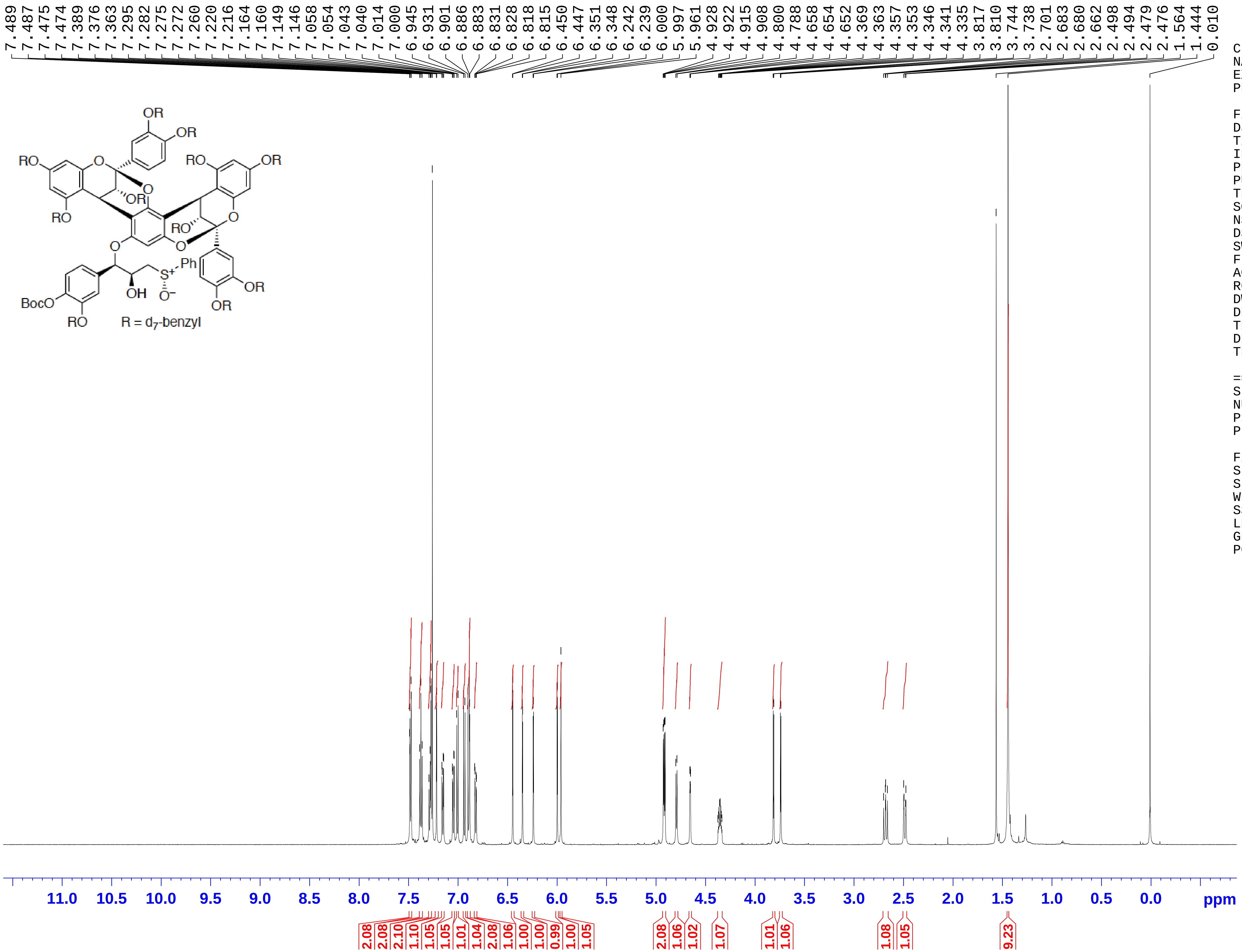


```
Current Data Parameters
NAME        VB-1undesired
EXPNO                  50
PROCNO                  1

F2 - Acquisition Parameters
Date_            20210115
Time                19.27
INSTRUM             spect
PROBHD    5 mm CPPBBO BB
PULPROG              zg30
TD                  65536
SOLVENT             CDCl3
NS                     16
DS                      2
SWH            12019.230 Hz
FIDRES          0.183399 Hz
AQ             2.7262976 sec
RG                   17.5
DW                 41.600 usec
DE                  10.00 usec
TE                  298.2 K
D1             1.00000000 sec
TD0                     1

======== CHANNEL f1 ========
SFO1        600.1337060 MHz
NUC1                     1H
P1                    12.00 usec
PLW1           21.00000000 W

F2 - Processing parameters
SI                  65536
SF            600.1300148 MHz
WDW                    EM
SSB      0
LB                   0.30 Hz
GB       0
PC                   1.00
```

^{13}C NMR of *anti*-21 (150MHz, $CDCl_3$)

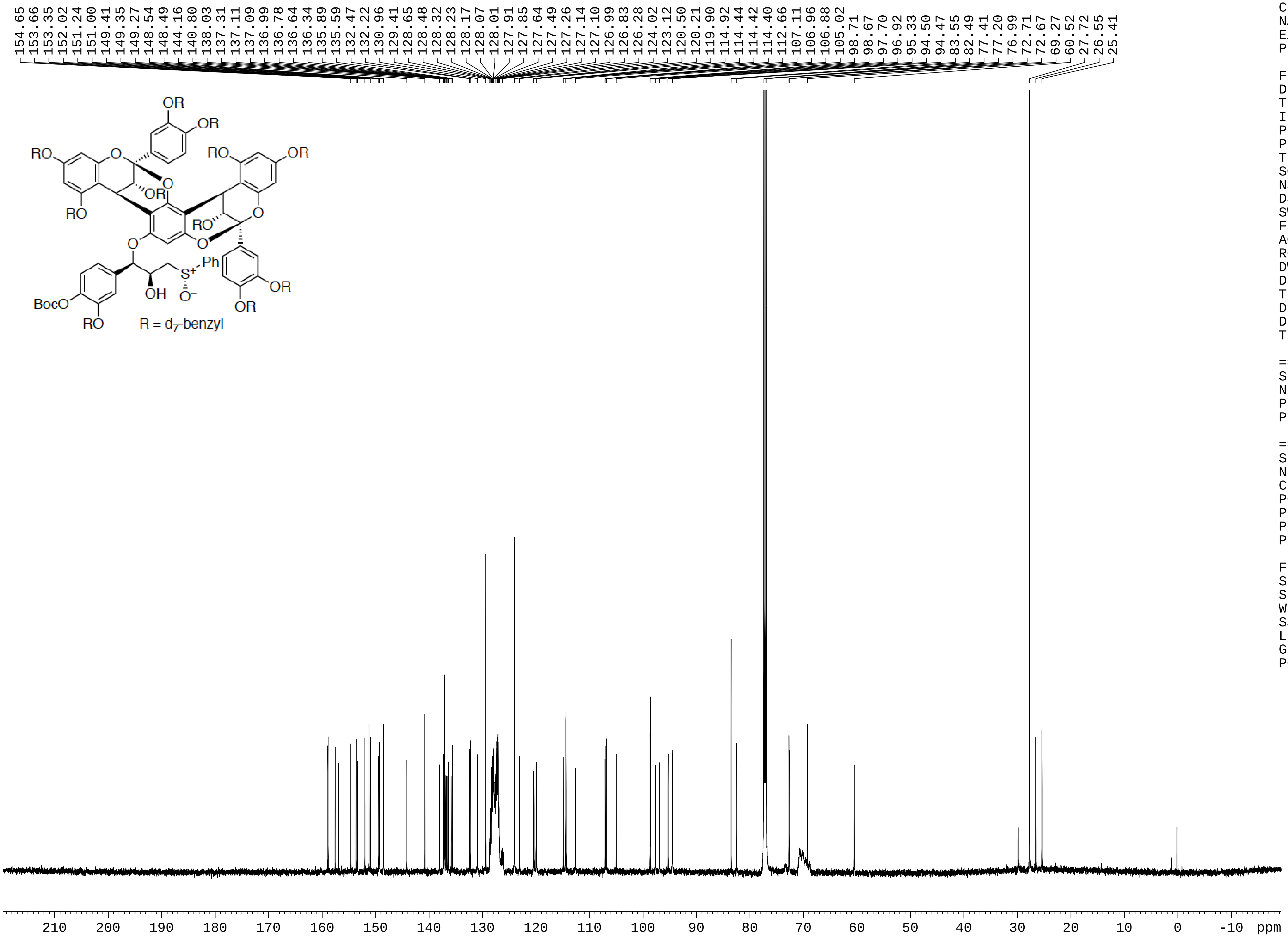


```
Current Data Parameters
NAME          VB-1undesired
EXPNO                    22
PROCNO                    1

F2 - Acquisition Parameters
Date_              20201201
Time                   3.41
INSTRUM               spect
PROBHD    5 mm CPPBBO BB
PULPROG             zgpg30
TD                    65536
SOLVENT               CDCl3
NS                     2000
DS                        4
SWH              36057.691 Hz
FIDRES            0.550197 Hz
AQ               0.9087659 sec
RG                   175.56
DW                   13.867 usec
DE                    18.00 usec
TE                    298.2 K
D1               2.00000000 sec
D11              0.03000000 sec
TD0                       1

======== CHANNEL f1 ========
SFO1           150.9178981 MHz
NUC1                    13C
P1                    10.00 usec
PLW1            80.00000000 W

======== CHANNEL f2 ========
SFO2           600.1324005 MHz
NUC2                     1H
CPDPRG[2            waltz16
PCPD2                 70.00 usec
PLW2            13.43999958 W
PLW12            0.61714000 W
PLW13            0.31042001 W

F2 - Processing parameters
SI                    32768
SF             150.9027884 MHz
WDW                      EM
SSB      0
LB                     1.00 Hz
GB       0
PC                     1.40
```

^{1}H NMR of 23 (600MHz, C_6D_6)

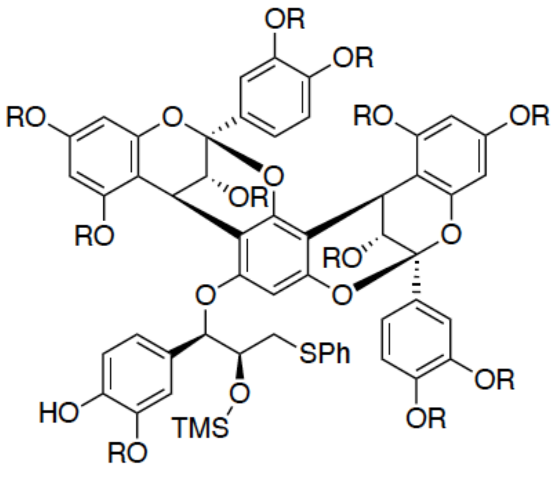

R = d_7-benzyl

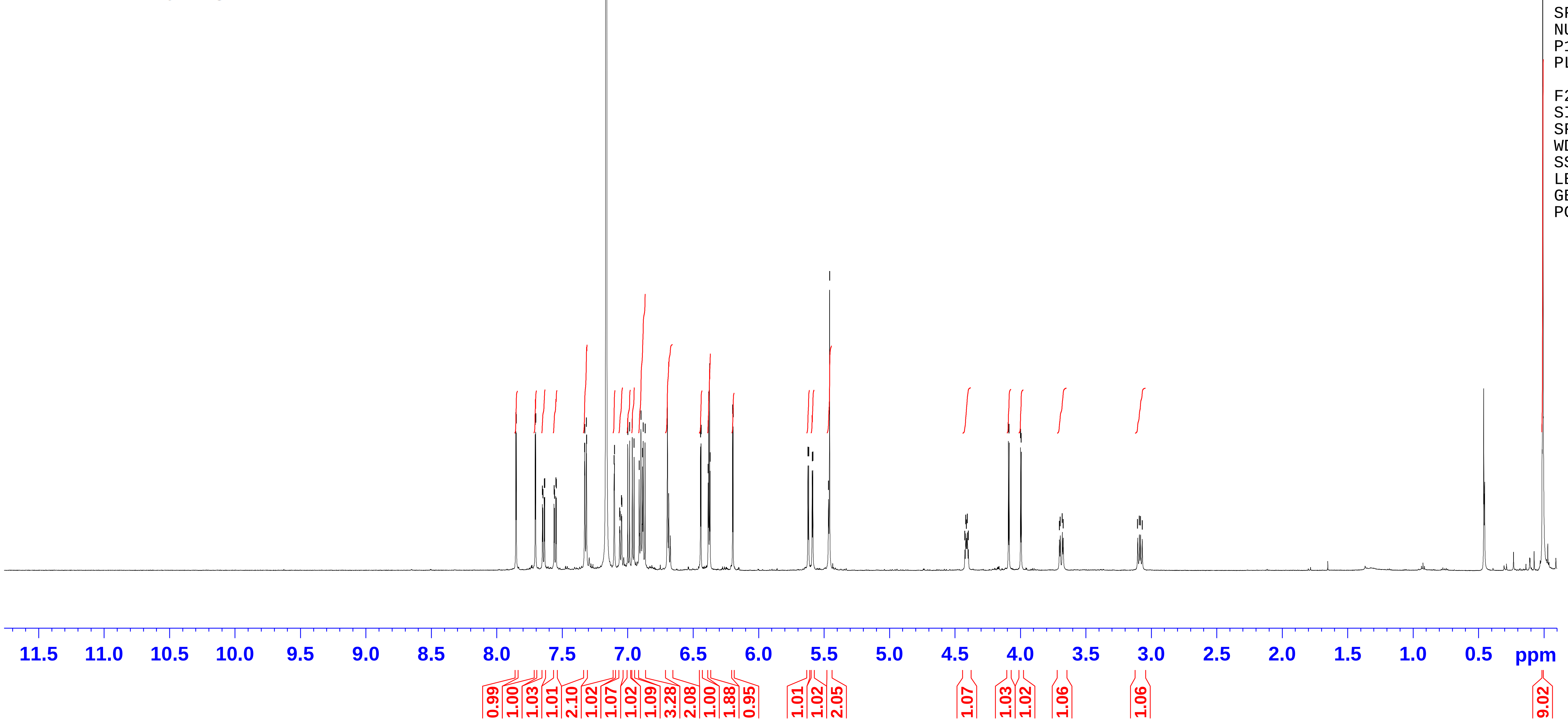

BRUKER

```
Current Data Parameters
NAME              VB-891-1B
EXPNO                    13
PROCNO                    1

F2 - Acquisition Parameters
Date_              20211025
Time                  17.10
INSTRUM               spect
PROBHD     5 mm CPPBBO BB
PULPROG                zg30
TD                    65536
SOLVENT                C6D6
NS                        2
DS                        2
SWH               12019.230 Hz
FIDRES             0.183399 Hz
AQ                2.7262976 sec
RG                    31.94
DW                   41.600 usec
DE                    10.00 usec
TE                    298.1 K
D1               1.00000000 sec
TD0                       1

======== CHANNEL f1 ========
SFO1         600.1337060 MHz
NUC1                     1H
P1                    12.00 usec
PLW1            21.00000000 W

F2 - Processing parameters
SI                    65536
SF           600.1299925 MHz
WDW                      EM
SSB      0
LB                     0.30 Hz
GB       0
PC                     1.00
```

^{13}C NMR of 23 (150MHz, C_6D_6)

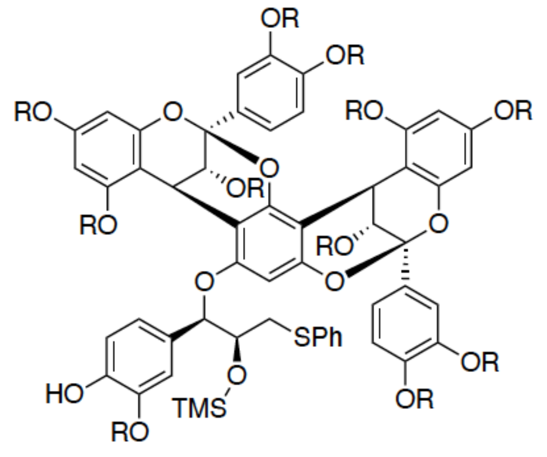

R = d_7-benzyl

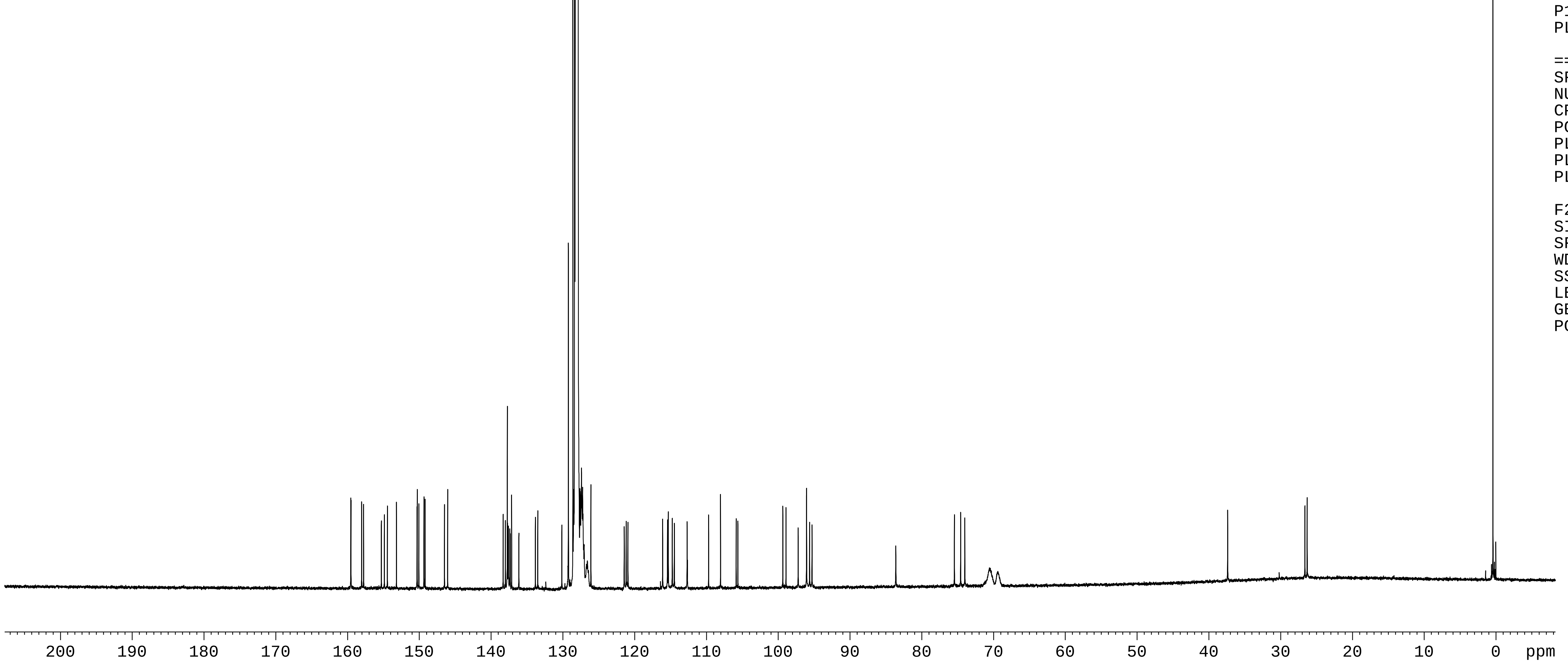


```
Current Data Parameters
NAME             VB-891-1B
EXPNO                   15
PROCNO                   1

F2 - Acquisition Parameters
Date_             20211026
Time                  5.18
INSTRUM              spect
PROBHD   5 mm CPPBBO BB
PULPROG            zgpg30
TD                  65536
SOLVENT              C6D6
NS                   8500
DS                      4
SWH             36057.691 Hz
FIDRES           0.550197 Hz
AQ              0.9087659 sec
RG                 175.56
DW                 13.867 usec
DE                  18.00 usec
TE                  298.2 K
D1             2.00000000 sec
D11            0.03000000 sec
TD0                     1

======== CHANNEL f1 ========
SFO1         150.9178981 MHz
NUC1                   13C
P1                   10.00 usec
PLW1           80.00000000 W

======== CHANNEL f2 ========
SFO2         600.1324005 MHz
NUC2                    1H
CPDPRG[2           waltz16
PCPD2                70.00 usec
PLW2           13.43999958 W
PLW12           0.61714000 W
PLW13           0.31042001 W

F2 - Processing parameters
SI                   32768
SF            150.9027539 MHz
WDW                     EM
SSB      0
LB                    1.00 Hz
GB       0
PC                    1.40
```

^{1}H NMR of 22 (600MHz, C_6D_6)

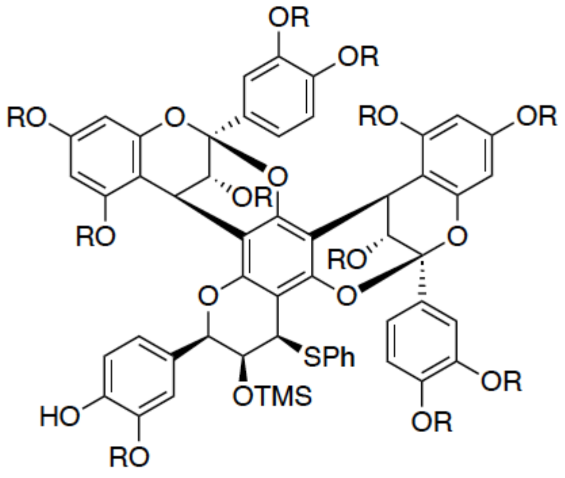

R = d_7-benzyl

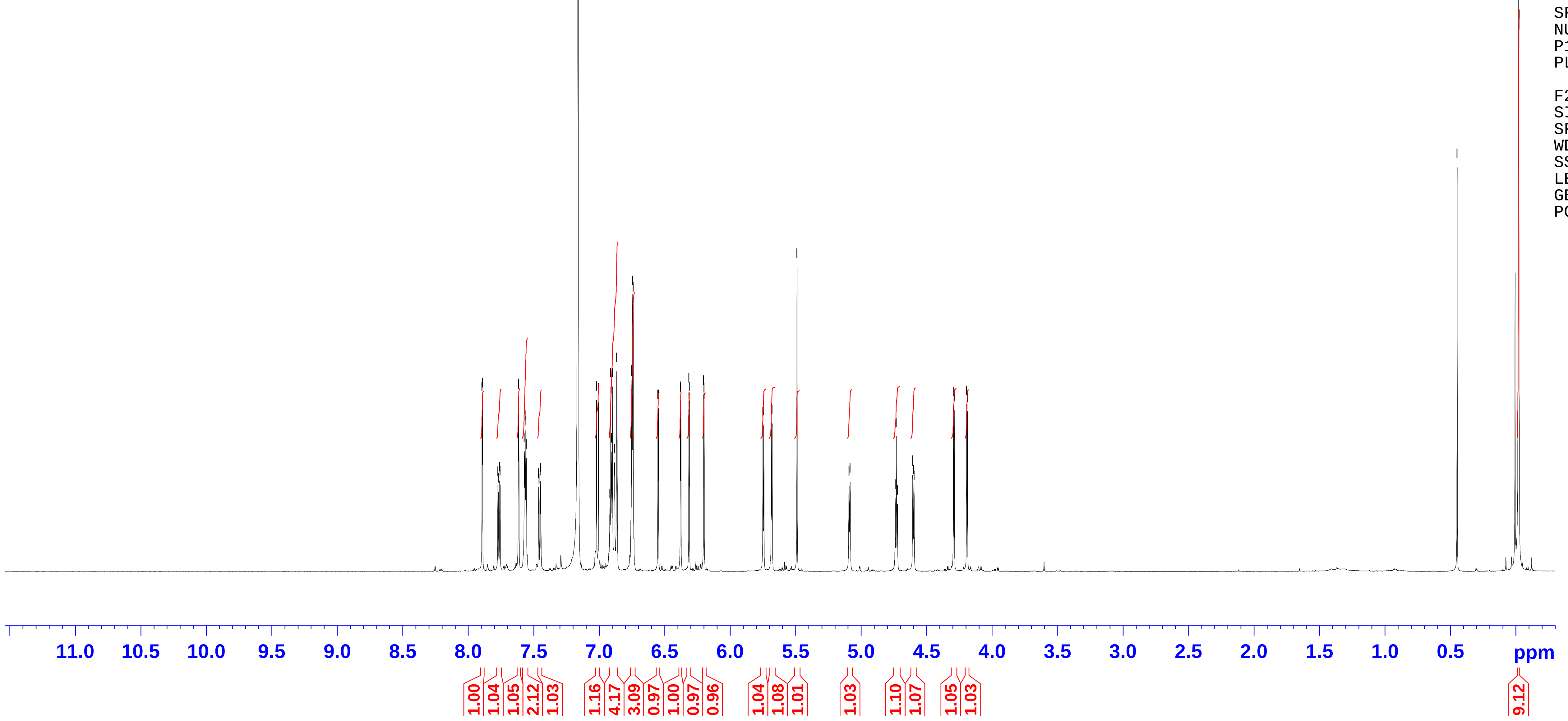

BRUKER

Current Data Parameters
NAME VB-718
EXPNO 90
PROCNO 1

F2 - Acquisition Parameters
Date_ 20211018
Time 19.48
INSTRUM spect
PROBHD 5 mm CPPBBO BB
PULPROG zg30
TD 65536
SOLVENT C6D6
NS 16
DS 2
SWH 12019.230 Hz
FIDRES 0.183399 Hz
AQ 2.7262976 sec
RG 28.28
DW 41.600 usec
DE 10.00 usec
TE 298.1 K
D1 1.00000000 sec
TD0 1

======== CHANNEL f1 ========
SFO1 600.1337060 MHz
NUC1 1H
P1 12.00 usec
PLW1 21.00000000 W

F2 - Processing parameters
SI 65536
SF 600.1299927 MHz
WDW EM
SSB 0
LB 0.30 Hz
GB 0
PC 1.00

^{13}C NMR of 22 (150MHz, C_6D_6)

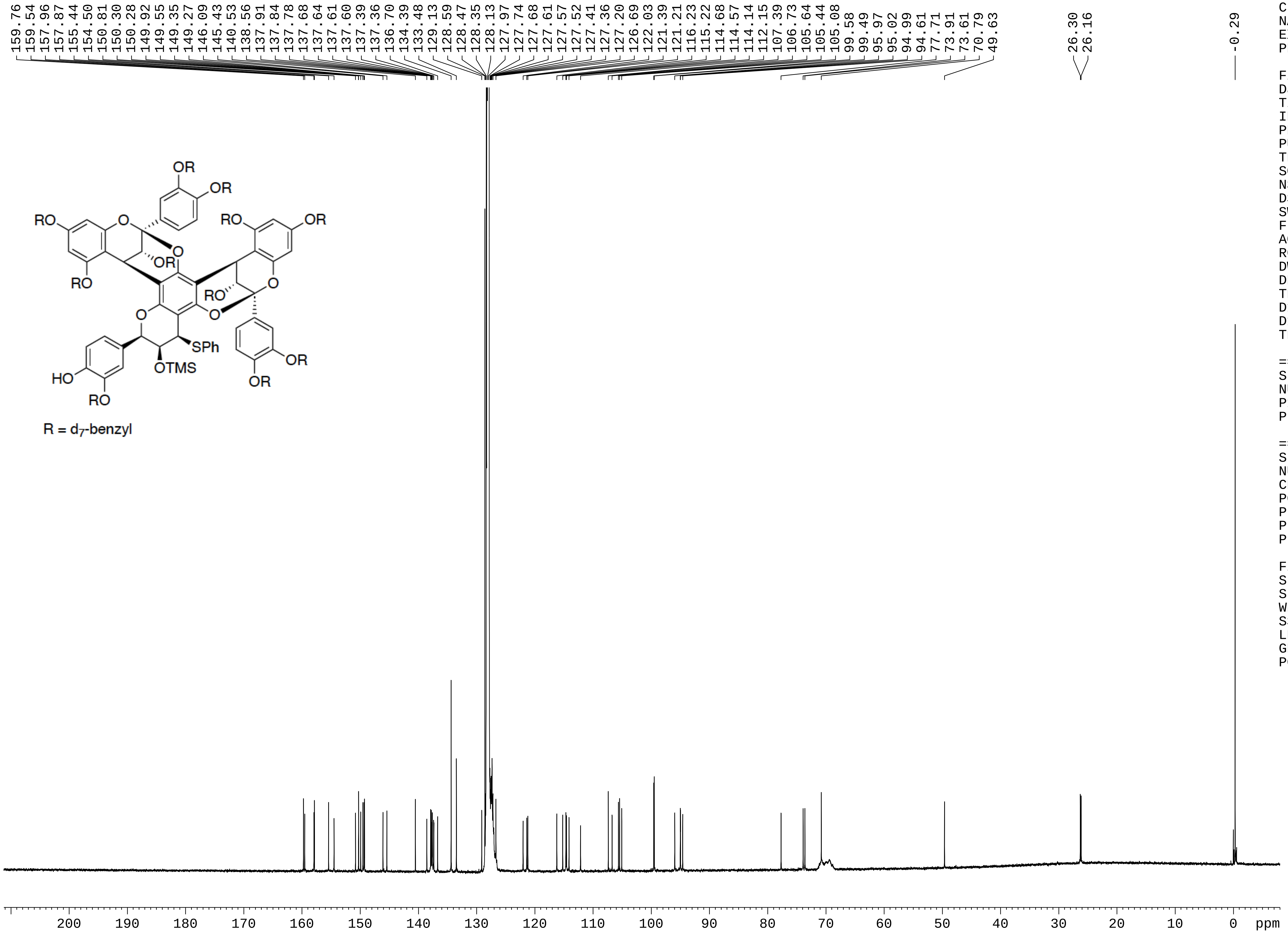

R = d_7-benzyl

Current Data Parameters
NAME VB-718
EXPNO 94
PROCNO 1

F2 - Acquisition Parameters
Date_ 20211019
Time 5.05
INSTRUM spect
PROBHD 5 mm CPPBBO BB
PULPROG zgpg30
TD 65536
SOLVENT C6D6
NS 8500
DS 4
SWH 36057.691 Hz
FIDRES 0.550197 Hz
AQ 0.9087659 sec
RG 175.56
DW 13.867 usec
DE 18.00 usec
TE 298.3 K
D1 2.00000000 sec
D11 0.03000000 sec
TD0 1

======== CHANNEL f1 ========
SFO1 150.9178981 MHz
NUC1 13C
P1 10.00 usec
PLW1 80.00000000 W

======== CHANNEL f2 ========
SFO2 600.1324005 MHz
NUC2 1H
CPDPRG[2 waltz16
PCPD2 70.00 usec
PLW2 13.43999958 W
PLW12 0.61714000 W
PLW13 0.31042001 W

F2 - Processing parameters
SI 32768
SF 150.9027539 MHz
WDW EM
SSB 0
LB 1.00 Hz
GB 0
PC 1.40

NOE of 22

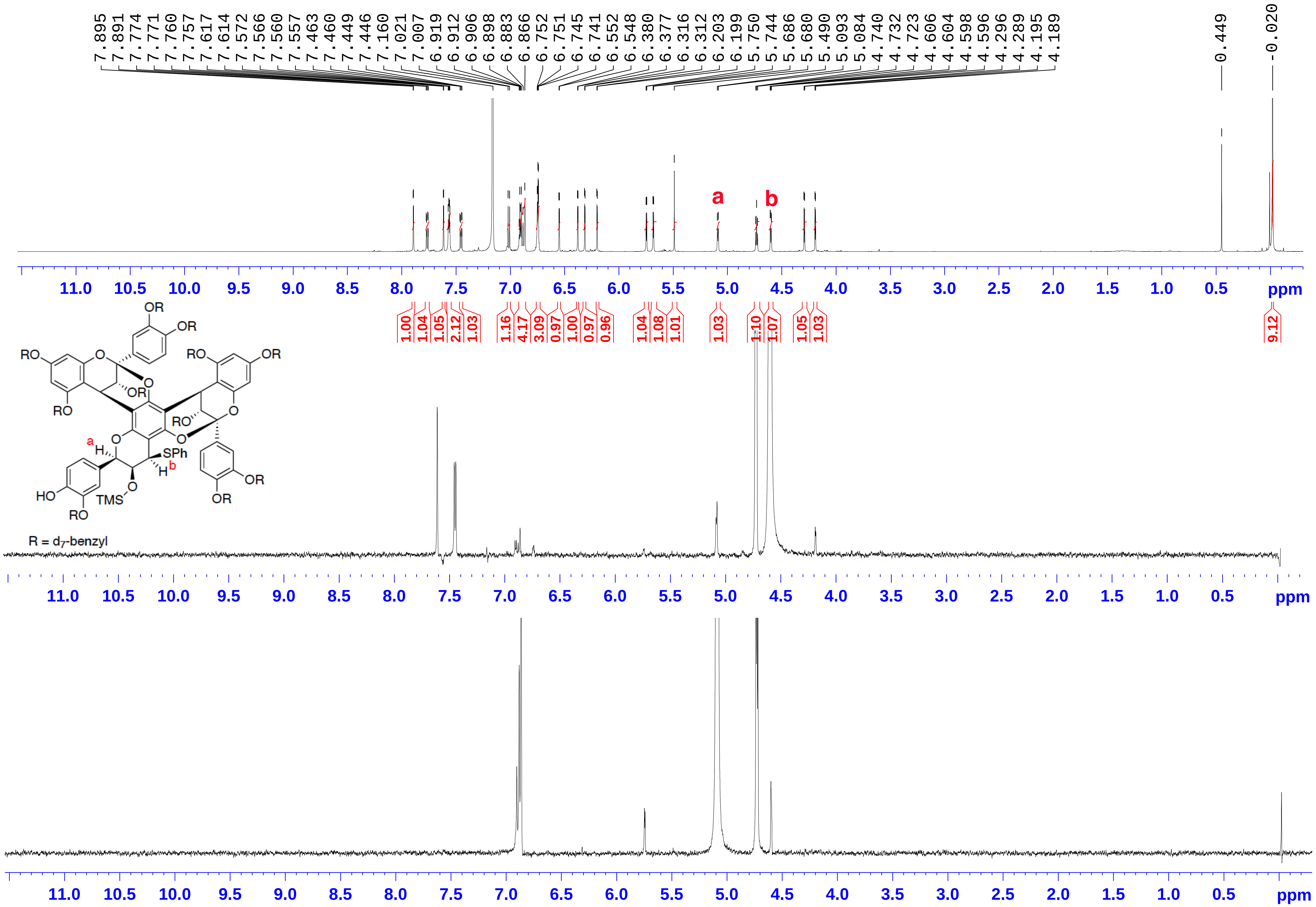

Current Data Parameters	
NAME	VB-718
EXPNO	90
PROCNO	1

F2 - Acquisition Parameters	
Date_	20211018
Time	19.48
INSTRUM	spect
PROBHD	5 mm CPPBBO BB
PULPROG	zg30
TD	65536
SOLVENT	C6D6
NS	16
DS	2
SWH	12019.230 Hz
FIDRES	0.183399 Hz
AQ	2.7262976 sec
RG	28.28
DW	41.600 usec
DE	10.00 usec
TE	298.1 K
D1	1.00000000 sec
TD0	1

======== CHANNEL f1 ========	
SFO1	600.1337060 MHz
NUC1	1H
P1	12.00 usec
PLW1	21.00000000 W

F2 - Processing parameters	
SI	65536
SF	600.1299927 MHz
WDW	EM
SSB	0
LB	0.30 Hz
GB	0
PC	1.00

^{1}H NMR of 25 (600MHz, $CDCl_3$)

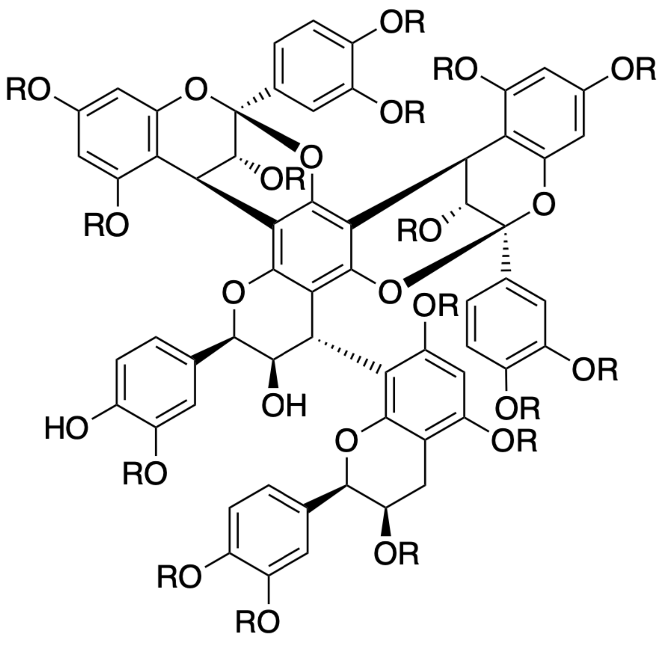

R = d_7-benzyl

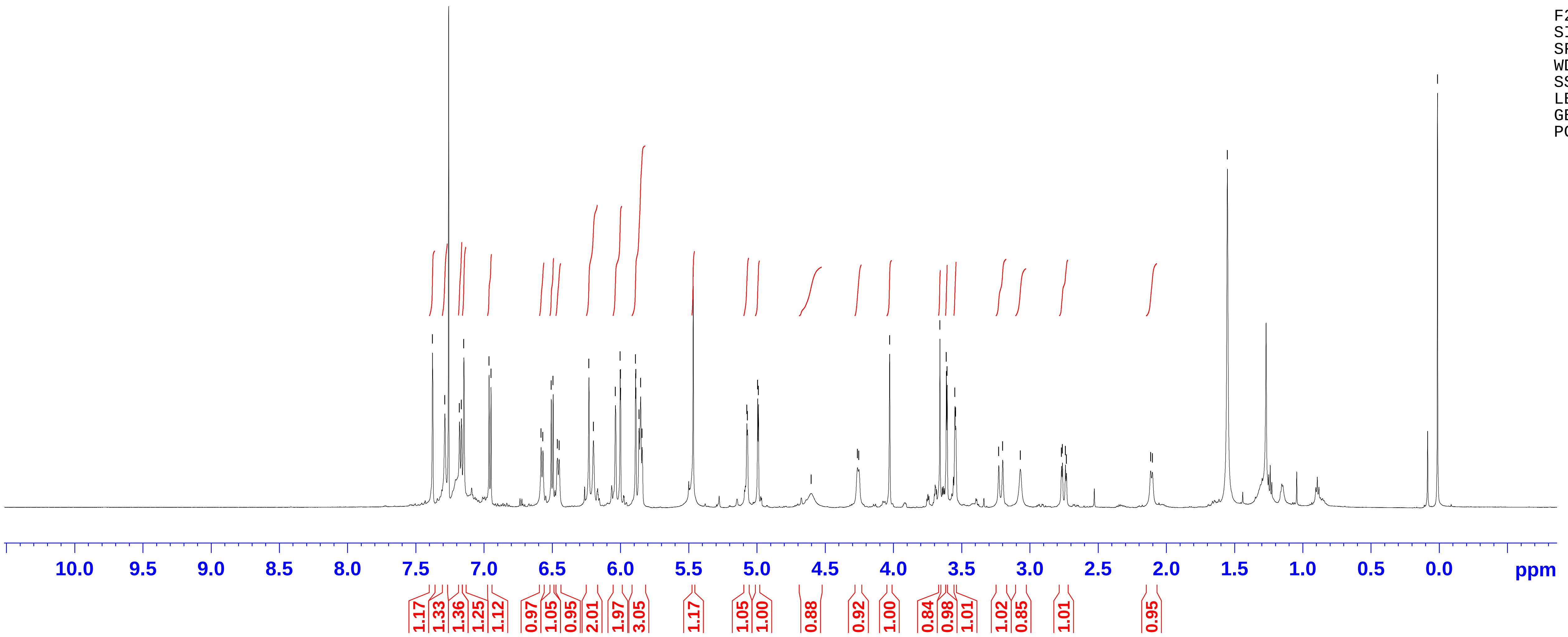


```
Current Data Parameters
NAME              VB-894
EXPNO                 41
PROCNO                 1

F2 - Acquisition Parameters
Date_           20211118
Time               19.32
INSTRUM            spect
PROBHD   5 mm CPPBBO BB
PULPROG             zg30
TD                 65536
SOLVENT            CDCl3
NS                   100
DS                     2
SWH            12019.230 Hz
FIDRES          0.183399 Hz
AQ             2.7262976 sec
RG                 31.94
DW                41.600 usec
DE                 10.00 usec
TE                 298.1 K
D1            1.00000000 sec
TD0                    1

======== CHANNEL f1 ========
SFO1         600.1337060 MHz
NUC1                  1H
P1                 12.00 usec
PLW1         21.00000000 W

F2 - Processing parameters
SI                 65536
SF           600.1300146 MHz
WDW                   EM
SSB      0
LB                  0.30 Hz
GB       0
PC                  1.00
```

^{13}C NMR of 25 (150MHz, $CDCl_3$)

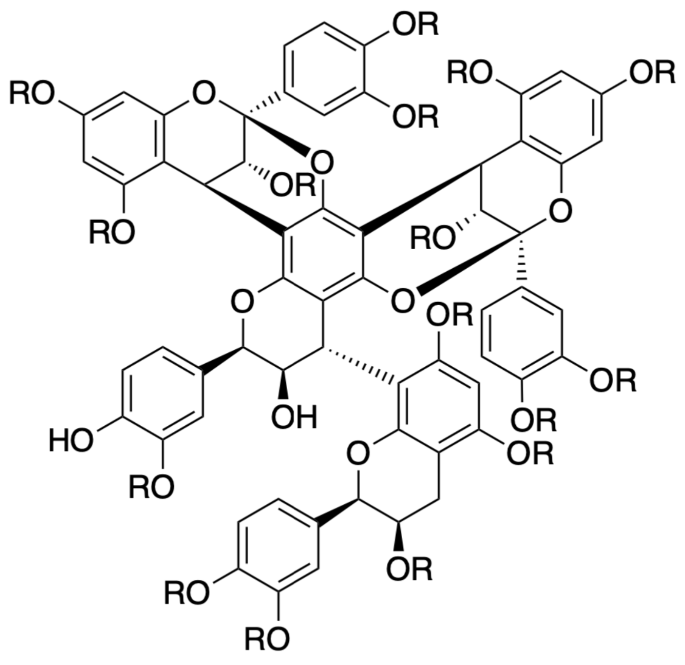

R = d_7-benzyl

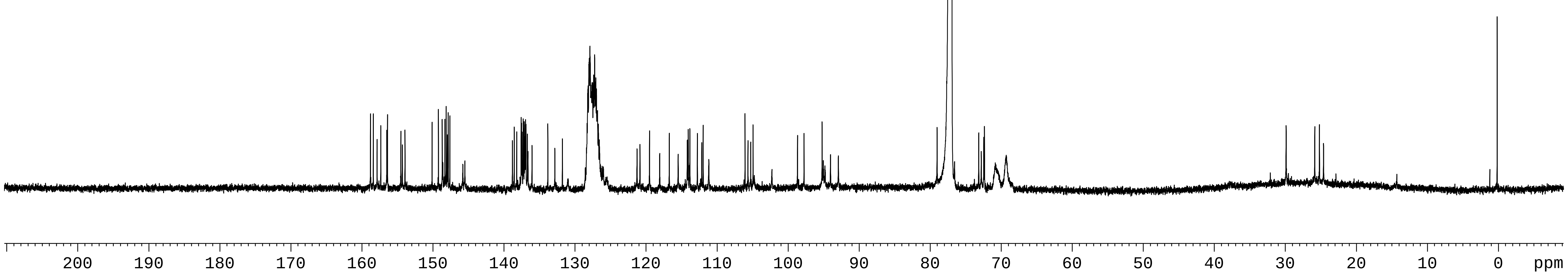

Current Data Parameters
NAME VB-894
EXPNO 13
PROCNO 1

F2 - Acquisition Parameters
Date_ 20211111
Time 7.57
INSTRUM spect
PROBHD 5 mm CPPBBO BB
PULPROG zgpg30
TD 65536
SOLVENT CDCl3
NS 12000
DS 4
SWH 36057.691 Hz
FIDRES 0.550197 Hz
AQ 0.9087659 sec
RG 175.56
DW 13.867 usec
DE 18.00 usec
TE 298.2 K
D1 2.00000000 sec
D11 0.03000000 sec
TD0 1

======== CHANNEL f1 ========
SFO1 150.9178981 MHz
NUC1 13C
P1 10.00 usec
PLW1 80.00000000 W

======== CHANNEL f2 ========
SFO2 600.1324005 MHz
NUC2 1H
CPDPRG[2 waltz16
PCPD2 70.00 usec
PLW2 13.43999958 W
PLW12 0.61714000 W
PLW13 0.31042001 W

F2 - Processing parameters
SI 32768
SF 150.9027832 MHz
WDW EM
SSB 0
LB 1.00 Hz
GB 0
PC 1.40

^{1}H NMR of 1 (600MHz, CD_3OD)

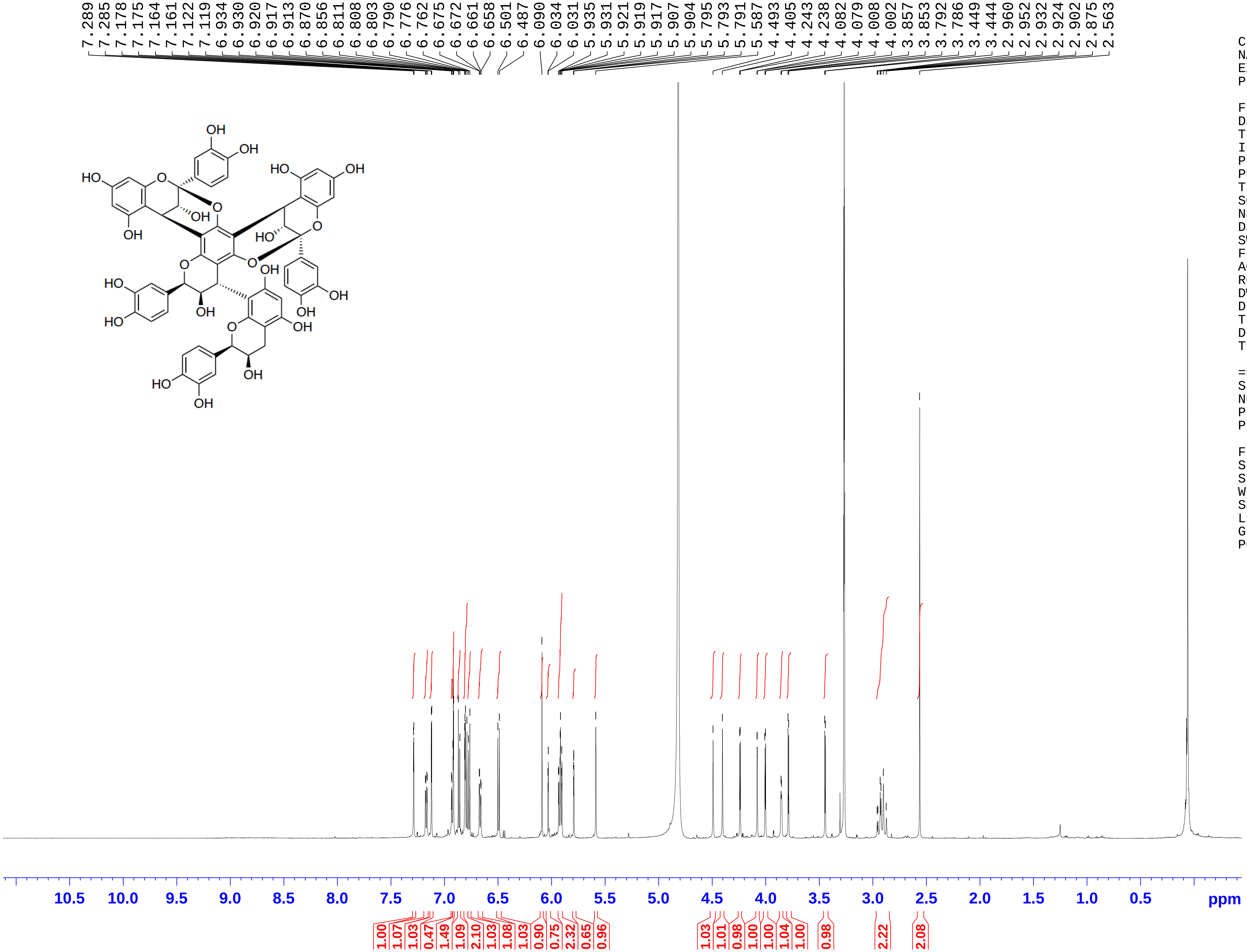

BRUKER

Current Data Parameters
NAME VB-809
EXPNO 11
PROCNO 1

F2 - Acquisition Parameters
Date_ 20210521
Time 20.01
INSTRUM spect
PROBHD 5 mm CPPBBO BB
PULPROG zg30
TD 65536
SOLVENT MeOD
NS 50
DS 2
SWH 12019.230 Hz
FIDRES 0.183399 Hz
AQ 2.7262976 sec
RG 31.94
DW 41.600 usec
DE 10.00 usec
TE 298.2 K
D1 1.00000000 sec
TD0 1

======== CHANNEL f1 ========
SFO1 600.1337060 MHz
NUC1 1H
P1 12.00 usec
PLW1 21.00000000 W

F2 - Processing parameters
SI 65536
SF 600.1300366 MHz
WDW EM
SSB 0
LB 0.30 Hz
GB 0
PC 1.00

^{13}C NMR of 1 (150MHz, CD_3OD)

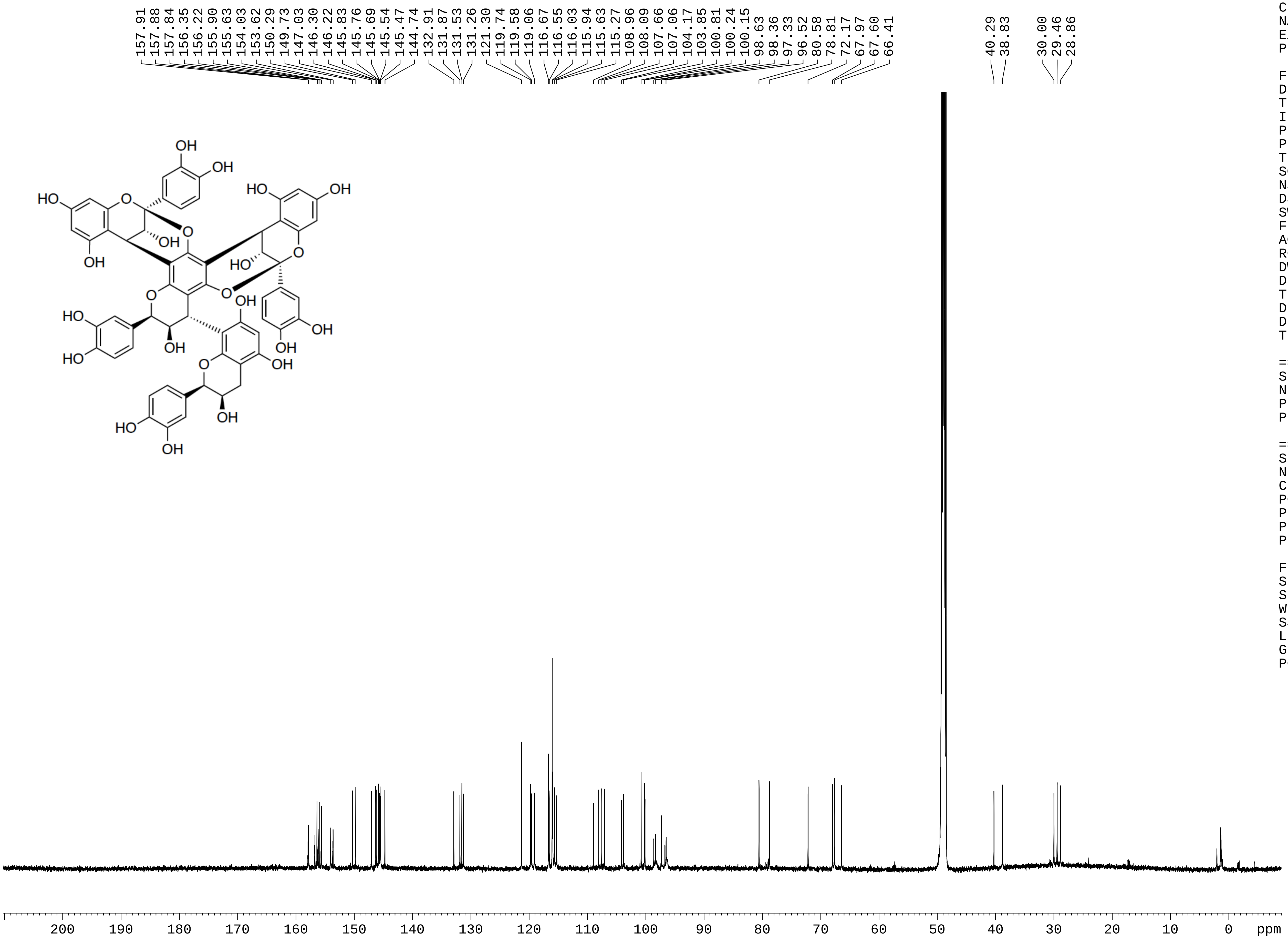

Current Data Parameters
NAME VB-809
EXPNO 12
PROCNO 1

F2 - Acquisition Parameters
Date_ 20210522
Time 4.51
INSTRUM spect
PROBHD 5 mm CPPBBO BB
PULPROG zgpg30
TD 65536
SOLVENT MeOD
NS 8192
DS 4
SWH 36057.691 Hz
FIDRES 0.550197 Hz
AQ 0.9087659 sec
RG 175.56
DW 13.867 usec
DE 18.00 usec
TE 298.1 K
D1 2.00000000 sec
D11 0.03000000 sec
TD0 1

======== CHANNEL f1 ========
SFO1 150.9178981 MHz
NUC1 13C
P1 10.00 usec
PLW1 80.00000000 W

======== CHANNEL f2 ========
SFO2 600.1324005 MHz
NUC2 1H
CPDPRG[2 waltz16
PCPD2 70.00 usec
PLW2 13.43999958 W
PLW12 0.61714000 W
PLW13 0.31042001 W

F2 - Processing parameters
SI 32768
SF 150.9026106 MHz
WDW EM
SSB 0
LB 1.00 Hz
GB 0
PC 1.40
